# Palladium-Catalyzed Carbonylative Sonogashira Coupling of Aryl Thianthrenium Salts with Arylalkynes

**DOI:** 10.1021/acs.orglett.4c02440

**Published:** 2024-07-18

**Authors:** Yan-Hua Zhao, Xing-Wei Gu, Xiao-Feng Wu

**Affiliations:** † Leibniz-Institut für Katalyse e.V., Albert-Einstein-Straße 29a, 18059 Rostock, Germany; ‡ Dalian National Laboratory for Clean Energy, Dalian Institute of Chemical Physics, Chinese Academy of Sciences, 116023 Dalian, Liaoning, China

## Abstract

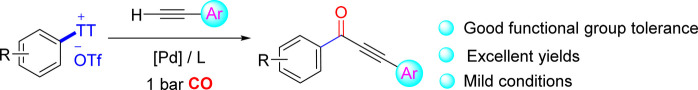

Alkynones are valuable compounds with applications in various areas. In this work, we developed an efficient carbonylation procedure for the carbonylative cross-coupling of aryl thianthrenium salts with aromatic alkynes. Various useful alkynones were produced in moderate to excellent yields under mild conditions. Notably, among the various tolerated functional groups, the bromide group can be maintained, which is ready for further coupling reactions.

Alkynones make up an essential class of building blocks, which are relatively common in many natural products,^1^ biologically active compounds,^2^ and pharmaceuticals^3^ and are crucial intermediates in the synthesis of heterocyclic compounds,^4^ including quinolones,^5^ pyrimidines,^6^ furans,^7^ pyrroles,^8^ pyrazoles,^9^ and flavonoids.^10^ In conventional methods, acetylenic ketones are usually prepared by the reaction of acyl halides with alkynyl organometallic reagents^11^ or terminal alkynes (Figure 1a).^12^ However, while acyl halides exhibit good reactivity in such transformations, their drawbacks of corrosiveness and sensitivity to moisture, as well as the poor substrate stability and narrow tolerance of functional groups that are usually associated with this method, limit the conventional method. Therefore, transition metal-catalyzed carbonylation of alkynes provides a viable alternative method for preparing alkynones.^13^

**Figure 1:**
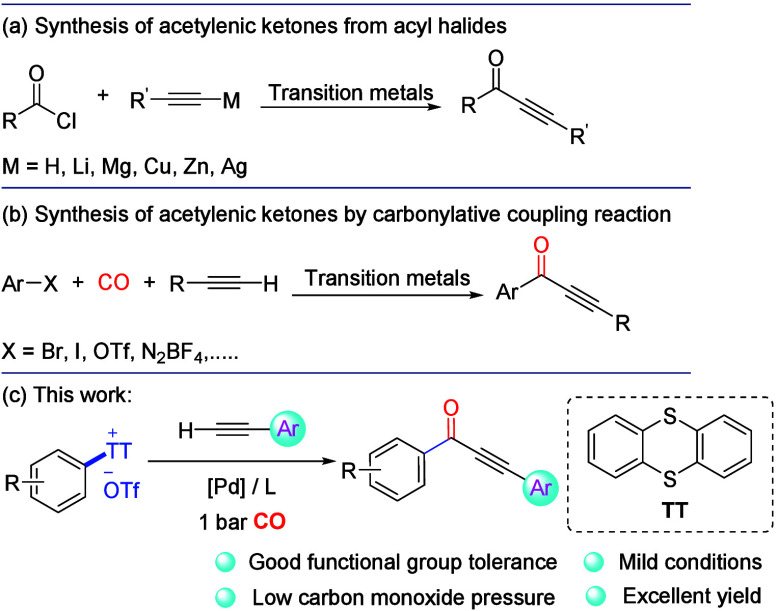
Synthetic strategies for alkynones.

Carbon monoxide (CO) is an important C1 synthon that is inexpensive and plentiful and has been widely used by synthetic chemists in carbonylation reactions.^14^ In the past few decades, the progress of transition metal-catalyzed carbonylative transformations has been outstanding, and a variety of carbonyl-containing compounds can be prepared directly by such transformations, including acetylenic ketones. Since the first report on palladium-catalyzed carbonylative Sonogashira coupling by Kobayashi and Tanaka in 1981,^15^ this transformation has been improved in a variety of ways. This transformation has also expanded the range of substrates available (Figure 1b), such as aryl triflates,^16^ aryl halides,^17^ and aryl triazenes.^18^ In 2011, Lee et al. reported a palladium-catalyzed synthesis of alkynyl carboxylic acids with aryl iodides to alkynyl aryl ketones under an atmospheric pressure of CO.^19^ In 2014, Li’s group reported the nonhomogeneous palladium-MOF-catalyzed carbonylation Sonogashira coupling of terminal alkynes with aryl iodides.^20^ Subsequently, Wu’s group reported an effective carbonylation cross-coupling of aryl diazonium salts with terminal alkynes using formic acid as a CO source and DCC as an activator.^21^

In recent years, the exploration of aryl thianthrenium salts in synthetic chemistry has attracted a great deal of attention.^22−25^ It was found that aryl thianthrenium salts are readily available and reactive electrophilic reagents. Additionally, compared with aryl halides, aryl thianthrenium salts can solve the problem of selective functionalization of aromatic C–H bonds. Hence, we hypothesize that the carbonylation of arenes and terminal alkynes via C(sp^2^)–H thioanthracenylation would be an interesting alternative route for the construction of alkynones (Figure 1c).

We started our study with aryl thianthrenium salt **1a** and phenylacetylene **2a** as model substrates to produce alkynyl ketone **3a** as the targeted product (Table 1). After a systematic investigation of the reaction parameters, the following optimal conditions were determined: aryl thianthrenium salts **1a** (1.5 equiv), aryl acetylenes **2a** (1.0 equiv), PdBr_2_ (2 mol %), PCy_3_ (6 mol %), K_3_PO_4_ (2.0 equiv), DMF (0.1 mol L^–1^), 80 °C, 15 h, atmospheric pressure of CO. The desired alkynone **3a** was obtained smoothly with a GC yield of 94% (isolated yield of 91%) (Table 1, entry 1). The desired alkynone **3a** could not be detected in the absence of the ligand, catalyst, or base (Table 1, entries 2–4). A decreased yield was obtained with a lower palladium precursor loading. Subsequently, we examined different catalysts, and moderate yields of **3a** were still obtained (Table 1, entries 5–7). Then, we investigated different ligands and found that monodentate phosphine ligands (such as PPh_3_ and BuPAd_2_) showed good reactivity, and the reaction can give the desired alkynone **3a** in good yields (Table 1, entries 8 and 9). Xantphos was also tested, but only a trace amount of the desired product was detected, perhaps because the size of the substrate cation cannot match the large bite angle of this ligand (Table 1, entry 10). Some other bases (such as Cs_2_CO_3_ and Et_3_N) were also tested but resulted in low yields of the desired product (Table 1, entries 11 and 12, respectively). Among the tested solvents, 1,4-dioxane can give a 90% yield of target product **3a** (Table 1, entry 13). However, when PhCF_3_ and DMC were used as solvents, the yield of the reaction decreased dramatically (Table 1, entries 14 and 15, respectively). The increased concentration of the reaction mixture induced a slight decrease in the yield (Table 1, entry 16). Finally, we reduced the amount of aryl thianthrenium salt **1a** with no obvious change in the yield (Table 1, entries 17 and 18), but we ultimately chose 1.5 equiv of **1a** for the subsequent substrate testing. It is worth mentioning that the internal alkyne from the direct Sonogashira coupling was the main side product of this reaction.

**Table 1:**
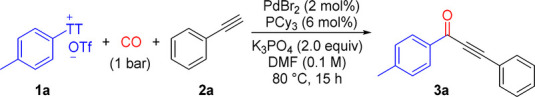
Optimization of the Reaction Conditions^a^

entry	variation from the standard conditions	yield (%)^b^
1	none	94 (91)^c^
2	no ligand	0
3	no catalyst	0
4	no base	0
5	Pd(OAc)_2_ instead of PdBr_2_	60
6	Pd(acac)_2_ instead of PdBr_2_	63
7	PdCl_2_ instead of PdBr_2_	85
8	PPh_3_ instead of PCy_3_	84
9	BuPAd_2_ instead of PCy_3_	88
10	Xantphos instead of PCy_3_	trace
11	Cs_2_CO_3_ instead of K_3_PO_4_	66
12	Et_3_N instead K_3_PO_4_	70
13	1.4-dioxane as the solvent	90
14	PhCF_3_ as the solvent	33
15	DMC as the solvent	54
16	0.2 mol L^–1^ DMF	80
17	1.0 equiv of **1a**	89
18	1.2 equiv of **1a**	90

a The reaction was conducted using **1a** (*x* mmol), **2a** (0.2 mmol), a catalyst (2 mol %), a ligand (6 mol %), a base (2.0 equiv), and CO (1 bar) at 80 °C for 15 h. Abbreviations: DMF, dimethylformamide; BuPAd_2_, di(1-adamantyl)-*n*-butylphosphine; DMC, dimethyl carbonate.

b Determined by GC with hexadecane as the internal standard.

c Isolated yield in parentheses.

After determining the optimal conditions, we initially investigated the substrate scope of a series of aryl thianthrenium salts (Scheme 1). Fortunately, a variety of aryl thianthrenium salts with different substituents could smoothly react under the standard conditions, and the corresponding alkynyl ketone products (**3a**–**3q**) were obtained in moderate to excellent yields. In the reaction system, an aryl thianthrenium salt without substituents on the aryl group or with monosubstituents (such as methoxy and methyl) afforded the target products (**3a**–**3c**) in excellent yields. In addition, the deuterated aryl thianthrenium salt was also compatible with this reaction system and afforded the corresponding deuterated alkynyl ketone in 93% yield (**3d**). Then monosubstituted aryl thianthrenium salts containing C(sp^2^)–X bonds (X = F, Cl, or Br) were investigated as the substrates, and the target products (**3e**–**3g**) were obtained in moderate to excellent yields. Notably, disubstituted substrates (**3h**–**3n**) and trisubstituted substrates (**3m**–**3q**) containing C(sp^2^)–X bonds (X = F, Cl, or Br) were similarly compatible with this reaction system and afforded the target products in good yields, which provide opportunities for subsequent structural modifications. Remarkably, the samples with multiple substitutions (**3h**–**3q**) prove the advantage of using aryl thianthrenium salts that are often difficult to obtain with other carbonylation methods.

**Scheme 1:**
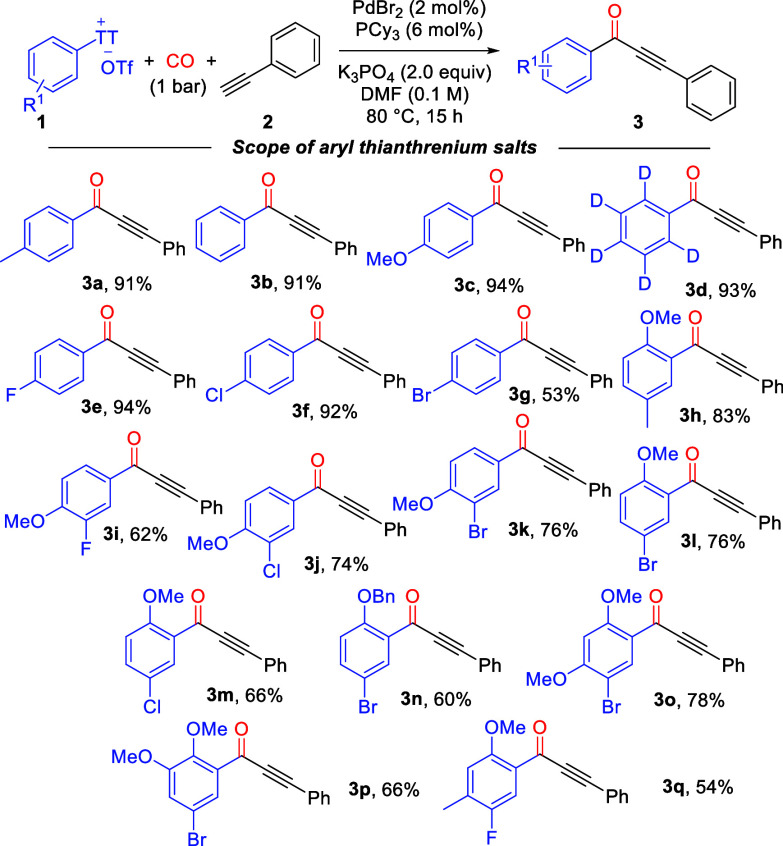
Substrate Scope of Aryl Thianthrennium Salts Reaction conditions: **1** (0.3 mmol), **2** (0.2 mmol), PdBr_2_ (2 mol %), PCy_3_ (6 mol %), K_3_PO_4_ (2.0 equiv), CO (1 bar), and DMF (0.1 M) at 80 °C for 15 h. Yields of isolated products.

Subsequently, we surveyed a variety of aryl acetylenes containing different functional groups (Scheme 2). Aryl acetylenes with electron-donating or electron-withdrawing groups all participated efficiently in the reaction, and the target products (**3r–3ah**) were synthesized in moderate to excellent yields. Noticeably, the reaction system has good functional group compatibility, and the electronic effects on the aromatics do not seem to affect the reaction results significantly; alkoxy (**3r**), alkyl (**3s**, **3w**, and **3x**), halogen (**3t–3v**, **3y**, and **3z**), trifluoromethyl (**3aa**), aldehyde (**3ae**), ester (**3af**), and ketone (**3ag**) were all tolerated well. The naphthyl motif was easily incorporated into the product (**3ab**). Various heterocycles, such as thiophene and pyridine, smoothly underwent this carbonylative Sonogashira coupling reaction and gave the desired products in good yields (**3ac** and **3ad**, respectively). However, aliphatic alkynes failed under the standard conditions, and only the direct Sonogashira coupling product was observed.

**Scheme 2:**
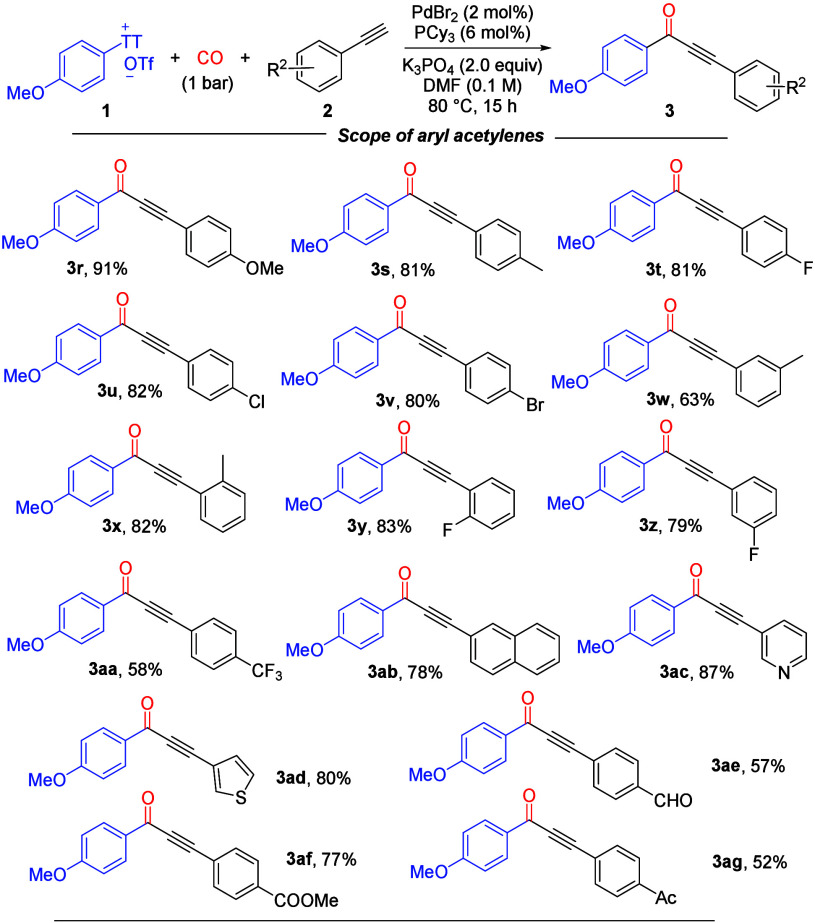
Substrate Scope of Aryl Acetylenes Reaction conditions: **1** (0.3 mmol), **2** (0.2 mmol), PdBr_2_ (2 mol %), PCy_3_ (6 mol %), K_3_PO_4_ (2.0 equiv), CO (1 bar), and DMF (0.1 M) at 80 °C for 15 h. Yields of isolated products.

Next, some mechanistic experiments were performed to understand the reaction mechanism. Under standard conditions, TEMPO (2 equiv) or BHT (1–3 equiv) was added to the reaction mixture, and the reaction still proceeded smoothly, providing the desired alkynone in 34–89% yields (Scheme 3a). Meanwhile, the addition of 1,1-diphenylethylene (1,1-DPE) to the standard reaction mixture afforded the desired acetylenic ketone **3a** in 80% yield (Scheme 3b). Hence, the possibility that the reaction involves a free radical intermediate can be fully excluded.

**Scheme 3:**
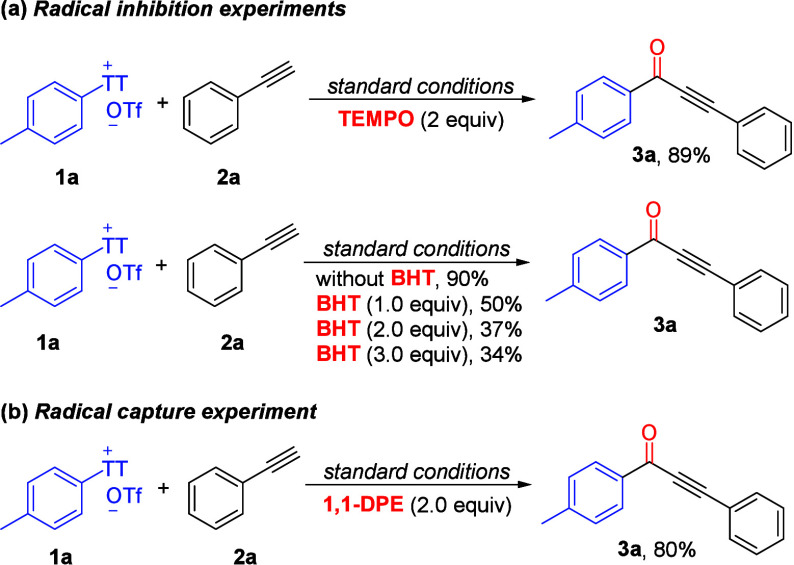
Control Experiments

According to the results presented above and related reports,^22−25^ a possible reaction mechanism is proposed (Scheme 4). First, active Pd(0) complex **A** was generated from the palladium precursor, after which complex **A** underwent oxidative addition with an aryl thianthrenium salt to generate palladium complex **B**. Next, CO will coordinate and insert into complex **B** to form acyl palladium intermediate **C**. Then, aryl palladium **C** reacts with the terminal alkene in the presence of a base to form alkynyl palladium complex **D**. Finally, complex **D** undergoes reductive elimination to deliver the final product while regenerating complex **A** for the next catalytic cycle.

**Scheme 4:**
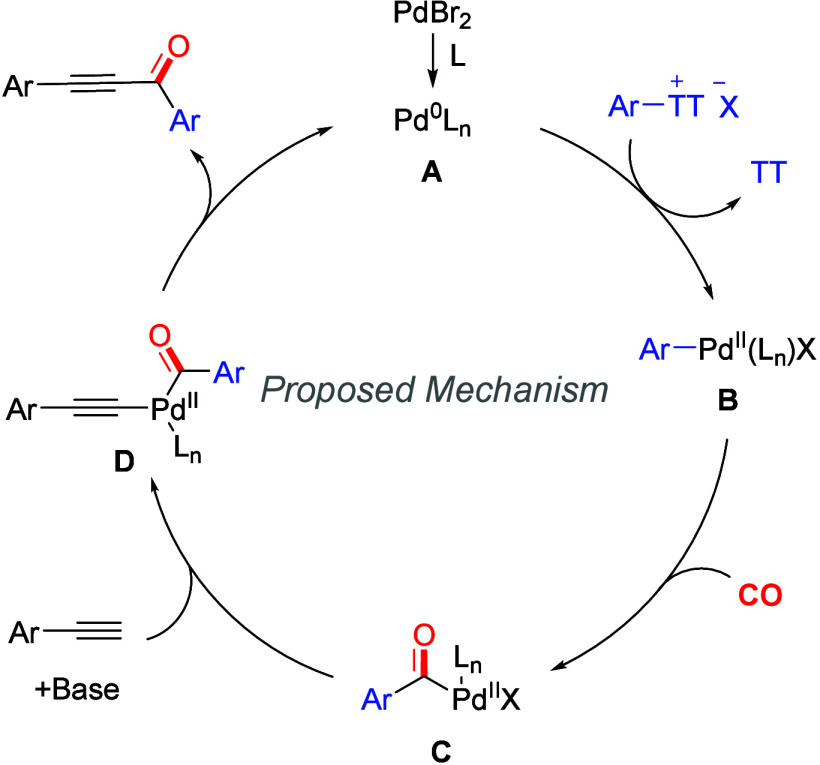
Proposed Mechanism

In conclusion, we developed a palladium-catalyzed carbonylative Sonogashira coupling reaction of arylthioanthracene salts with terminal alkynes under an atmospheric pressure of CO. The used arylthioanthracene salts are inexpensive and readily available; a series of alkynones were obtained in moderate to good yields with excellent functional group compatibility under mild conditions.

## Data Availability

The data underlying this study are available in the published article and its Supporting Information.

## Supporting Information Available

General comments, general procedure, optimization details, analytical data, and NMR spectra (PDF)
