# Supplementary material for: Palladium-Catalyzed Carbonylative Sonogashira Coupling of Aryl Thianthrenium Salts with Arylalkynes
Source: Org Lett. 2024 Jul 18;26(30):6507–11. doi: 10.1021/acs.orglett.4c02440 (PMC11301662; doi:10.1021/acs.orglett.4c02440)
Supplement: Supplementary file 1 — ol4c02440_si_001.pdf [file ol4c02440_si_001.pdf]

Supporting Information

Palladium-Catalyzed Carbonylative Sonogashira Coupling of Aryl Thianthrenium Salts with Arylalkynes

Yan-Hua Zhao†a, Xing-Wei Gu†a, Xiao-Feng Wu*a,b

a. Leibniz-Institut für Katalyse e.V., Albert-Einstein-Straße 29a, 18059 Rostock, Germany

b. Dalian National Laboratory for Clean Energy, Dalian Institute of Chemical Physics, Chinese Academy of Sciences, 116023 Dalian, Liaoning, China, e-mail: xwu2020@dicp.ac.cn

Contents

1. General Experimental S2

2. General procedure for the synthesis of aryl sulfonium salts. S2

3. General Procedure for the carbonylation. S3

4. Characterization and procedure of the products S4

5. Control experiments S16

6. Reference S18

7. NMR spectra of products S19

1. General Experimental

Reagents and solvents: Unless otherwise noted, the chemicals were commercially available from Sigma-Aldrich, TCI or Alfa Aesar and were used without further purification. Dioxane bought from Alfa Aesar, HPLC grade, 99% min, packaged under argon in resealable ChemSeal bottles. The reaction does not require the glovebox.

Purification: The products were isolated from the reaction mixture by column chromatography on silica gel 60, 0.063-0.2 mm, 70-230 mesh (Merck). Gradient flash chromatography was conducted eluting with PE/EA, PE refers to pentane and EA refers to ethyl acetate, they were listed as volume/volume ratios.

Data collection: GC-yields were calculated using hexadecane as internal standard. GC analysis was performed on an Agilent HP-7890A instrument with FID detector and HP-5 capillary column (polydimethylsiloxane with 5% phenyl groups, 30 m, 0.32 mm i.d., 0.25 µm film thickness) using argon as carrier gas. High resolution mass spectra (HRMS) were recorded on Agilent 6210. NMR spectra were recorded on Bruker Avance 300 and Bruker ARX 400 spectrometers. Chemical shifts (ppm) are given relative to solvent: references for $CDCl_3$ were 7.26 ppm (^1H NMR) and 77.00 ppm (^{13}C NMR). All measurements were carried out at room temperature unless otherwise stated.

2. General procedure for the synthesis of aryl sulfonium salts.

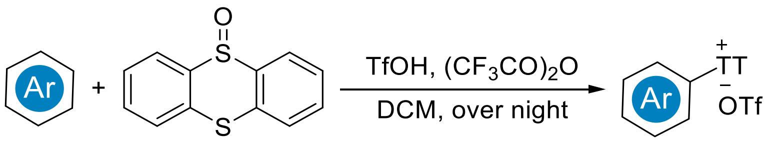

Aryl thianthrenium triflates were prepared by known literature procedure.[1] Thianthrene S-oxide (1.1 equiv.), arene (1mmol, 1 equiv.) and DCM (0.2M) were added to a 100 mL Schlenk tube under a nitrogen atmosphere, equipped with a magnetic stir bar. The reaction mixture was then cooled to -40 °C and stirred at this temperature. Tf_2O (1.2 equiv.) was added dropwise. The reaction mixture was stirred at -40 °C for 30 min, and then allowed to stir at room temperature for 12 h, neutralized by a saturated aqueous $NaHCO_3$ solution, and extracted with DCM. The combined organic layers were dried over anhydrous Na_2SO_4 and concentrated to dryness under reduced pressure. The crude product was purified by crystallization from DCM / diethyl ether system to afford the aryl thianthrenium triflate.

3. General Procedure for the carbonylation.

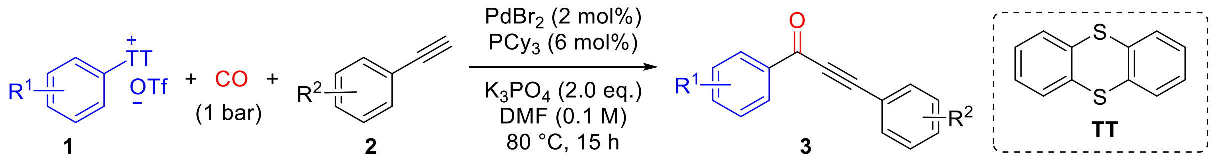

A 4 mL snap vial was charged with $PdBr_2$ (0.004 mmol, 2 mol %), PCy_3 (0.012 mmol, 6 mol%), K_3PO_4 (0.4 mmol, 2.0 equiv.), aryl sulfonium salts (0.3 mmol, 1.5 equiv.) and closed with a rubber-based septum. The vial was evacuated and backfilled with argon. Degassed DMF (2.0 mL) and aryl acetylenes (0.2 mmol, 1.0 equiv.) were added via syringe. The vial was then connected to atmosphere with a cannula and transferred into a 300 mL Parr 4560 series autoclave, under argon counterflow. The closed autoclave was flushed three times with nitrogen (~ 5 bar), three times with CO (~ 5 bar), and 1 bar of carbon monoxide (measured by pressure meter) was charged. The autoclave was then placed into an aluminum block on a magnetic stirrer. The reaction mixture was stirred (500 rpm) at 80 °C (aluminum block) 15 h. The crude product was purified by silica gel chromatography (pentane/EA) to afford the corresponding product.

3 mmol scale: a 12 mL snap vial was charged with $PdBr_2$ (2 mol %), PCy_3 (6 mol%), K_3PO_4 (2.0 equiv.), aryl sulfonium salts (1.5 equiv.) and closed with a rubber-based septum. The vial was evacuated and backfilled with argon. Degassed DMF (8.0 mL) and phenyl acetylene (3 mmol, 1.0 equiv.) were added via syringe. The vial was then connected to atmosphere with a cannula and transferred into a 300 mL Parr 4560 series autoclave, under argon counterflow. The closed autoclave was flushed three times with nitrogen (~ 5 bar), three times with CO (~ 5 bar), and 1 bar of carbon monoxide (measured by pressure meter) was charged. The autoclave was then placed into an aluminum block on a magnetic stirrer. The reaction mixture was stirred (500 rpm) at 80 °C (aluminum block) 15 h. The crude product was purified by silica gel chromatography (pentane/EA) to afford the corresponding product **3c** in 64% yield (453.2 mg).

4. Characterization and procedure of the products

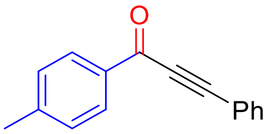

3a

3-phenyl-1-(*p*-tolyl) prop-2-yn-1-one (3a)[2]

White solid (40.1 mg, 91% yield), purified by column chromatography (SiO_2, Pentane/EA= 50:1).

^{1}H NMR (300 MHz, $CDCl_3$) δ 8.03 (d, J = 8.3 Hz, 2H), 7.65 – 7.55 (m, 2H), 7.43 – 7.28 (m, 3H), 7.22 (d, J = 8.0 Hz, 2H), 2.36 (s, 3H).

^{13}C NMR (75 MHz, $CDCl_3$) δ 177.7, 145.2, 134.6, 133.0, 130.6, 129.7, 129.3, 128.6, 120.2, 92.5, 86.9, 21.8.

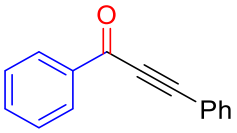

3b

1,3-diphenylprop-2-yn-1-one (3b)[2]

Colorless oil (37.4 mg, 91% yield), purified by column chromatography (SiO_2, Pentane/EA= 50:1).

^{1}H NMR (300 MHz, $CDCl_3$) δ 8.20 – 8.09 (m, 2H), 7.65 – 7.50 (m, 3H), 7.48 – 7.30 (m, 5H).

^{13}C NMR (75 MHz, $CDCl_3$) δ 178.0, 136.8, 134.1, 133.0, 130.8, 129.5, 128.6, 128.6, 120.1, 93.1, 86.8.

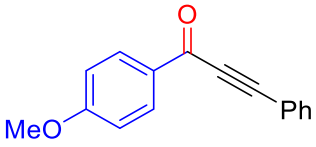

3c

1-(4-methoxyphenyl)-3-phenylprop-2-yn-1-one (3c)[2]

White solid (44.4 mg, 94% yield), purified by column chromatography (SiO_2, Pentane/EA= 20:1).

^{1}H NMR (300 MHz, $CDCl_3$) δ 8.25 – 8.13 (m, 1H), 7.72 – 7.61 (m, 1H), 7.53 – 7.33 (m, 2H), 7.04 – 6.92 (m, 1H), 3.89 (s, 1H).

^{13}C NMR (75 MHz, $CDCl_3$) δ 176.7, 164.5, 133.0, 132.0, 130.6, 130.3, 128.7, 120.4, 113.9, 92.3, 86.9, 55.6.

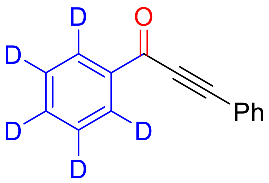

3d

3-phenyl-1-(phenyl-d_5) prop-2-yn-1-one (3d)

Colorless oil (39.3 mg, 93% yield), purified by column chromatography (SiO_2, Pentane/EA= 50:1).

^{1}H NMR (300 MHz, $CDCl_3$) δ 7.75 – 7.64 (m, 2H), 7.57 – 7.33 (m, 3H).

^{13}C NMR (75 MHz, $CDCl_3$) δ 178.0, 133.0, 130.8, 128.7, 120.1, 93.0, 86.9.

HRMS (ESI-TOF): m/z calcd. for $C_{15}H_6D_5O$ [M+H $^+$] 212.1118, found 212.1121.

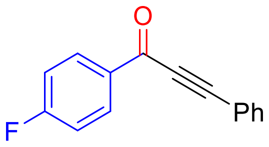

3e

1-(4-fluorophenyl)-3-phenylprop-2-yn-1-one (3e)[3]

White solid (42.2 mg, 94% yield), purified by column chromatography (SiO_2, Pentane/EA= 50:1).

^{1}H NMR (300 MHz, $CDCl_3$) δ 8.23 – 8.10 (m, 2H), 7.65 – 7.54 (m, 2H), 7.48 – 7.28 (m, 3H), 7.18 – 7.04 (m, 2H).

^{13}C NMR (75 MHz, $CDCl_3$) δ 176.3, 168.1, 164.7, 133.4 (d, J = 2.7 Hz), 133.0, 132.2 (d, J = 9.7 Hz), 130.9, 128.7, 119.9, 115.8 (d, J = 22.2 Hz), 93.3, 86.6.

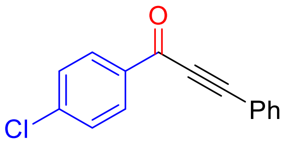

3f

1-(4-chlorophenyl)-3-phenylprop-2-yn-1-one (3f)[3]

White solid (44.5 mg, 92% yield), purified by column chromatography (SiO_2, Pentane/EA= 50:1).

^{1}H NMR (300 MHz, $CDCl_3$) δ 8.12 – 8.01 (m, 2H), 7.65 – 7.54 (m, 2H), 7.45 – 7.30 (m, 5H).

^{13}C NMR (75 MHz, $CDCl_3$) δ 176.6, 140.7, 135.2, 133.1, 130.9, 130.8, 129.0, 128.7, 119.8, 93.6, 86.5.

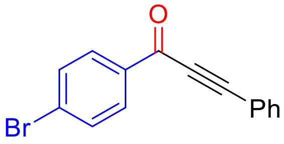

3g

1-(4-bromophenyl)-3-phenylprop-2-yn-1-one (3g)[3]

White solid (30.5 mg, 53% yield), purified by column chromatography (SiO_2, Pentane/EA= 50:1).

^{1}H NMR (400 MHz, $CDCl_3$) δ 8.05 – 7.94 (m, 2H), 7.65 – 7.54 (m, 4H), 7.45 – 7.33 (m, 3H).

^{13}C NMR (101 MHz, $CDCl_3$) δ 176.8, 135.7, 133.1, 132.0, 131.0, 130.9, 129.5, 128.7, 119.8, 93.7, 86.5.

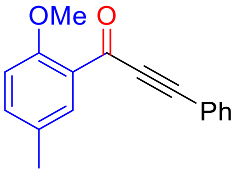

3h

1-(2-methoxy-5-methylphenyl)-3-phenylprop-2-yn-1-one (3h)[4]

Colorless oil (41.3 mg, 83% yield), purified by column chromatography (SiO_2, Pentane/EA= 20:1).

^{1}H NMR (300 MHz, $CDCl_3$) δ 7.81 – 7.73 (m, 1H), 7.60 – 7.49 (m, 2H), 7.43 – 7.23 (m, 4H), 6.83 (d, J = 8.5 Hz, 1H), 3.85 (s, 3H), 2.26 (s, 2H).

^{13}C NMR (75 MHz, $CDCl_3$) δ 176.8, 157.9, 135.6, 132.9, 132.5, 130.3, 129.6, 128.5, 126.4, 120.7, 112.2, 91.5, 89.3, 56.0, 20.3.

HRMS (ESI-TOF): m/z calcd. for $C_{17}H_{15}O_2$ [M+H $^+$] 251.1067, found 251.1073.

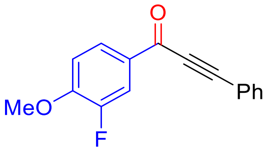

3i

1-(3-fluoro-4-methoxyphenyl)-3-phenylprop-2-yn-1-one (3i)

Yellow oil (31.4 mg, 62% yield), purified by column chromatography (SiO_2, Pentane/EA= 20:1).

^{1}H NMR (300 MHz, $CDCl_3$) δ 8.1 – 8.0 (m, 1H), 7.91 (dd, J = 11.6, 2.1 Hz, 1H), 7.72 – 7.62 (m, 2H), 7.51 – 7.37 (m, 3H), 7.05 (dd, J = 8.6, 8.0 Hz, 1H), 3.98 (s, 3H).

^{13}C NMR (75 MHz, $CDCl_3$) δ 175.7, 153.2 (d, J = 47.1 Hz), 151.5 (d, J = 190.4 Hz), 133.0, 130.8, 130.4 (d, J = 5.7 Hz), 128.7, 127.3 (d, J = 3.2 Hz), 120.0, 116.6 (d, J = 19.3 Hz), 112.4 (d, J = 1.9 Hz), 92.9, 86.5, 56.4.

^{19}F NMR (282 MHz, $CDCl_3$) δ -133.9 (dd, J = 11.5, 7.9 Hz).

HRMS (ESI-TOF): m/z calcd. for $C_{16}H_{12}FO_2$ [M+H $^+$] 255.0816, found 255.0823.

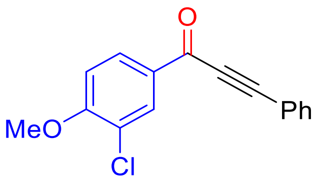

3j

1-(3-chloro-4-methoxyphenyl)-3-phenylprop-2-yn-1-one (3j)

Yellow oil (39.8 mg, 74% yield), purified by column chromatography (SiO_2, Pentane/EA= 20:1).

^{1}H NMR (300 MHz, $CDCl_3$) δ 8.14 (d, *J* = 2.2 Hz, 1H), 8.05 (dd, *J* = 8.6, 2.2 Hz, 1H), 7.65 – 7.54 (m, 2H), 7.45 – 7.29 (m, 3H), 6.94 (d, *J* = 8.6 Hz, 1H), 3.91 (s, 3H).

^{13}C NMR (75 MHz, $CDCl_3$) δ 175.5, 159.6, 133.0, 131.5, 130.8, 130.6, 130.2, 128.7, 123.0, 120.0, 111.3, 93.1, 86.4, 56.5.

HRMS (ESI-TOF): m/z calcd. for $C_{16}H_{12}ClO_2$ [M+H $^+$] 271.0521, found 271.0527.

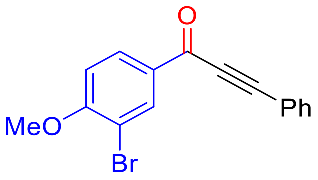

3k

1-(3-bromo-4-methoxyphenyl)-3-phenylprop-2-yn-1-one (3k)

Yellow oil (47.9 mg, 76% yield), purified by column chromatography (SiO_2, Pentane/EA= 20:1).

^{1}H NMR (300 MHz, $CDCl_3$) δ 8.31 (d, *J* = 2.1 Hz, 1H), 8.08 (dd, *J* = 8.6, 2.2 Hz, 1H), 7.64 – 7.53 (m, 2H), 7.46 – 7.31 (m, 3H), 6.90 (d, *J* = 8.7 Hz, 1H), 3.90 (s, 3H).

^{13}C NMR (75 MHz, $CDCl_3$) δ 175.4, 160.4, 134.7, 133.0, 131.1, 130.9, 130.8, 128.7, 120.0, 112.0, 111.1, 93.1, 86.4, 56.6.

HRMS (ESI-TOF): m/z calcd. for $C_{16}H_{12}BrO_2$ [M+H $^+$] 315.0015, found 315.0023.

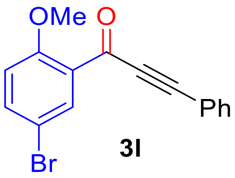

3l

1-(5-bromo-2-methoxyphenyl)-3-phenylprop-2-yn-1-one (3l)[5]

White solid (47.6 mg, 76% yield), purified by column chromatography (SiO_2, Pentane/EA= 20:1).

^{1}H NMR (400 MHz, $CDCl_3$) δ 8.13 (d, *J* = 2.6 Hz, 1H), 7.67 – 7.57 (m, 3H), 7.51 – 7.36 (m, 3H), 6.91 (d, *J* = 8.9 Hz, 1H), 3.94 (s, 3H).

^{13}C NMR (101 MHz, $CDCl_3$) δ 175.1, 158.7, 137.3, 134.6, 133.0, 130.7, 128.6, 128.1, 120.3, 114.1, 112.5, 92.6, 88.8, 56.2.

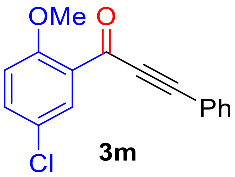

3m

1-(5-chloro-2-methoxyphenyl)-3-phenylprop-2-yn-1-one (3m)[5]

White solid (35.9 mg, 66% yield), purified by column chromatography (SiO_2, Pentane/EA= 20:1).

^{1}H NMR (300 MHz, $CDCl_3$) δ 7.92 (d, J = 2.7 Hz, 1H), 7.61 – 7.50 (m, 2H), 7.45 – 7.26 (m, 5H), 6.88 (d, J = 8.9 Hz, 1H), 3.87 (s, 3H).

^{13}C NMR (75 MHz, $CDCl_3$) δ 175.2, 158.3, 134.4, 133.0, 131.7, 130.7, 128.6, 127.7, 125.5, 120.3, 113.8, 92.5, 88.8, 56.2.

HRMS (ESI-TOF): m/z calcd. for $C_{16}H_{12}ClO_2$ [M+H $^+$] 271.0521, found 271.0529.

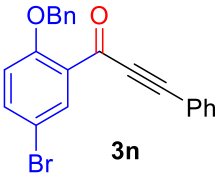

3n

1-(2-(benzyloxy)-5-bromophenyl)-3-phenylprop-2-yn-1-one (3n)

Yellow oil (47.1 mg, 60% yield), purified by column chromatography (SiO_2, Pentane/EA= 20:1).

^{1}H NMR (300 MHz, $CDCl_3$) δ 8.02 (d, J = 2.6 Hz, 1H), 7.48 (dd, J = 8.9, 2.6 Hz, 1H), 7.42 – 7.12 (m, 10H), 6.85 (d, J = 8.9 Hz, 1H), 5.12 (s, 2H).

^{13}C NMR (75 MHz, $CDCl_3$) δ 175.2, 157.7, 137.2, 135.7, 134.3, 132.9, 130.5, 128.7, 128.6, 128.4, 128.1, 127.2, 120.2, 115.4, 113.0, 92.9, 89.2, 71.0.

HRMS (ESI-TOF): m/z calcd. for $C_{22}H_{16}BrO_2$ [M+H $^+$] 391.0328, found 391.0336.

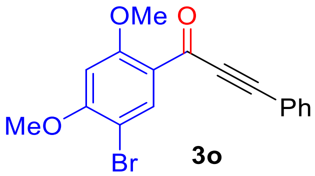

3o

1-(5-bromo-2,4-dimethoxyphenyl)-3-phenylprop-2-yn-1-one (3o)

Yellow oil (53.6 mg, 78% yield), purified by column chromatography (SiO_2, Pentane/EA= 20:1).

^{1}H NMR (300 MHz, $CDCl_3$) δ 8.22 (s, 1H), 7.58 – 7.52 (m, 2H), 7.44 – 7.25 (m, 3H), 6.41 (s, 1H), 3.91 (s, 6H).

^{13}C NMR (75 MHz, $CDCl_3$) δ 173.9, 161.5, 161.2, 137.1, 133.0, 130.5, 128.6, 120.7, 120.7, 102.3, 96.3, 91.7, 88.9, 56.6, 56.3.

HRMS (ESI-TOF): m/z calcd. for $C_{17}H_{14}BrO_3$ [M+H $^+$] 345.0121, found 345.0124.

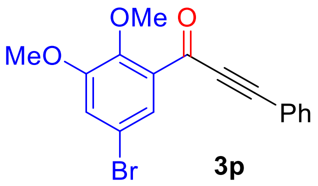

3p

1-(5-bromo-2,3-dimethoxyphenyl)-3-phenylprop-2-yn-1-one (3p)

Yellow oil (45.4 mg, 66% yield), purified by column chromatography (SiO_2, Pentane/EA= 20:1).

^{1}H NMR (300 MHz, $CDCl_3$) δ 7.62 – 7.50 (m, 3H), 7.48 – 7.25 (m, 3H), 7.05 (s, 1H), 3.88 (s, 3H), 3.87 (s, 3H).

^{13}C NMR (75 MHz, $CDCl_3$) δ 175.9, 152.8, 147.9, 132.9, 130.8, 129.2, 128.7, 120.2, 117.3, 115.1, 114.3, 93.9, 88.0, 56.4, 56.1.

HRMS (ESI-TOF): m/z calcd. for $C_{17}H_{14}BrO_3$ [M+H $^+$] 345.0121, found 345.0130.

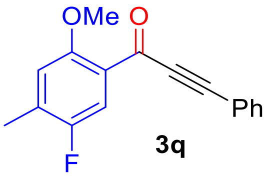

3q

1-(5-fluoro-2-methoxy-4-methylphenyl)-3-phenylprop-2-yn-1-one (3q)

Yellow oil (29.1 mg, 54% yield), purified by column chromatography (SiO_2, Pentane/EA= 20:1).

^{1}H NMR (300 MHz, $CDCl_3$) δ 7.72 (d, J = 9.8 Hz, 1H), 7.66 – 7.57 (m, 2H), 7.50 – 7.35 (m, 3H), 6.81 (d, J = 5.9 Hz, 1H), 3.93 (s, 3H), 2.33 (d, J = 1.8 Hz, 1H).

^{13}C NMR (75 MHz, $CDCl_3$) δ 175.0 (d, J = 2.0 Hz), 155.9 (d, J = 1.9 Hz), 154.8 (d, J = 239.1 Hz), 132.9, 132.9 (d, J = 19.1 Hz), 130.5, 128.6, 125.0 (d, J = 6.0 Hz), 120.5, 117.8 (d, J = 25.2 Hz), 115.1 (d, J = 4.3 Hz), 91.8, 88.9, 56.4, 15.3 (d, J = 3.0 Hz).

^{19}F NMR (282 MHz, $CDCl_3$) δ -127.2 – -127.4 (m).

HRMS (ESI-TOF): m/z calcd. for $C_{17}H_{14}FO_2$ [M+H $^+$] 269.0973, found 269.0981.

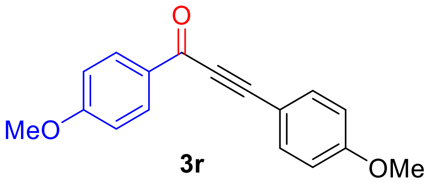

3r

1,3-bis(4-methoxyphenyl) prop-2-yn-1-one (3r)[2]

White solid (48.2 mg, 91% yield), purified by column chromatography (SiO_2, Pentane/EA= 20:1).

^{1}H NMR (300 MHz, $CDCl_3$) δ 8.16 – 8.04 (m, 2H), 7.59 – 7.48 (m, 2H), 6.94 – 6.78 (m, 4H), 3.80 (s, 3H), 3.76 (s, 3H).

^{13}C NMR (75 MHz, $CDCl_3$) δ 176.7, 164.3, 161.5, 134.9, 131.8, 130.4, 114.3, 113.8, 112.1, 93.4, 86.7, 55.5, 55.4.

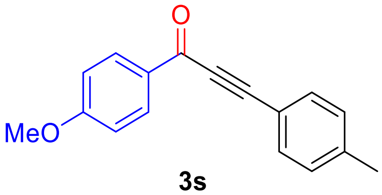

3s

1-(4-methoxyphenyl)-3-(*p*-tolyl) prop-2-yn-1-one (3s)[6]

White solid (40.6 mg, 81% yield), purified by column chromatography (SiO_2, Pentane/EA= 20:1).

^{1}H NMR (300 MHz, $CDCl_3$) δ 8.16 – 8.05 (m, 2H), 7.54 – 7.44 (m, 2H), 7.19 – 7.08 (m, 2H), 6.96 – 6.85 (m, 2H), 3.81 (s, 3H), 2.32 (s, 3H).

^{13}C NMR (75 MHz, $CDCl_3$) δ 176.7, 164.4, 141.3, 133.0, 131.9, 130.3, 129.4, 117.2, 113.8, 92.9, 86.7, 55.5, 21.7.

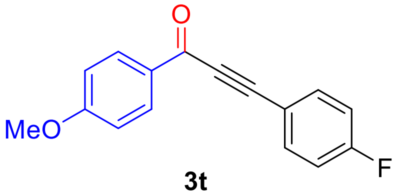

3t

3-(4-fluorophenyl)-1-(4-methoxyphenyl) prop-2-yn-1-one (3t)[7]

White solid (41.4 mg, 81% yield), purified by column chromatography (SiO_2, Pentane/EA= 20:1).

^{1}H NMR (300 MHz, $CDCl_3$) δ 8.15 – 8.03 (m, 2H), 7.65 – 7.52 (m, 2H), 7.10 – 6.96 (m, 2H), 6.96 – 6.85 (m, 2H), 3.81 (s, 3H).

^{13}C NMR (75 MHz, $CDCl_3$) δ 176.5, 164.5, 163.8 (d, J = 253.3 Hz), 135.2 (d, J = 8.8 Hz), 131.9, 130.1, 116.4 (d, J = 3.6 Hz), 116.1 (d, J = 22.4 Hz), 113.9, 91.1, 86.8 (d, J = 1.5 Hz), 55.6.

^{19}F NMR (282 MHz, $CDCl_3$) δ -106.4 – -106.6 (m).

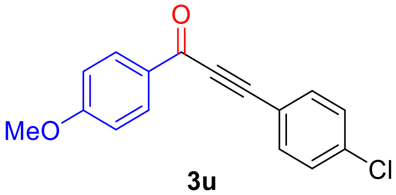

3u

3-(4-chlorophenyl)-1-(4-methoxyphenyl) prop-2-yn-1-one (3u)[8]

White solid (44.4 mg, 82% yield), purified by column chromatography (SiO_2, Pentane/EA= 20:1).

^{1}H NMR (300 MHz, $CDCl_3$) δ 8.14 – 8.03 (m, 2H), 7.56 – 7.45 (m, 2H), 7.36 – 7.24 (m, 2H), 6.96 – 6.85 (m, 2H), 3.81 (s, 3H).

^{13}C NMR (75 MHz, $CDCl_3$) δ 176.4, 164.5, 136.9, 134.1, 131.9, 130.1, 129.1, 118.8, 113.9, 90.8, 87.6, 55.6.

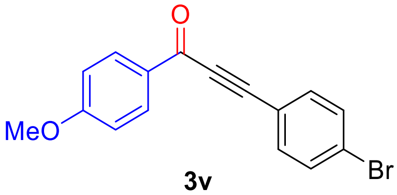

3v

3-(4-bromophenyl)-1-(4-methoxyphenyl) prop-2-yn-1-one (3v)[8]

White solid (50.6 mg, 80% yield), purified by column chromatography (SiO_2, Pentane/EA= 20:1).

^{1}H NMR (300 MHz, $CDCl_3$) δ 8.13 – 8.02 (m, 2H), 7.52 – 7.38 (m, 4H), 6.95 – 6.84 (m, 2H), 3.81 (s, 3H).

^{13}C NMR (75 MHz, $CDCl_3$) δ 176.3, 164.5, 134.2, 132.0, 131.9, 130.1, 125.3, 119.2, 113.9, 90.8, 87.7, 55.6.

HRMS (ESI-TOF): m/z calcd. for $C_{16}H_{12}BrO_2$ [M+H $^+$] 315.0015, found 315.0020.

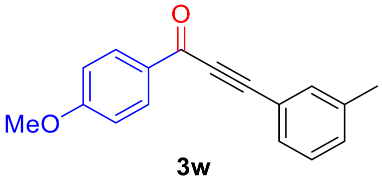

3w

1-(4-methoxyphenyl)-3-(*m*-tolyl) prop-2-yn-1-one (3w)[9]

White solid (31.6 mg, 63% yield), purified by column chromatography (SiO_2, Pentane/EA= 20:1).

^{1}H NMR (300 MHz, $CDCl_3$) δ 8.16 – 8.06 (m, 2H), 7.46 – 7.35 (m, 2H), 7.28 – 7.14 (m, 2H), 6.96 – 6.85 (m, 2H), 3.82 (s, 3H), 2.30 (s, 3H).

^{13}C NMR (75 MHz, $CDCl_3$) δ 176.7, 164.4, 138.4, 133.4, 131.9, 131.5, 130.3, 130.1, 128.5, 120.1, 113.8, 92.6, 86.7, 55.6, 21.2.

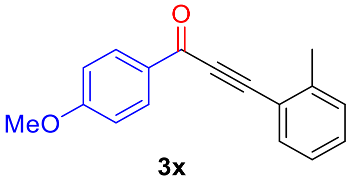

3x

1-(4-methoxyphenyl)-3-(*o*-tolyl) prop-2-yn-1-one (3x)[10]

White solid (41.3 mg, 82% yield), purified by column chromatography (SiO_2, Pentane/EA= 20:1).

^{1}H NMR (300 MHz, $CDCl_3$) δ 8.16 – 8.05 (m, 2H), 7.46 – 7.33 (m, 2H), 7.30 – 7.14 (m, 2H), 6.96 – 6.83 (m, 2H), 3.81 (s, 3H), 2.30 (s, 3H).

^{13}C NMR (75 MHz, $CDCl_3$) δ 176.7, 164.4, 138.4, 133.4, 131.9, 131.5, 130.3, 130.1, 128.5, 120.1, 113.8, 92.7, 86.7, 55.5, 21.1.

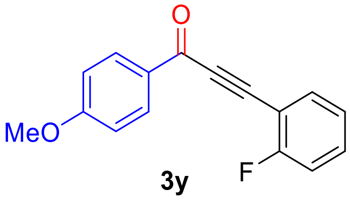

3y

3-(2-fluorophenyl)-1-(4-methoxyphenyl) prop-2-yn-1-one (3y)

Yellow oil (42.0 mg, 83% yield), purified by column chromatography (SiO_2, Pentane/EA= 20:1).

^{1}H NMR (300 MHz, $CDCl_3$) δ 8.20 – 8.08 (m, 2H), 7.62 – 7.51 (m, 1H), 7.48 – 7.32 (m, 1H), 7.16 – 7.04 (m, 2H), 6.97 – 6.85 (m, 2H), 3.82 (s, 3H).

^{13}C NMR (75 MHz, $CDCl_3$) δ 176.3, 164.6, 163.7 (d, J = 254.9 Hz), 134.7, 132.6 (d, J = 8.3 Hz), 132.1, 130.1, 124.4 (d, J = 3.8 Hz), 115.8 (d, J = 20.5 Hz), 113.9, 109.2 (d, J = 15.3 Hz), 91.4 (d, J = 3.3 Hz), 85.4, 55.6.

^{19}F NMR (282 MHz, $CDCl_3$) δ -107.0 – -107.3 (m).

HRMS (ESI-TOF): m/z calcd. for $C_{16}H_{12}FO_2$ [M+H $^+$] 255.0816, found 255.0821.

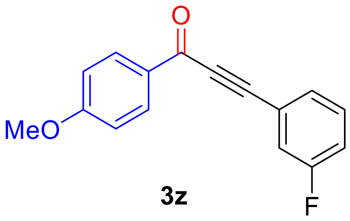

3z

3-(3-fluorophenyl)-1-(4-methoxyphenyl) prop-2-yn-1-one (3z)[11]

White solid (40. mg, 79% yield), purified by column chromatography (SiO_2, Pentane/EA= 20:1).

^{1}H NMR (300 MHz, $CDCl_3$) δ 8.15 – 8.03 (m, 2H), 7.43 – 7.22 (m, 3H), 7.16 – 7.03 (m, 1H), 6.97 – 6.85 (m, 2H), 3.82 (s, 3H).

^{13}C NMR (75 MHz, $CDCl_3$) δ 176.3, 164.6, 162.2 (d, J = 248.0 Hz), 132.0, 130.3 (d, J = 8.4 Hz), 130.0, 128.8 (d, J = 3.2 Hz), 122.1 (d, J = 9.4 Hz), 119.5 (d, J = 23.2 Hz), 118.0 (d, J = 21.2 Hz), 113.9, 90.3 (d, J = 3.4 Hz), 87.2, 55.6.

^{19}F NMR (282 MHz, $CDCl_3$) δ -111.7 (td, J = 8.8, 5.6 Hz).

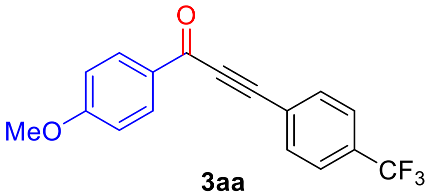

3aa

1-(4-methoxyphenyl)-3-(4-(trifluoromethyl) phenyl) prop-2-yn-1-one (3aa)[12]

White solid (35.5 mg, 58% yield), purified by column chromatography (SiO_2, Pentane/EA= 20:1).

^{1}H NMR (300 MHz, $CDCl_3$) δ 8.16 – 8.04 (m, 2H), 7.75 – 7.54 (m, 4H), 6.97 – 6.86 (m, 2H), 3.83 (s, 3H).

^{13}C NMR (75 MHz, $CDCl_3$) δ 176.2, 164.8, 133.1, 132.1, 132.1 (q, J = 33.0 Hz), 130.0, 125.6 (q, J = 3.8 Hz), 123.8, 114.0, 89.7, 88.2, 55.6.

^{19}F NMR (282 MHz, $CDCl_3$) δ -63.1.

HRMS (ESI-TOF): m/z calcd. for $C_{17}H_{12}F_3O_2$ [M+H $^+$] 305.0784, found 305.0787.

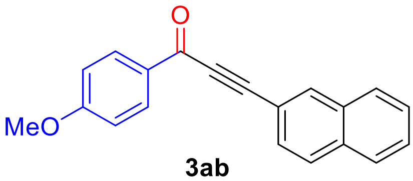

3ab

1-(4-methoxyphenyl)-3-(naphthalen-2-yl) prop-2-yn-1-one (3ab)

Yellow oil (44.4 mg, 78% yield), purified by column chromatography (SiO_2, Pentane/EA= 20:1).

^{1}H NMR (300 MHz, $CDCl_3$) δ 8.19 – 8.07 (m, 3H), 7.81 – 7.69 (m, 3H), 7.55 (dd, J = 8.6, 1.6 Hz, 1H), 7.49 – 7.40 (m, 1H), 6.95 – 6.84 (m, 2H), 3.79 (s, 3H).

^{13}C NMR (75 MHz, $CDCl_3$) δ 176.6, 164.4, 134.0, 133.8, 132.6, 131.9, 130.3, 128.4, 128.3, 128.1, 127.9, 127.8, 126.9, 117.4, 113.8, 92.8, 87.1, 55.5.

HRMS (ESI-TOF): m/z calcd. for $C_{20}H_{15}O_2$ [M+H $^+$] 287.1067, found 287.1071.

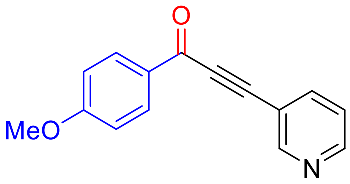

3ac

1-(4-methoxyphenyl)-3-(pyridin-3-yl) prop-2-yn-1-one (3ac)[13]

White solid (41.2 mg, 87% yield), purified by column chromatography (SiO_2, Pentane/EA= 10:1).

^{1}H NMR (300 MHz, $CDCl_3$) δ 8.81 (dd, J = 2.2, 0.9 Hz, 1H), 8.60 (dd, J = 4.9, 1.7 Hz, 1H), 8.16 – 8.04 (m, 2H), 7.92 – 7.82 (m, 1H), 7.36 – 7.24 (m, 1H), 6.97 – 6.86 (m, 2H), 3.83 (s, 3H).

^{13}C NMR (75 MHz, $CDCl_3$) δ 176.0, 164.7, 153.1, 150.5, 139.7, 132.0, 129.9, 123.2, 117.7, 114.0, 89.4, 88.1, 55.6.

HRMS (ESI-TOF): m/z calcd. for $C_{15}H_{12}NO_2$ [M+H $^+$] 238.0863, found 238.0869.

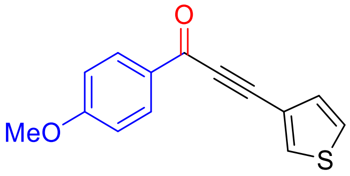

3ad

1-(4-methoxyphenyl)-3-(thiophen-3-yl) prop-2-yn-1-one (3ad)[11]

White solid (38.6 mg, 80% yield), purified by column chromatography (SiO_2, Pentane/EA= 10:1).

^{1}H NMR (300 MHz, $CDCl_3$) δ 8.15 – 8.02 (m, 2H), 7.73 (dd, J = 2.9, 1.2 Hz, 1H), 7.28 (dd, J = 5.0, 3.0 Hz, 1H), 7.22 (dd, J = 5.0, 1.2 Hz, 1H), 6.95 – 6.84 (m, 2H), 3.81 (s, 3H).

^{13}C NMR (75 MHz, $CDCl_3$) δ 176.6, 164.4, 133.4, 131.9, 130.2, 126.1, 119.5, 113.8, 87.6, 87.1, 55.5.

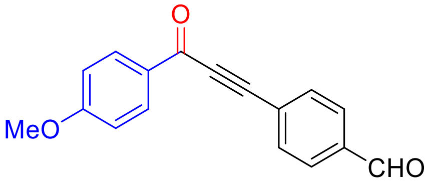

3ae

4-(3-(4-methoxyphenyl)-3-oxoprop-1-yn-1-yl) benzaldehyde (3ae)[8]

White solid (30 mg, 57% yield), purified by column chromatography (SiO_2, Pentane/EA= 10:1).

^{1}H NMR (300 MHz, $CDCl_3$) δ 9.99 (s, 1H), 8.15 – 8.04 (m, 2H), 7.90 – 7.79 (m, 2H), 7.79 – 7.68 (m, 2H), 6.97 – 6.87 (m, 2H), 3.83 (s, 3H).

^{13}C NMR (75 MHz, $CDCl_3$) δ 191.1, 176.1, 164.7, 136.9, 133.3, 132.0, 129.9, 129.6, 126.3, 114.0, 90.0, 89.2, 55.6.

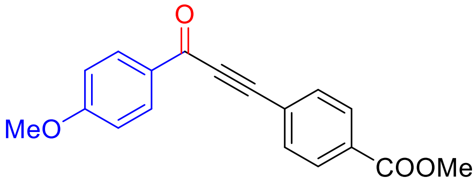

3af

methyl 4-(3-(4-methoxyphenyl)-3-oxoprop-1-yn-1-yl) benzoate(3af)

Yellow oil (45.1 mg, 77% yield), purified by column chromatography (SiO_2, Pentane/EA= 10:1).

^{1}H NMR (300 MHz, $CDCl_3$) δ 8.17 – 8.05 (m, 2H), 8.03 – 7.97 (m, 2H), 7.70 – 7.60 (m, 2H), 6.97 – 6.86 (m, 2H), 3.87 (s, 3H), 3.83 (s, 3H).

^{13}C NMR (75 MHz, $CDCl_3$) δ 176.3, 166.1, 164.7, 132.7, 132.0, 131.5, 130.0, 129.6, 124.8, 113.9, 90.5, 88.6, 55.6, 52.4.

HRMS (ESI-TOF): m/z calcd. for $C_{18}H_{15}O_4$ [M+H $^{+}$] 295.0965, found 295.0973.

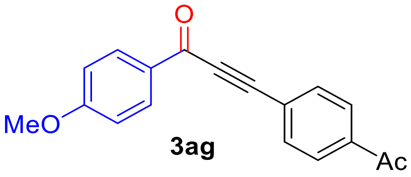

3ag

3-(4-acetylphenyl)-1-(4-methoxyphenyl) prop-2-yn-1-one (3ag)

Yellow oil (28.9 mg, 52% yield), purified by column chromatography (SiO_2, Pentane/EA= 10:1).

^{1}H NMR (300 MHz, $CDCl_3$) δ 8.17 – 8.05 (m, 2H), 7.97 – 7.86 (m, 2H), 7.73 – 7.62 (m, 2H), 6.97 – 6.87 (m, 2H), 3.83 (s, 3H), 2.56 (s, 3H).

^{13}C NMR (75 MHz, $CDCl_3$) δ 197.1, 176.3, 164.7, 137.9, 132.9, 132.0, 130.0, 128.3, 124.9, 114.0, 90.4, 88.9, 55.6, 26.7.

HRMS (ESI-TOF): m/z calcd. for $C_{18}H_{15}O_3$ [M+H $^+$] 279.1016, found 279.1019.

5. Control experiments

5.1 Radical inhibition experiments

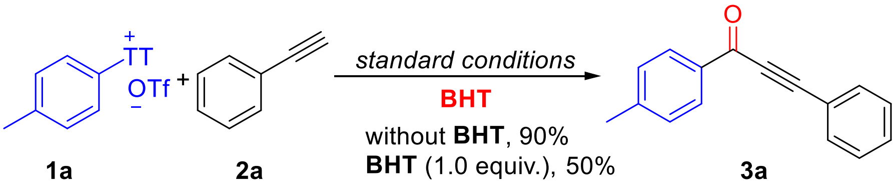

1a 2a 3a

without **BHT**, 90%
BHT (1.0 equiv.), 50%
BHT (2.0 equiv.), 37%
BHT (3.0 equiv.), 34%

Scheme S1. Radical inhibition experiments

A 4 mL snap vial equipped with a magnetic stir bar was charged with $PdBr_2$ (0.004 mmol, 2 mol %), PCy_3 (0.012 mmol, 6 mol%), K_3PO4 (0.4 mmol, 2.0 equiv.), aryl sulfonium salts (0.3 mmol, 1.5 equiv.) and closed with a rubber-based septum. Different amounts of 2,6-di-tert-butyl-4-methylphenol (BHT) (1 – 3 equiv.) were added sequentially to the corresponding vials. The vial was evacuated and backfilled with argon. Degassed DMF (2.0 mL) and aryl acetylenes (0.2 mmol, 1.0 equiv.) were added via syringe. The vial was then connected to atmosphere with a cannula and transferred into a 300 mL Parr 4560 series autoclave, under argon counterflow. The closed autoclave was flushed three times with nitrogen (~ 5 bar), three times with CO (~ 5 bar), and 1 bar of carbon monoxide (measured by pressure meter) was charged. The autoclave was then placed into an aluminum block on a magnetic stirrer. The reaction mixture was stirred (500 rpm) at 80 °C (aluminum block) 15 h. And a proper amount of solvent was taken for GC analysis. The result is shown above.

As BHT was added to the reaction system, the reaction was gradually inhibited.

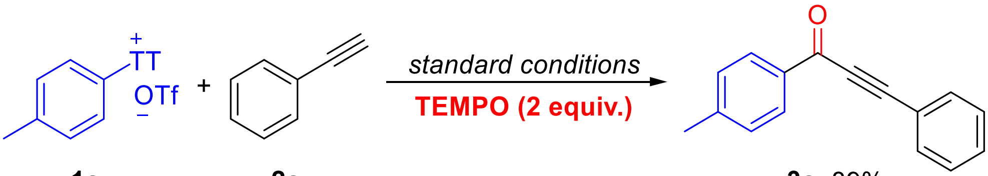

1a 2a **3a**, 89%

Scheme S4. Radical capture experiment

A 4 mL snap vial equipped with a magnetic stir bar was charged $PdBr_2$ (0.004 mmol, 2 mol %), PCy_3 (0.012 mmol, 6 mol%), K_3PO4 (0.4 mmol, 2.0 equiv.), aryl sulfonium salts (0.3 mmol, 1.5 equiv.), 2,2,6,6-tetramethylpiperidine-1-oxyl (TEMPO) (2 equiv.) and closed with a rubber-based septum. The vial was evacuated and backfilled with argon. Degassed DMF (2.0 mL) and substrate aryl acetylenes (0.2 mmol, 1.0 equiv.) were added via syringe. The vial was then connected to atmosphere with a cannula and transferred into a 300 mL Parr 4560 series autoclave, under argon counterflow. The closed autoclave was flushed three times with nitrogen (~ 5 bar), three times with CO (~ 5 bar), and 1 bar of carbon monoxide (measured by pressure meter) was charged. The autoclave was then placed into an aluminum block on a magnetic stirrer. The reaction mixture was stirred (500 rpm) at 80 °C (aluminum block) 15 h. And a proper amount of solvent was taken for GC analysis. The result is shown above.

5.3 Radical capture experiment

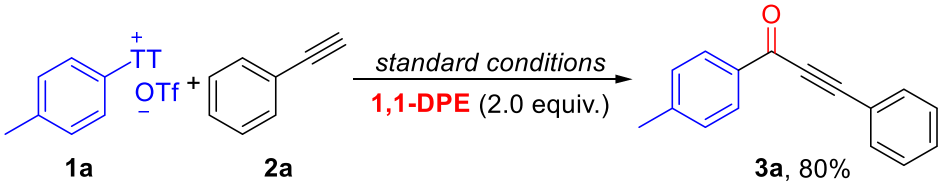

A 4 mL snap vial equipped with a magnetic stir bar was charged $PdBr_2$ (0.004 mmol, 2 mol %), PCy_3 (0.012 mmol, 6 mol%), K_3PO4 (0.4 mmol, 2.0 equiv.), aryl sulfonium salts (0.3 mmol, 1.5 equiv.), 1,1-DPE (2 equiv.) and closed with a rubber-based septum. The vial was evacuated and backfilled with argon. Degassed DMF (2.0 mL) and substrate aryl acetylenes (0.2 mmol, 1.0 equiv.) were added via syringe. The vial was then connected to atmosphere with a cannula and transferred into a 300 mL Parr 4560 series autoclave, under argon counterflow. The closed autoclave was flushed three times with nitrogen (~ 5 bar), three times with CO (~ 5 bar), and 1 bar of carbon monoxide (measured by pressure meter) was charged. The autoclave was then placed into an aluminum block on a magnetic stirrer. The reaction mixture was stirred (500 rpm) at 80 ℃ (aluminum block) 15 h. And a proper amount of solvent was taken for GC analysis. The result is shown above.

6. Reference

[1] (a) Gu, X.-W.; Zhao, Y.-H.; Wu, X.-F. [3 + 2] Cycloaddition of azides with arynes formed via C–H deprotonation of aryl sulfonium salts. *Green Chem.* **2023**, *25*, 6282-6286. (b) Tian, Z.-Y.; Lin, Z.-H.; Zhang, C.-P. Pd/Cu-Catalyzed C–H/C–H Cross Coupling of (Hetero)Arenes with Azoles through Arylsulfonium Intermediates. *Org. Lett.* **2021**, *23*, 4400-4405.

[2] Sun, W.; Wang, Y.; Wu, X.; Yao, X. Palladium-, ligand-, and solvent-free synthesis of ynones by the coupling of acyl chlorides and terminal alkynes in the presence of a reusable copper nanoparticle catalyst. *Green Chem.* **2013**, *15*, 2356-2360.

[3] Bai, C.; Jian, S.; Yao, X.; Li, Y. Carbonylative Sonogashira coupling of terminal alkynes with aryl iodides under atmospheric pressure of CO using Pd(ii)@MOF as the catalyst. *Catal. Sci. Technol.* **2014**, *4*, 3261-3267.

[4] Cen, K.; Wei, J.; Liu, Y.; Tan, Z.; Wang, X.; Wang, D.; He, W.-M.; Cai, J. Electrochemical Selenylation of Alkynyl Aryl Ketones: Efficient Synthesis of 3-Selenylated Chromones under Catalyst- and Chemical-Oxidant-Free Conditions. *Eur. J. Org. Chem.* **2023**, *26*, e202300968.

[5] Li, X.; Li, Y.; Yang, J.; Shi, H.; Ai, Z.; Han, C.; He, J.; Du, Y. Synthesis of 3-SCF2H-/3-SCF3-chromones via Interrupted Pummerer Reaction/Intramolecular Cyclization Mediated by Difluoromethyl or Trifluoromethyl Sulfoxide and Tf_2O. *Org. Lett.* **2022**, *24*, 7216-7221.

[6] De La Cruz, L. K.; Bauer, N.; Cachuela, A.; Tam, W. S.; Tripathi, R.; Yang, X.; Binghe Wang, Light-Activated CO Donor as a Universal CO Surrogate for Pd-Catalyzed and Light-Mediated Carbonylation. *Org. Lett.* **2022**, *24*, 4902-4907.

[7] Sarma, M. J.; Sudarshana, K. A.; Pabbaraja, S.; Mehta, G. Diversified Stitching of Ynones with Oxindole-3-oxy acrylates: One-Flask Spiro-annulation Protocol toward Assorted 3H/5H-Spiro[furan-2,3′-indolin]-2′-ones. *J. Org. Chem.* **2023**, *88*, 12131-12140.

[8] Zhang, K.; Yao, Y.; Sun, W.; Wen, R.; Wang, Y.; Sun, H.; Zhang, W.; Zhang, G.; Gao, Z. Triazine-wingtips accelerated NHC-Pd catalysed carbonylative Sonogashira cross-coupling reaction. *Chem. Commun.*, **2021**, *57*, 13020-13023.

[9] Feng, X.; Song, J.; Bao, M. Carbonylative Sonogashira Coupling of Aryl Iodides with Terminal Alkynes Catalyzed by Palladium Nanoparticles. *J. Chin. Chem. Soc.* **2018**, *65*, 337-345.

[10] Shiroodi, R. K.; Soltani, M.; Gevorgyan, V. Gold-Catalyzed 1,3-Transposition of Ynones. *J. Am. Chem. Soc.* **2014**, *136*, 9882-9885.

[11] Karadeniz, E.; Zora, M. Synthesis of 1-Azaspiro[4.5]deca-1,3-dienes from N-Propargylic β-Enaminones in Basic Medium. *Synthesis* **2019**, *51*, 2157-2170.

[12] Breuer, N.; Gruber, I.; Janiak, C.; Müller, T. J. J. Emission solvatochromic, solid-state and aggregation-induced emissive α-pyrones and emission-tuneable 1H-pyridines by Michael addition-cyclocondensation sequences. *Beilstein J. Org. Chem.* **2019**, *15*, 2684-2703

[13] Rao, P. N. P.; Uddin, M. J.; Knaus, E. E. Design, Synthesis, and Structure−Activity Relationship Studies of 3,4,6-Triphenylpyran-2-ones as Selective Cyclooxygenase-2 Inhibitors. *J. Med. Chem.* **2004**, *47*, 3972-3990.

7. NMR spectra of products

240322.317.10.fid — Yanhua Zhao ZYH-216 — Au1H CDCl3 {C:\Bruker\TopSpin3.6.2} 2403 17 - 300 MHz

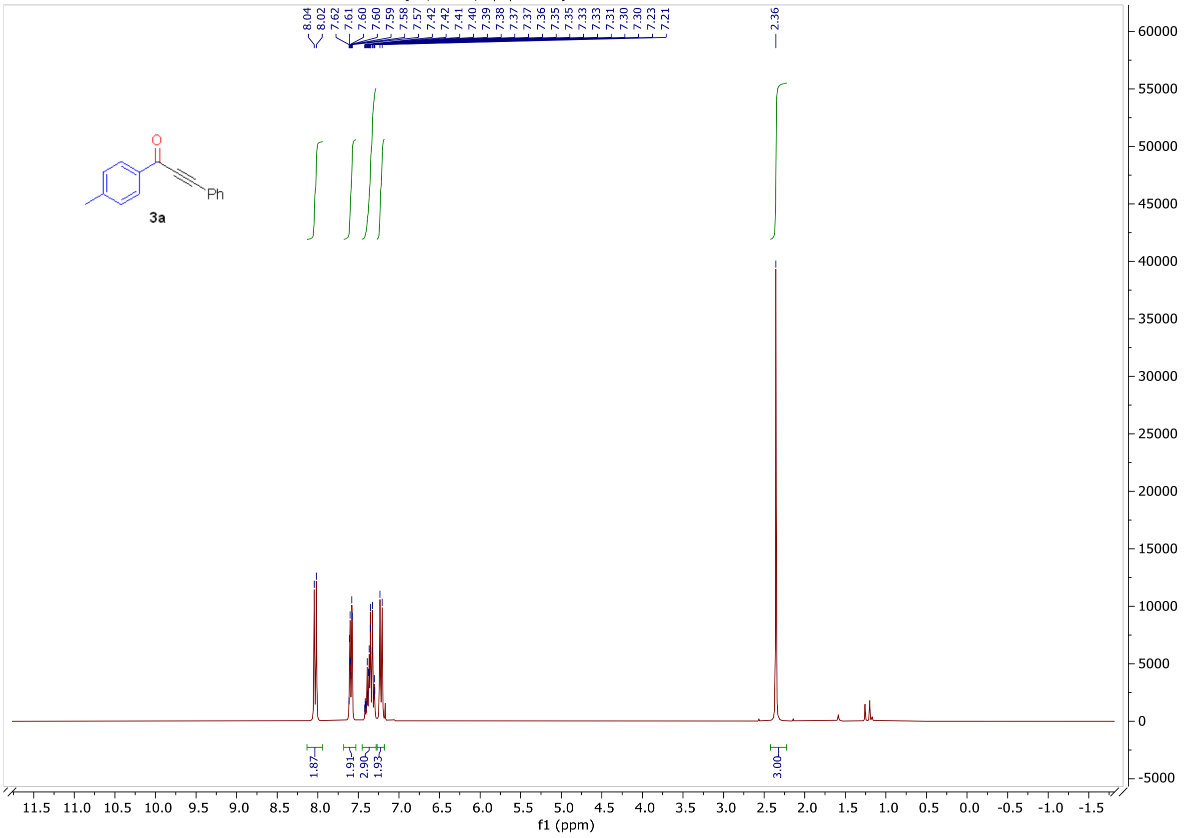

240322.317.11.fid — Yanhua Zhao ZYH-216 — Au13C CDCl3 {C:\Bruker\TopSpin3.6.2} 2403 17 - 75 MHz

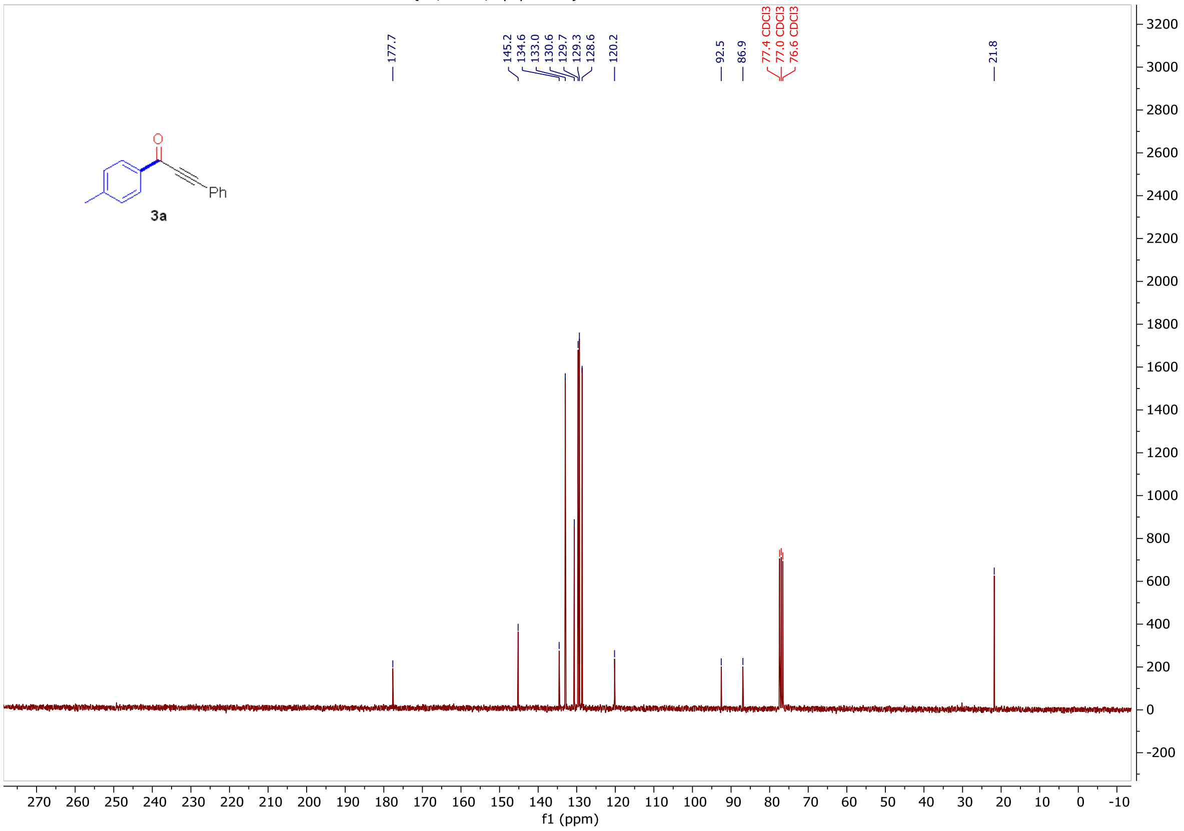

240411.305.10.fid — Yanhua Zhao ZYH-230-1 — Au1H CDCl3 {C:\Bruker\TopSpin3.6.2} 2404 5 - 300 MHz

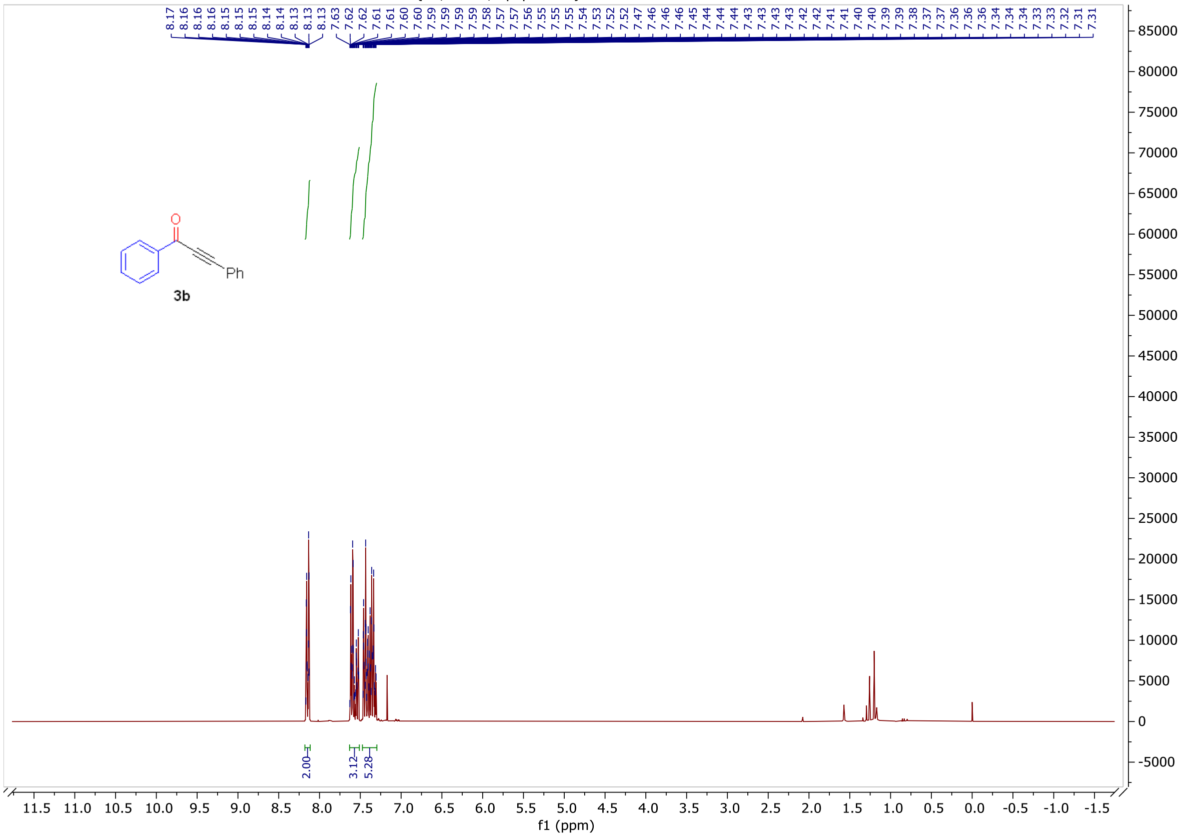

240411.305.11.fid — Yanhua Zhao ZYH-230-1 — Au13C CDCl3 {C:\Bruker\TopSpin3.6.2} 2404 5 - 75 MHz

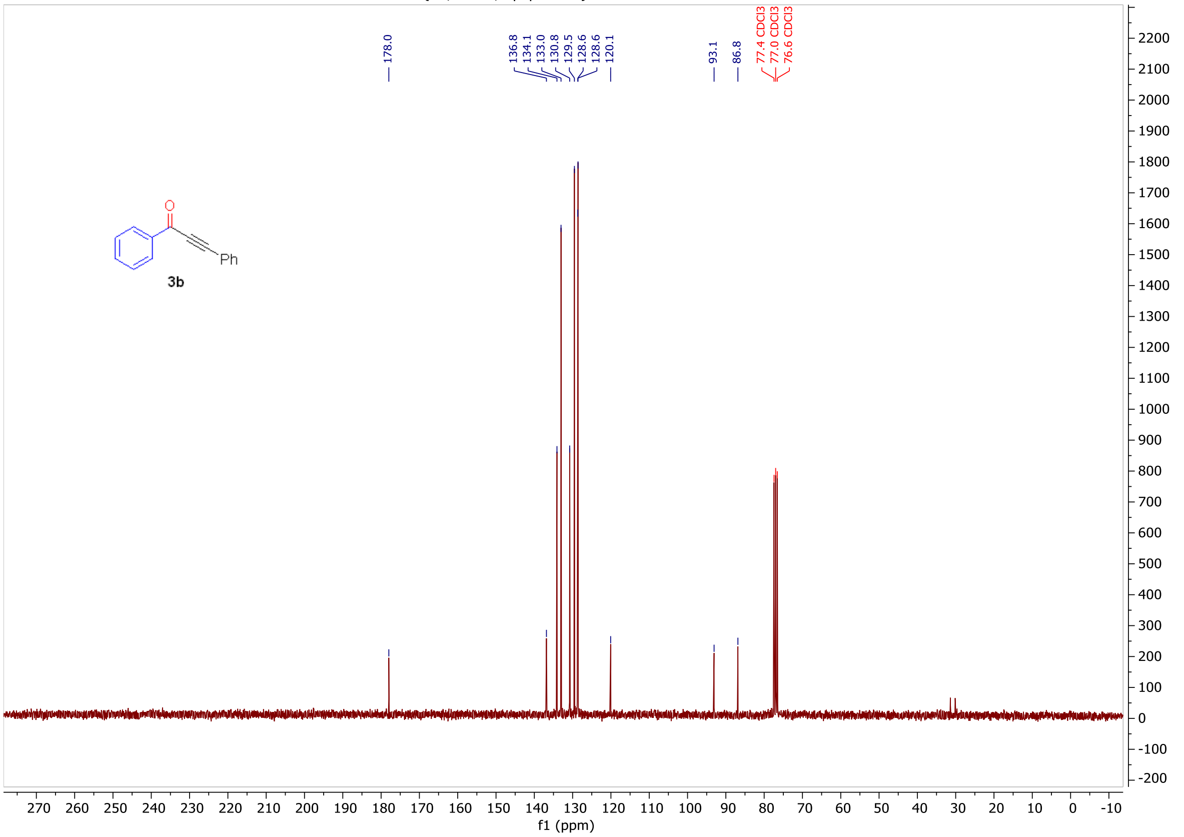

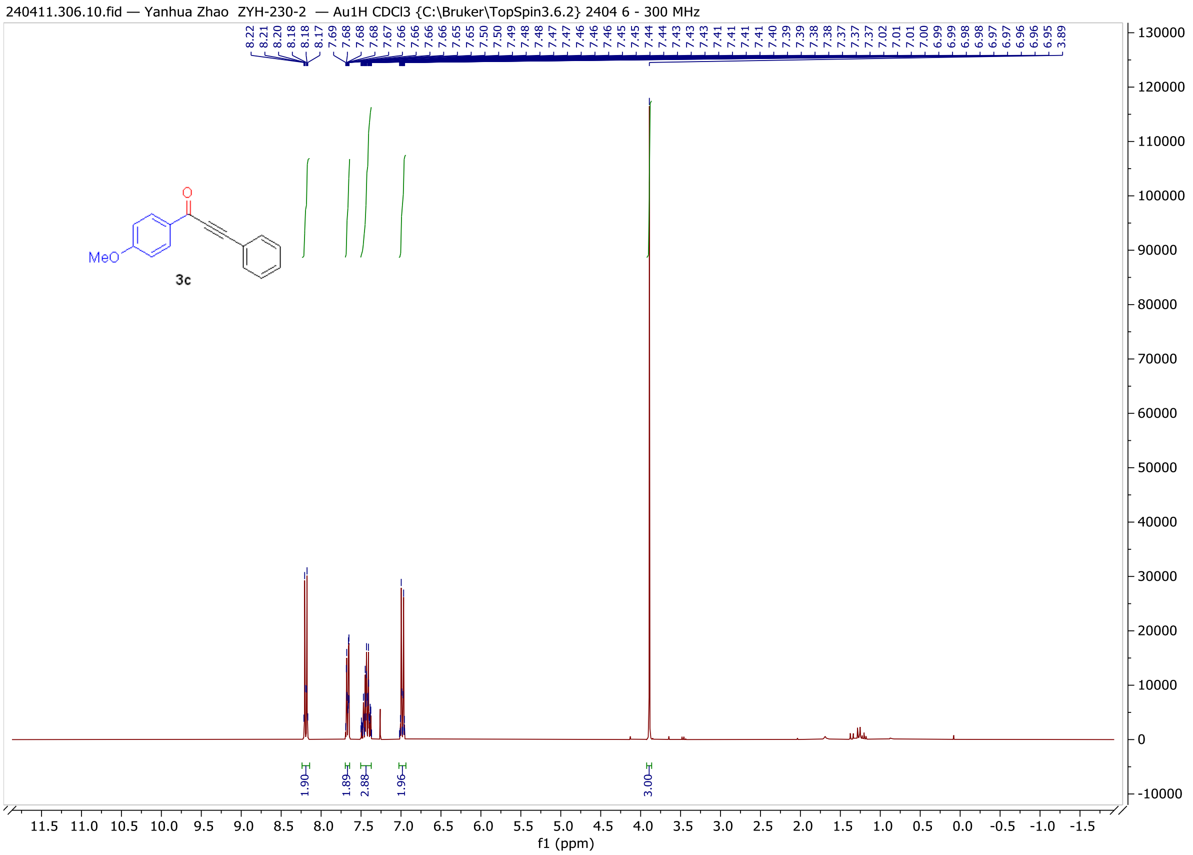
240411.306.10.fid — Yanhua Zhao ZYH-230-2 — Au1H CDCl3 {C:\Bruker\TopSpin3.6.2} 2404 6 - 300 MHz
MeO
O
3c
1.90
1.89
2.88
1.96
3.00
f1 (ppm)

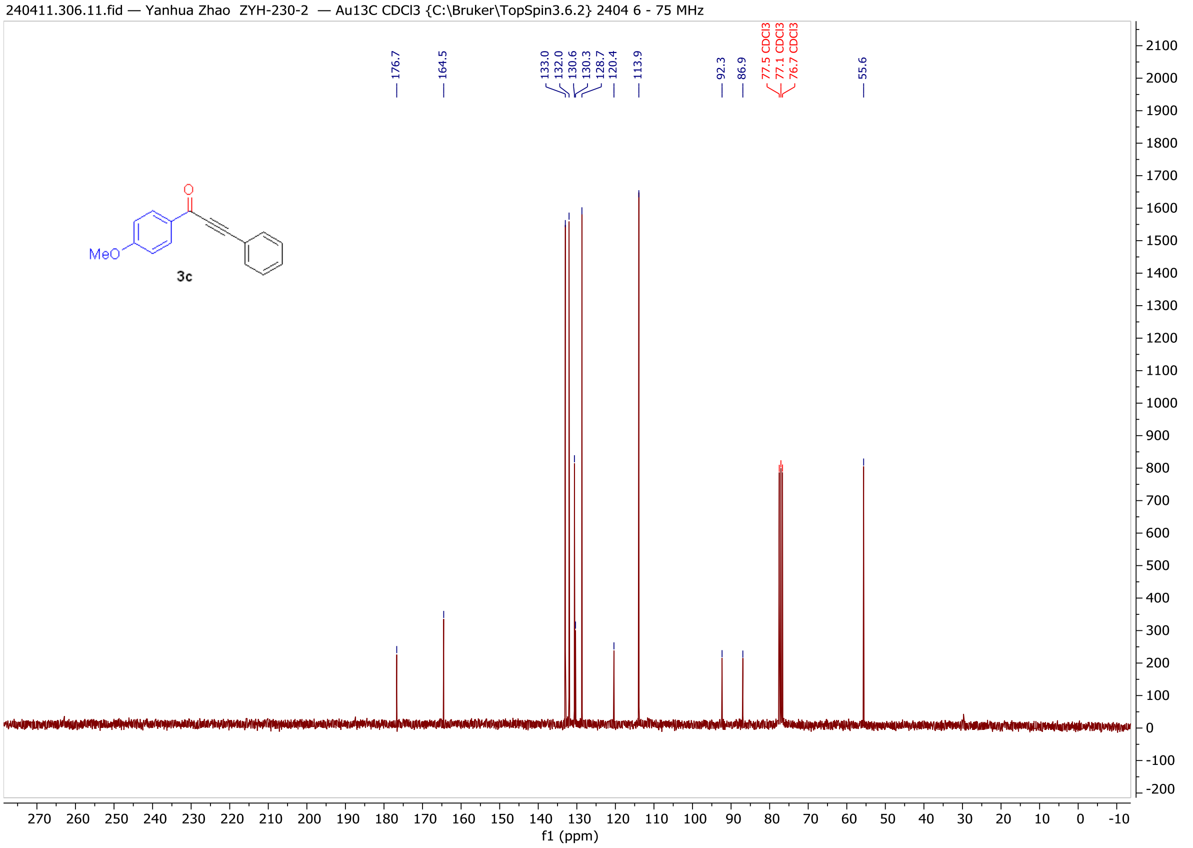
240411.306.11.fid — Yanhua Zhao ZYH-230-2 — Au13C CDCl3 {C:\Bruker\TopSpin3.6.2} 2404 6 - 75 MHz
176.7
164.5
133.0
132.0
130.6
130.3
128.7
120.4
113.9
92.3
86.9
77.5 CDCl3
77.1 CDCl3
76.7 CDCl3
55.6
MeO
O
3c
f1 (ppm)

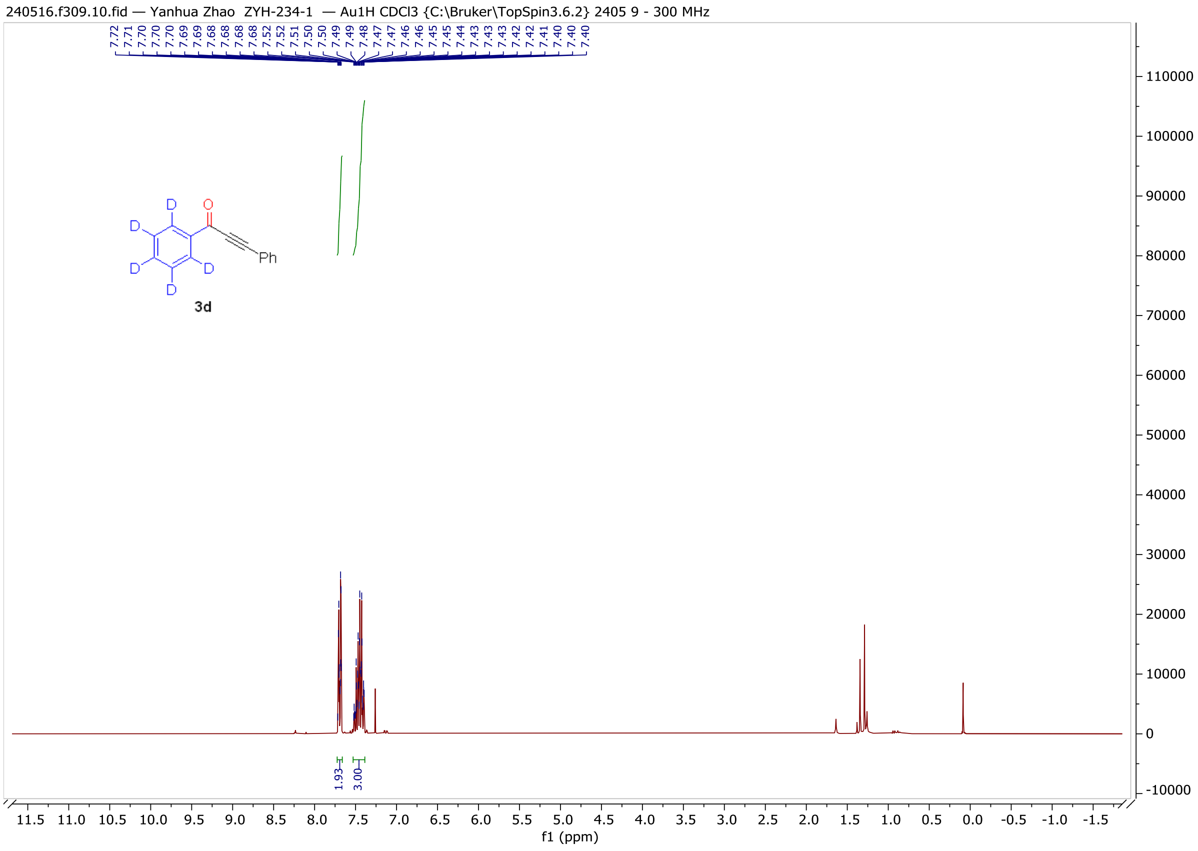
240516.f309.10.fid — Yanhua Zhao ZYH-234-1 — Au1H CDCl3 {C:\Bruker\TopSpin3.6.2} 2405 9 - 300 MHz
7.72 7.71 7.70 7.70 7.69 7.69 7.68 7.68 7.68 7.68 7.52 7.52 7.51 7.50 7.50 7.49 7.49 7.48 7.47 7.47 7.46 7.46 7.45 7.45 7.44 7.43 7.43 7.43 7.42 7.42 7.41 7.40 7.40 7.40
3d
1.93
3.00
f1 (ppm)

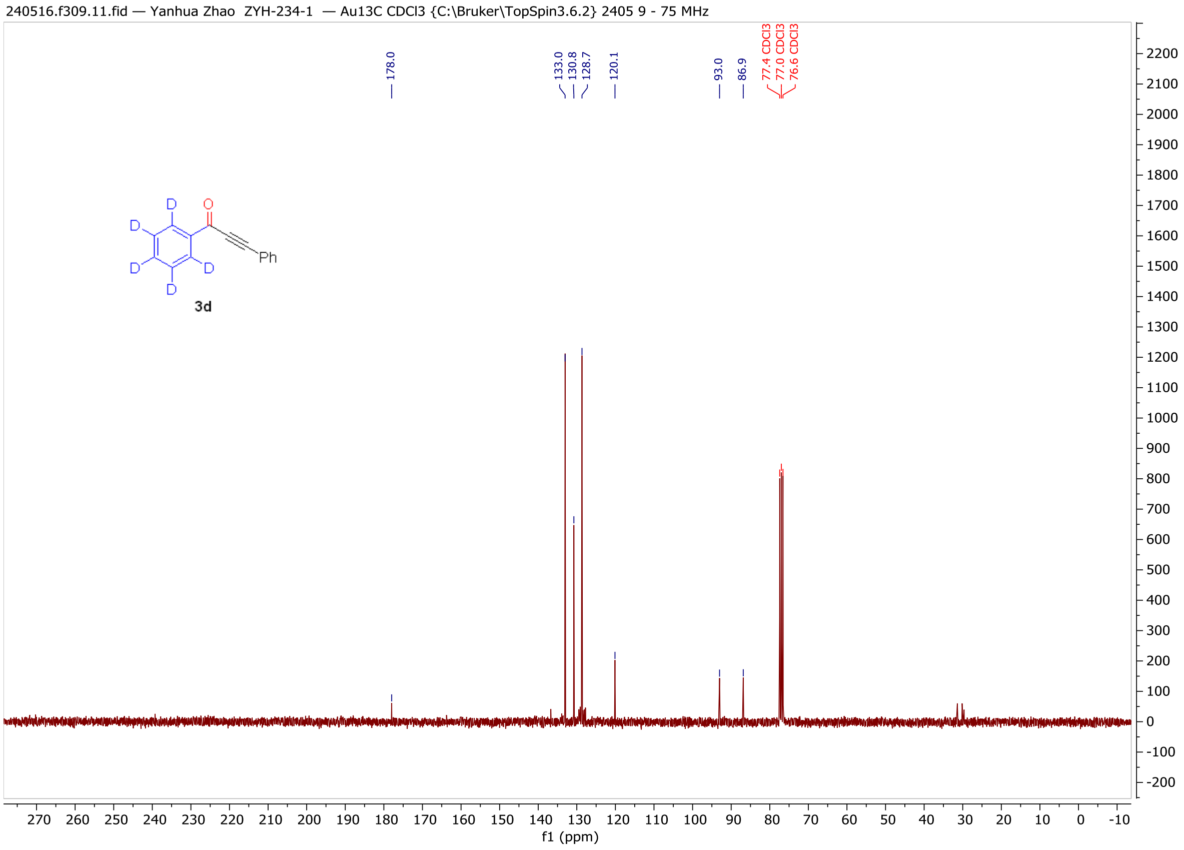
240516.f309.11.fid — Yanhua Zhao ZYH-234-1 — Au13C CDCl3 {C:\Bruker\TopSpin3.6.2} 2405 9 - 75 MHz
178.0
133.0 130.8 128.7
120.1
93.0
86.9
77.4 CDCl3 77.0 CDCl3 76.6 CDCl3
3d
f1 (ppm)

240412.f306.10.fid — Yanhua Zhao ZYH-230-3 — Au1H CDCl3 {C:\Bruker\TopSpin3.6.2} 2404 6 - 300 MHz

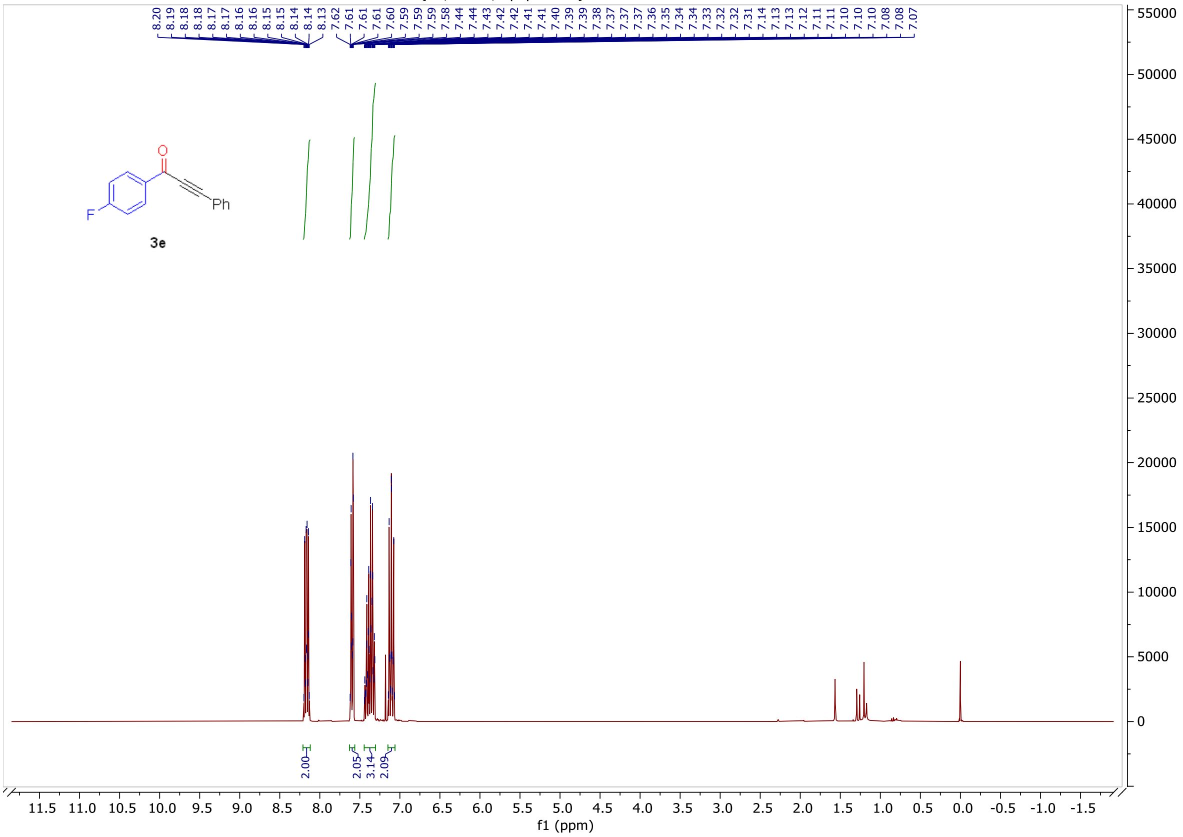

3e

240412.f306.11.fid — Yanhua Zhao ZYH-230-3 — Au13C CDCl3 {C:\Bruker\TopSpin3.6.2} 2404 6 - 75 MHz

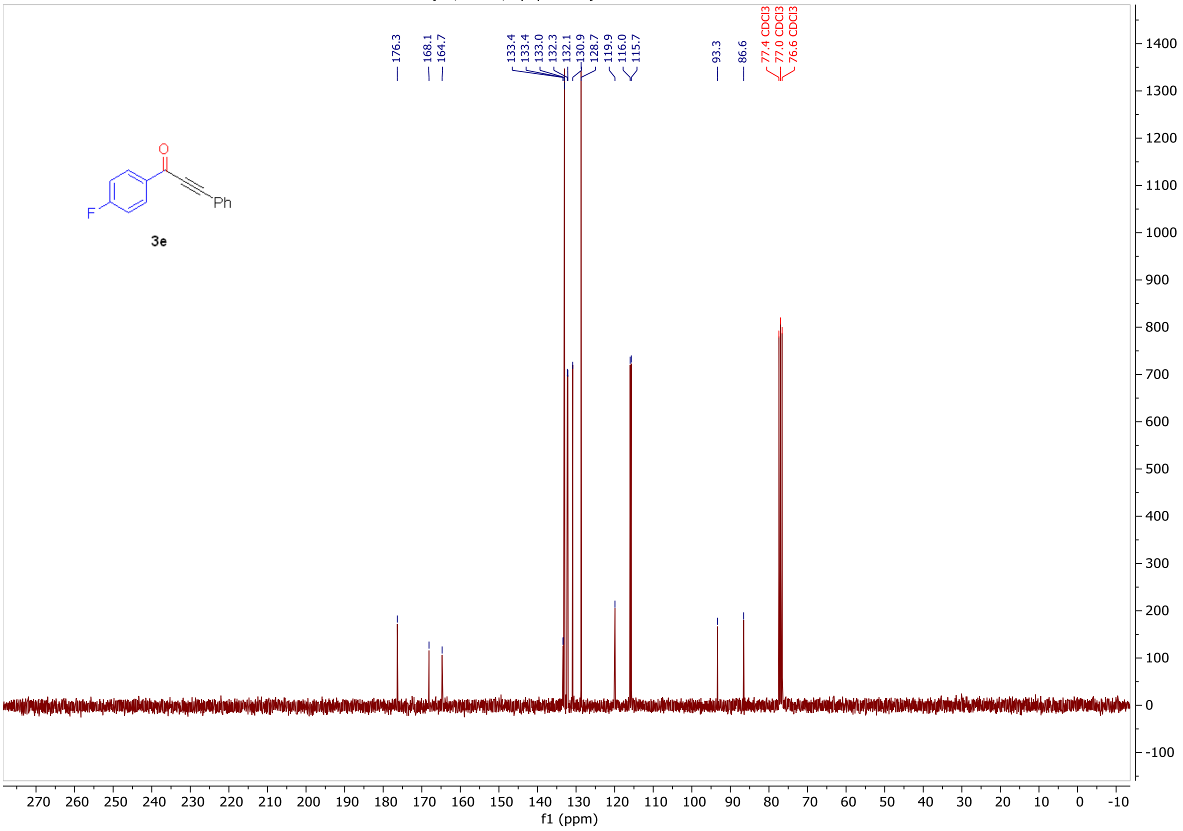

3e

240412.f304.10.fid — Yanhua Zhao ZYH-230-4 — Au1H CDCl3 {C:\Bruker\TopSpin3.6.2} 2404 4 - 300 MHz

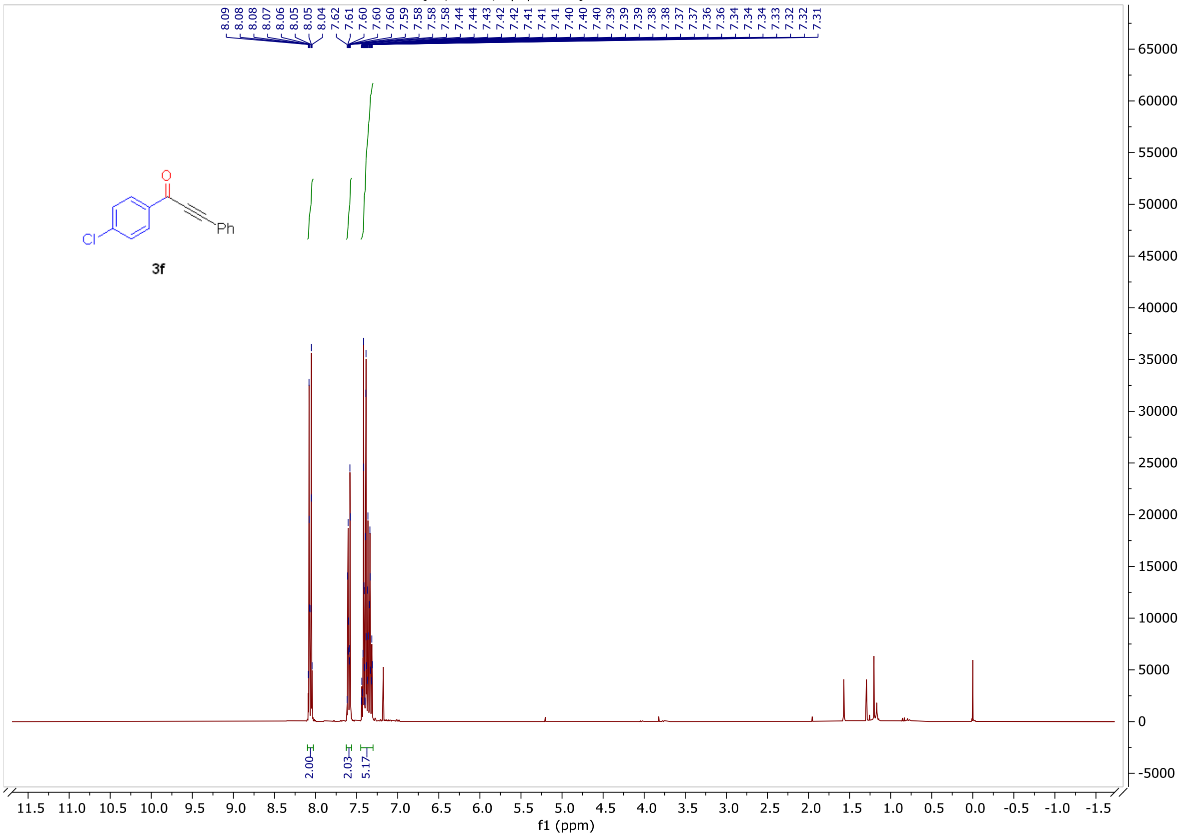

240412.f304.11.fid — Yanhua Zhao ZYH-230-4 — Au13C CDCl3 {C:\Bruker\TopSpin3.6.2} 2404 4 - 75 MHz

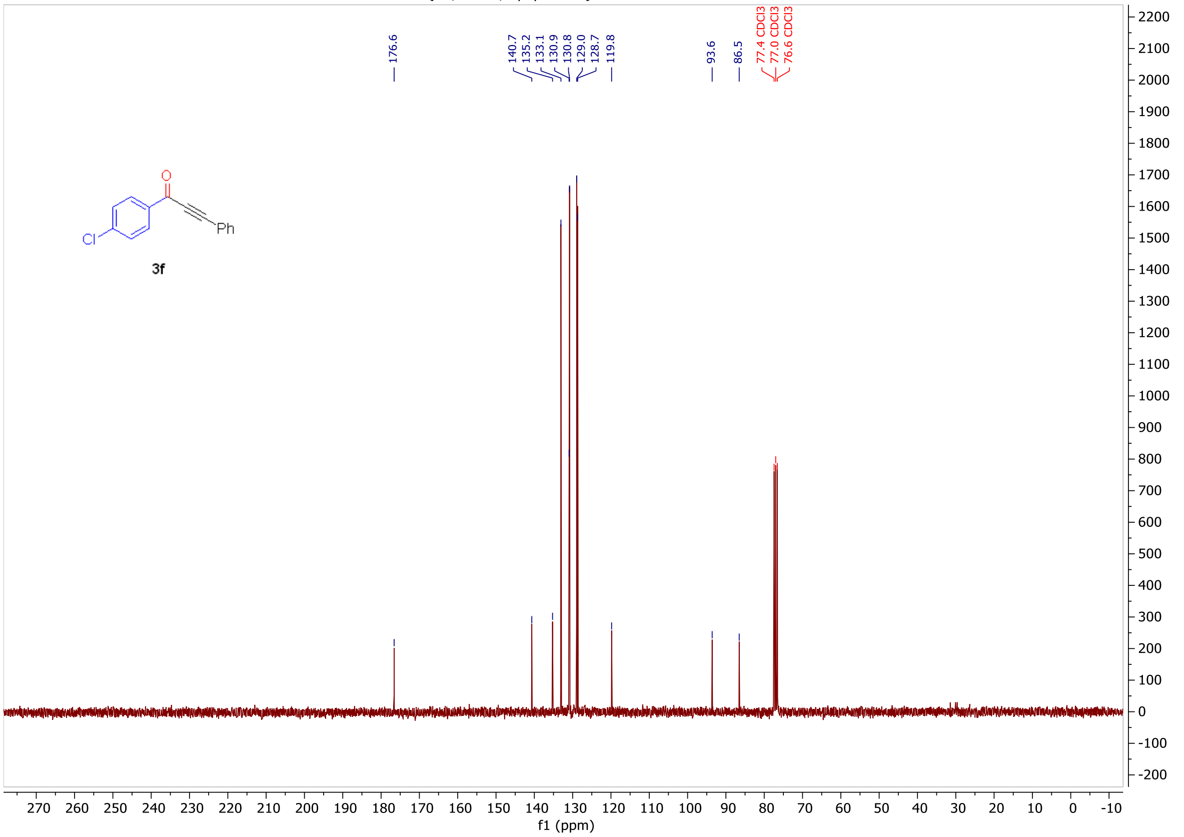

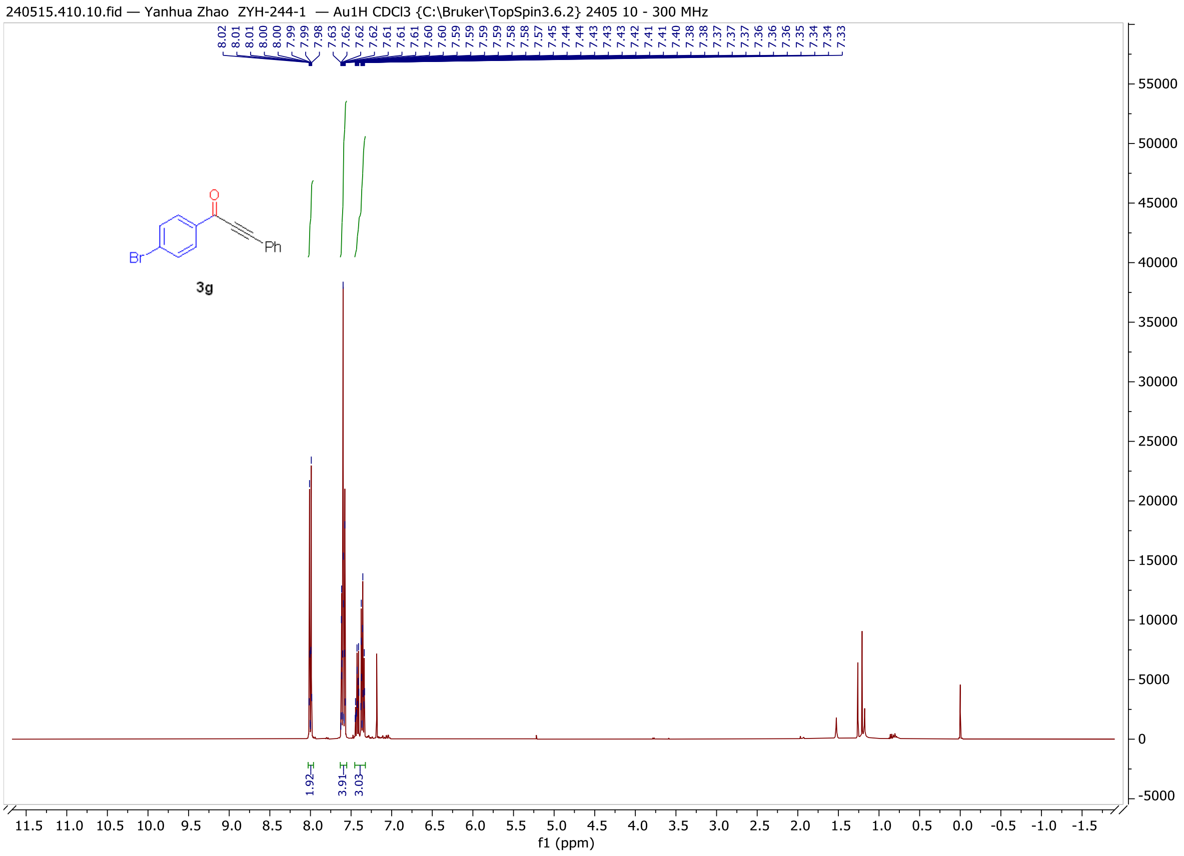
240515.410.10.fid — Yanhua Zhao ZYH-244-1 — Au1H CDCl3 {C:\Bruker\TopSpin3.6.2} 2405 10 - 300 MHz
8.02 8.01 8.01 8.00 8.00 7.99 7.99 7.98 7.63 7.62 7.62 7.62 7.61 7.61 7.61 7.60 7.60 7.59 7.59 7.59 7.59 7.58 7.58 7.57 7.45 7.44 7.44 7.43 7.43 7.43 7.42 7.41 7.41 7.40 7.38 7.38 7.37 7.37 7.37 7.36 7.36 7.36 7.35 7.34 7.34 7.33
O
Br
Ph
3g
1.92
3.91
3.03
f1 (ppm)

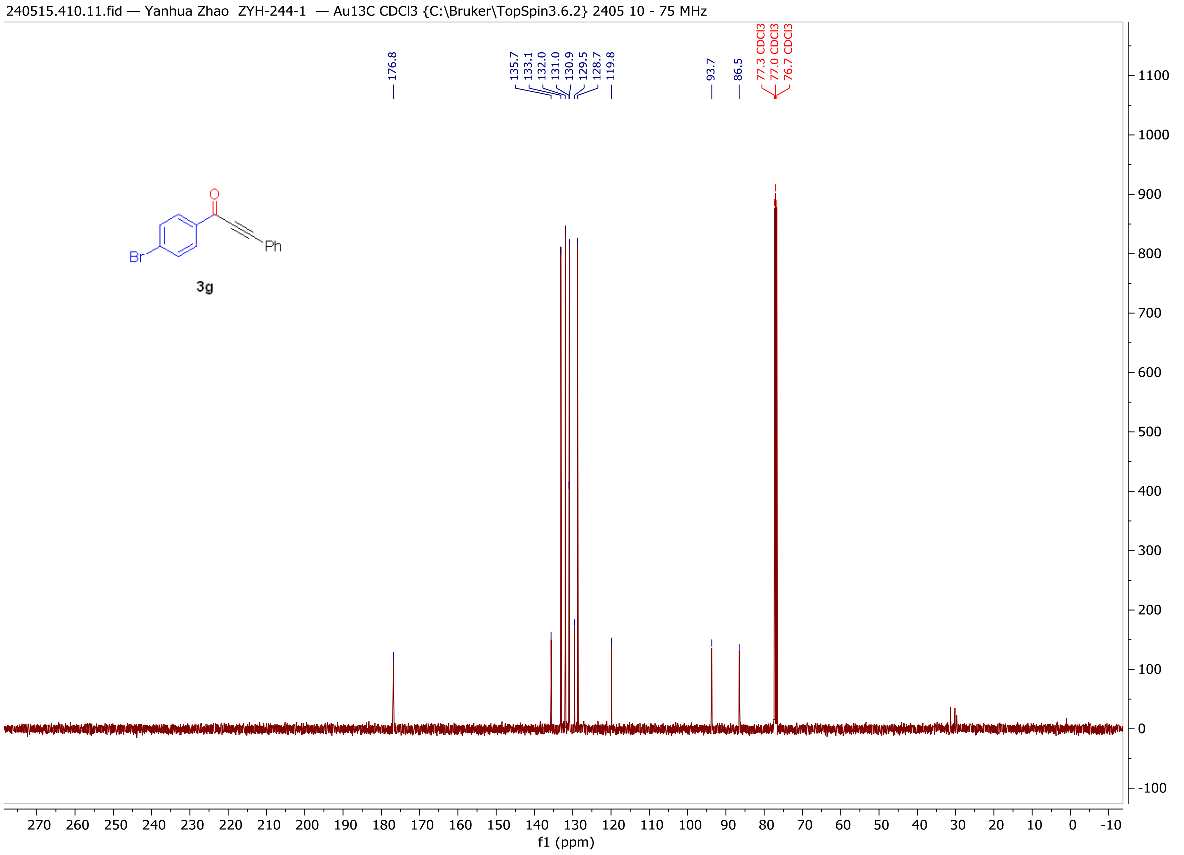
240515.410.11.fid — Yanhua Zhao ZYH-244-1 — Au13C CDCl3 {C:\Bruker\TopSpin3.6.2} 2405 10 - 75 MHz
176.8
135.7 133.1 132.0 131.0 130.9 129.5 128.7 119.8
93.7
86.5
77.3 CDCl3 77.0 CDCl3 76.7 CDCl3
O
Br
Ph
3g
f1 (ppm)

240412.f305.10.fid — Yanhua Zhao ZYH-230-5 — Au1H CDCl3 {C:\Bruker\TopSpin3.6.2} 2404 5 - 300 MHz

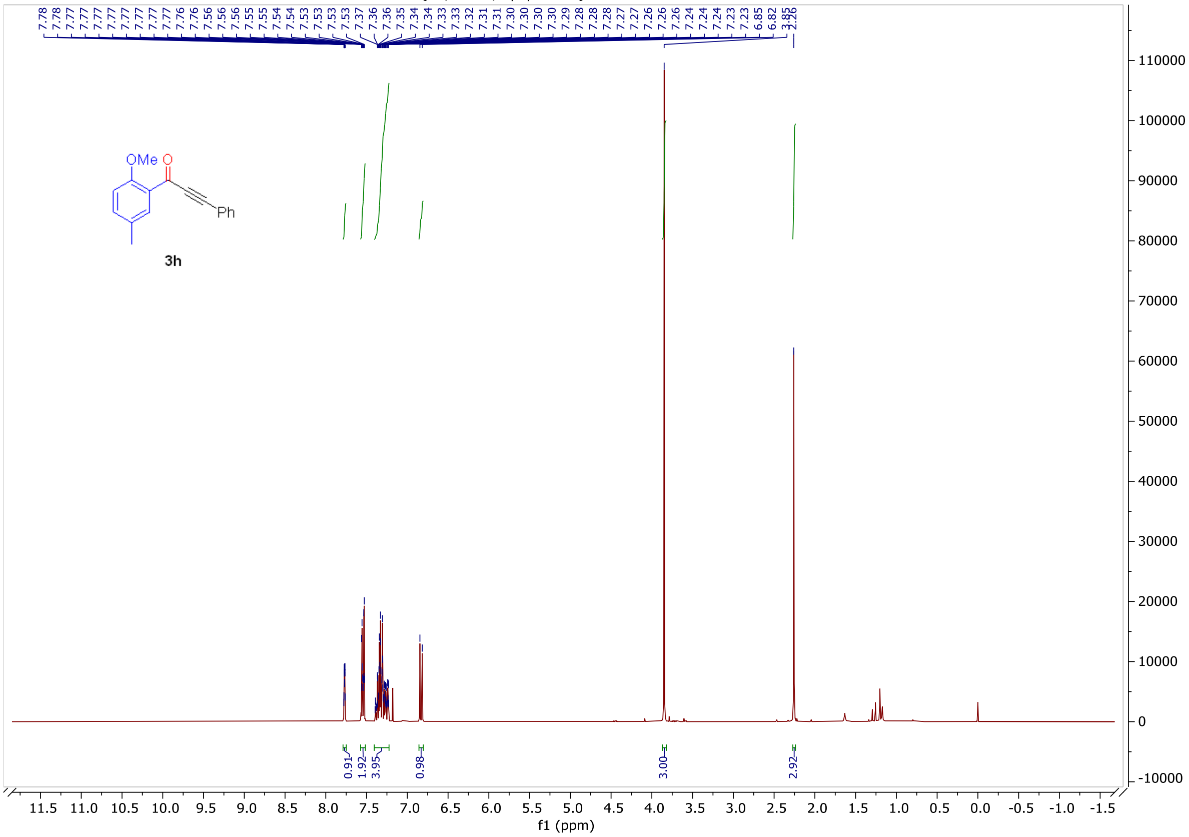

240412.f305.11.fid — Yanhua Zhao ZYH-230-5 — Au13C CDCl3 {C:\Bruker\TopSpin3.6.2} 2404 5 - 75 MHz

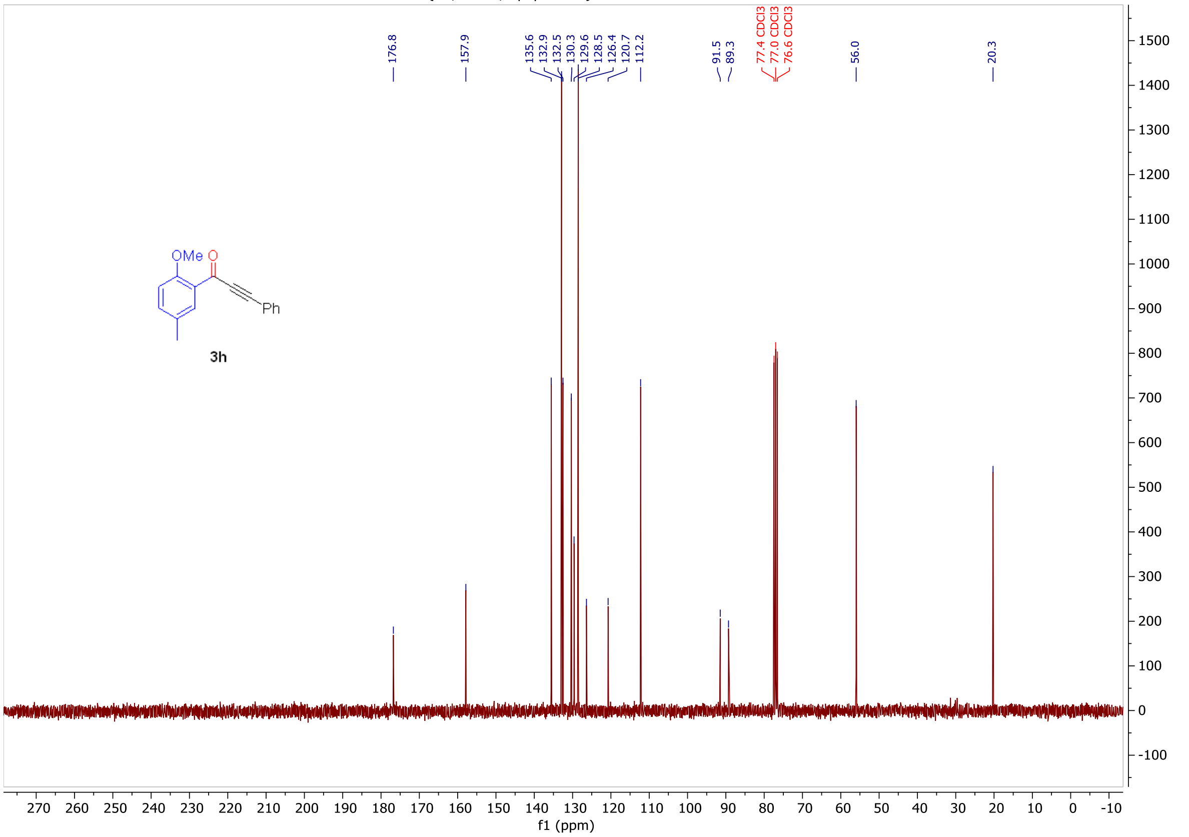

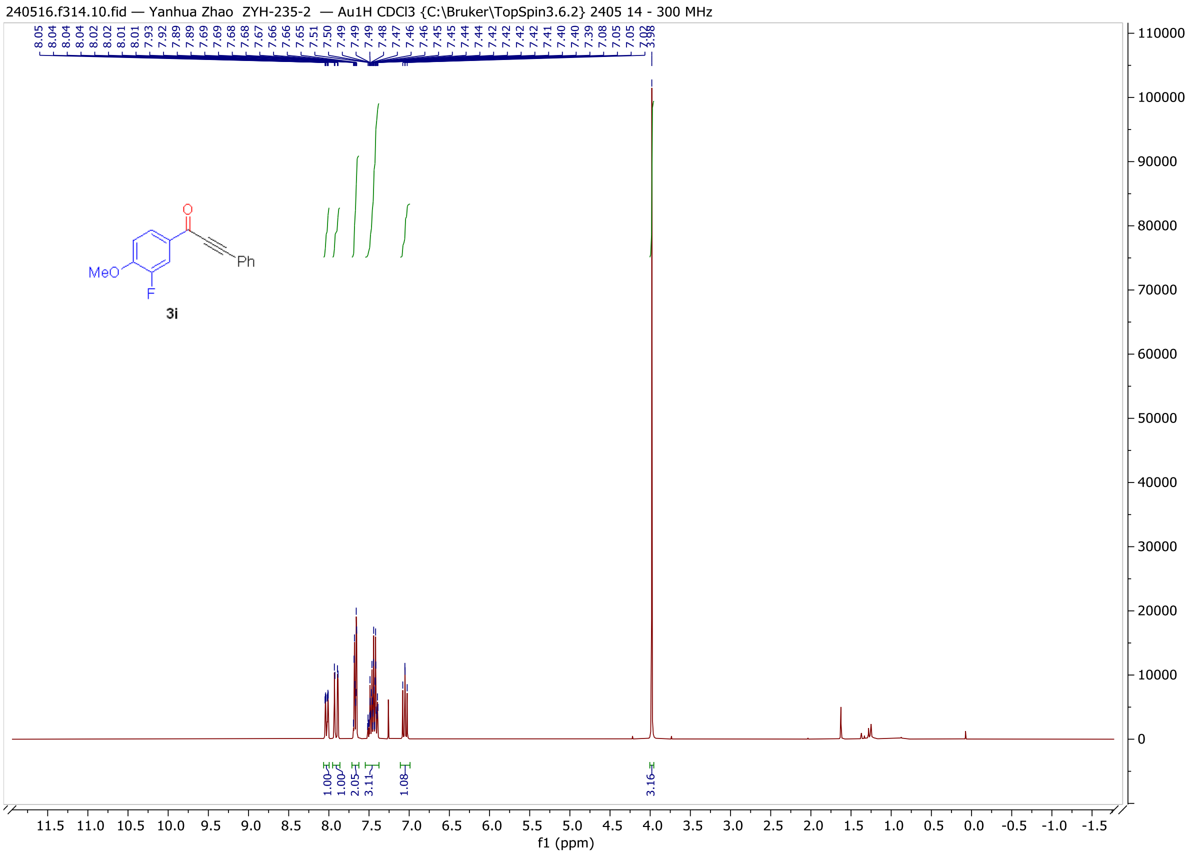
240516.f314.10.fid — Yanhua Zhao ZYH-235-2 — Au1H CDCl3 {C:\Bruker\TopSpin3.6.2} 2405 14 - 300 MHz
MeO
F
O
Ph
3i
f1 (ppm)

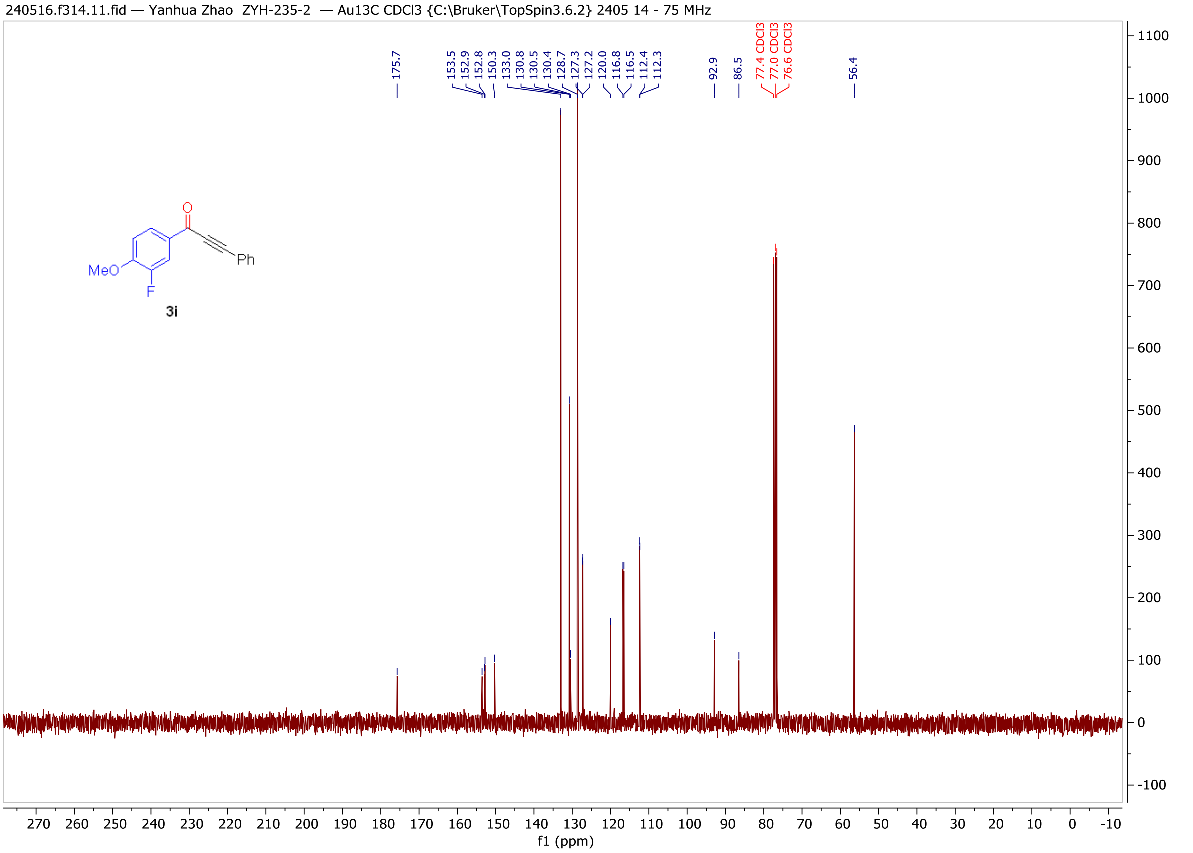
240516.f314.11.fid — Yanhua Zhao ZYH-235-2 — Au13C CDCl3 {C:\Bruker\TopSpin3.6.2} 2405 14 - 75 MHz
MeO
F
O
Ph
3i
f1 (ppm)

240516.f314.12.fid — Yanhua Zhao ZYH-235-2 — Au19F(H-gek) CDCl3 {C:\Bruker\TopSpin3.6.2} 2405 14-282 MHz

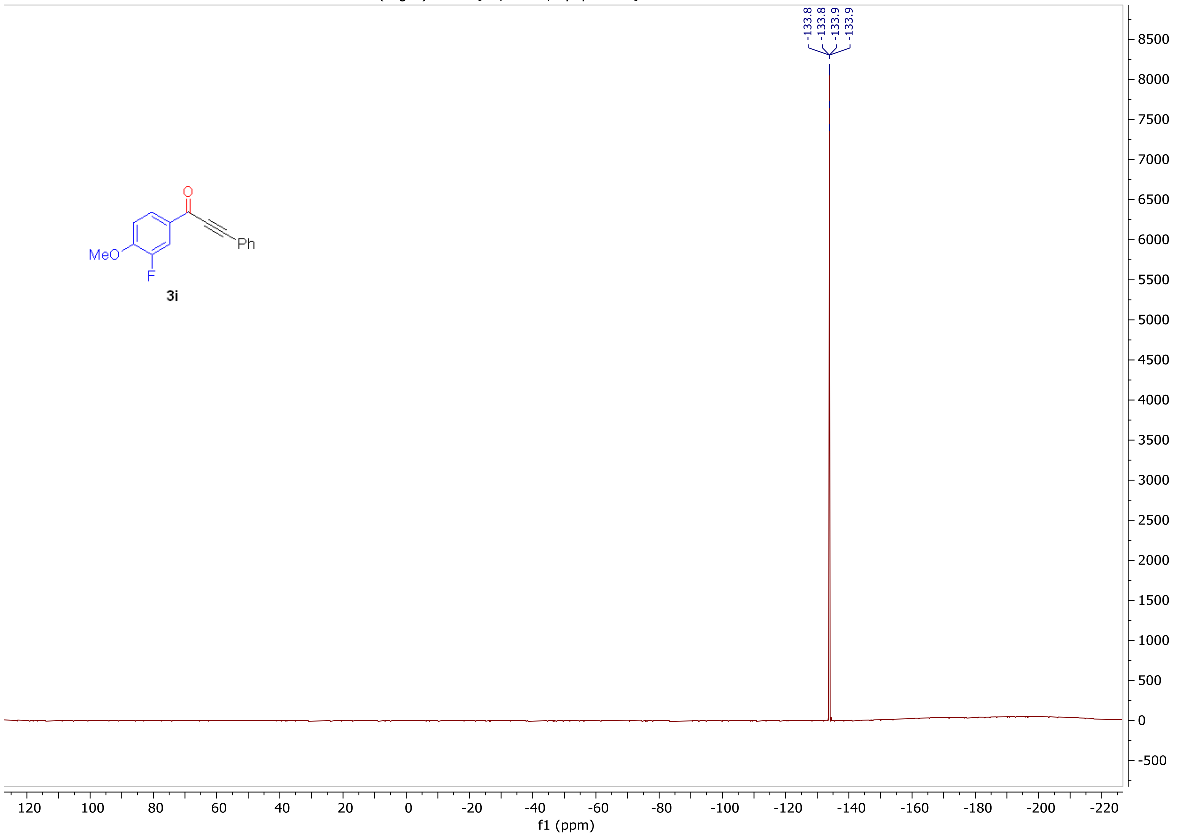

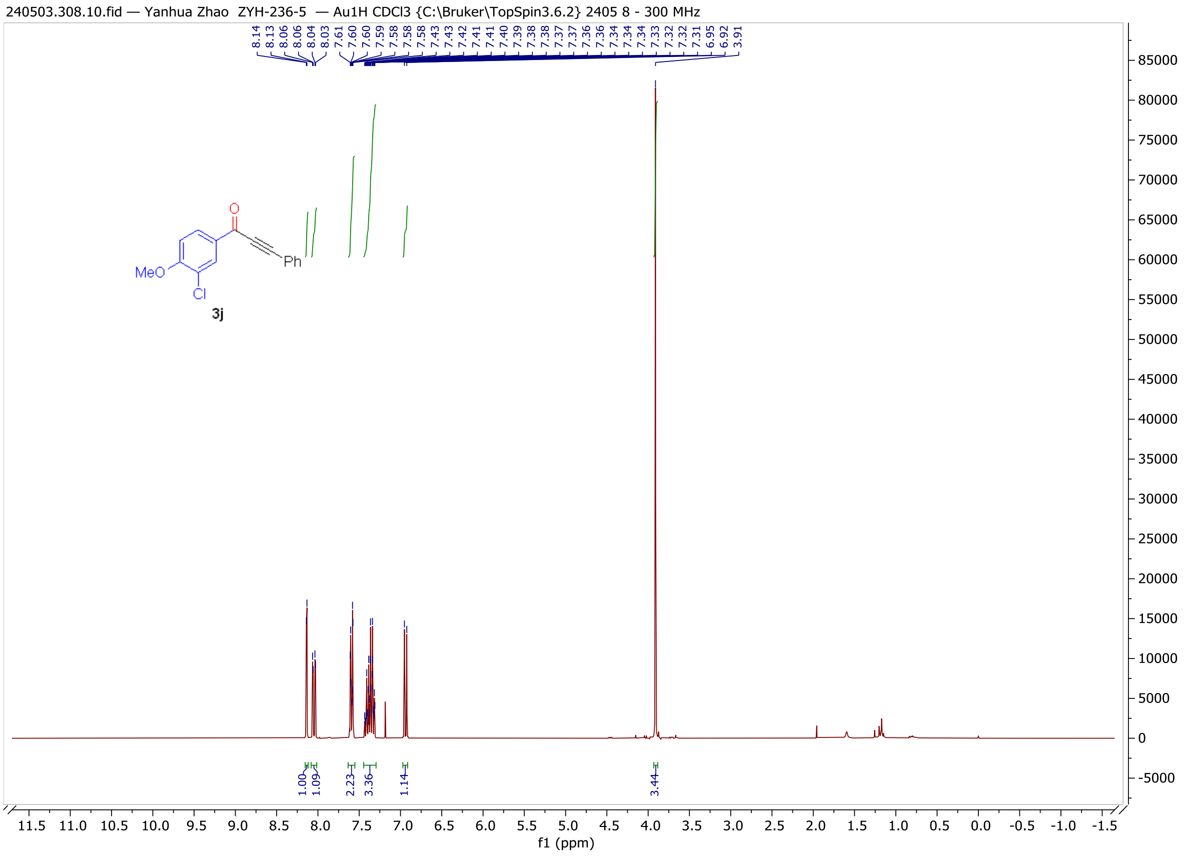
240503.308.10.fid — Yanhua Zhao ZYH-236-5 — Au1H CDCl3 {C:\Bruker\TopSpin3.6.2} 2405 8 - 300 MHz
8.14 8.13 8.06 8.06 8.04 8.03 7.61 7.60 7.60 7.59 7.58 7.58 7.58 7.43 7.43 7.42 7.41 7.41 7.40 7.39 7.38 7.38 7.37 7.37 7.36 7.34 7.34 7.34 7.33 7.32 7.32 7.31 6.95 6.92 3.91
MeO
Cl
O
Ph
3j
1.00 1.09 2.23 3.36 1.14 3.44
f1 (ppm)

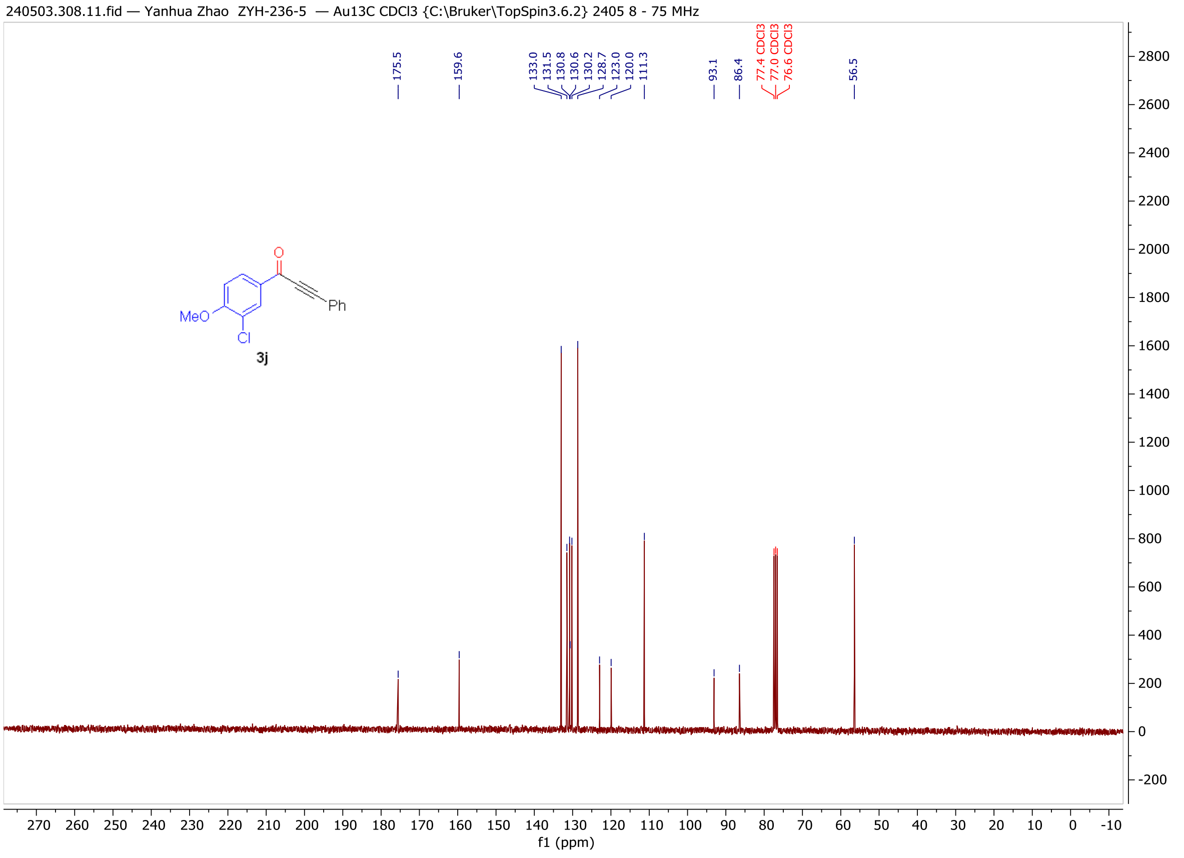
240503.308.11.fid — Yanhua Zhao ZYH-236-5 — Au13C CDCl3 {C:\Bruker\TopSpin3.6.2} 2405 8 - 75 MHz
175.5 159.6 133.0 131.5 130.8 130.6 130.2 128.7 123.0 120.0 111.3 93.1 86.4 77.4 CDCl3 77.0 CDCl3 76.6 CDCl3 56.5
MeO
Cl
O
Ph
3j
f1 (ppm)

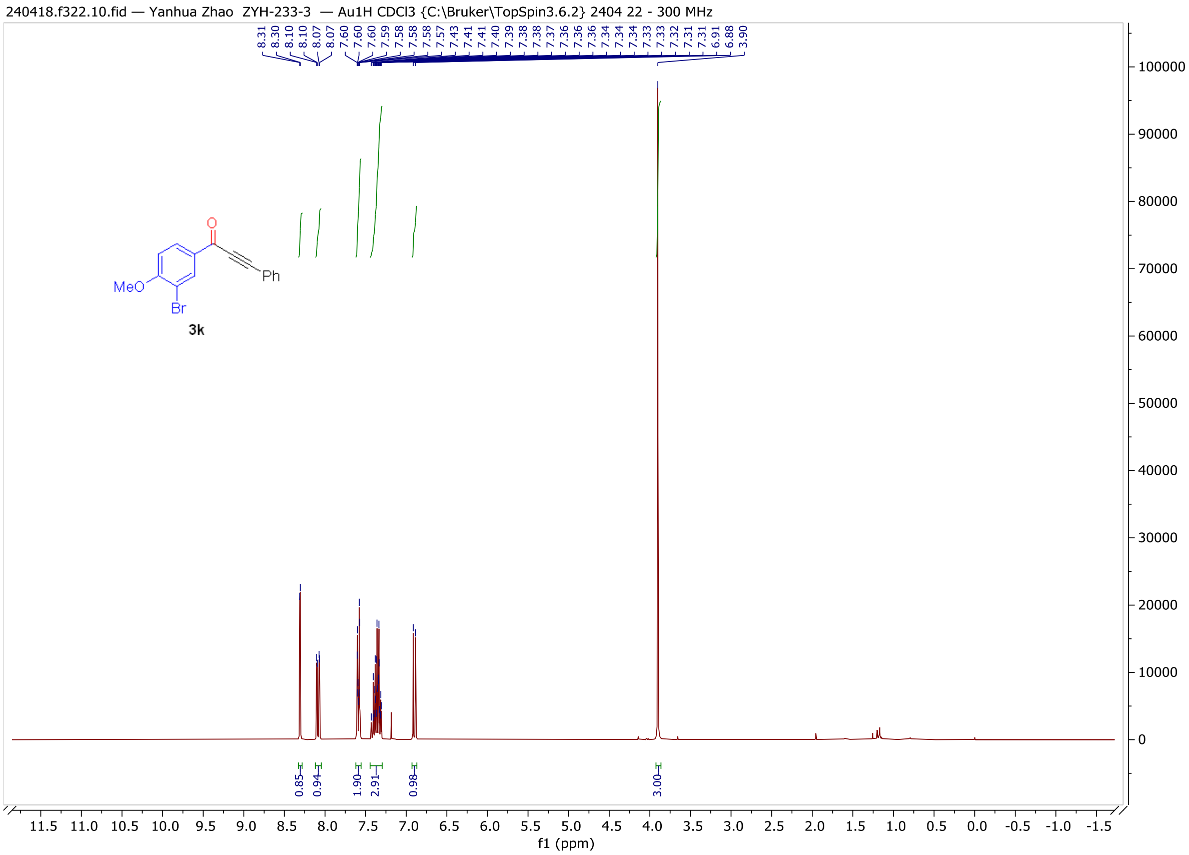
240418.f322.10.fid — Yanhua Zhao ZYH-233-3 — Au1H CDCl3 {C:\Bruker\TopSpin3.6.2} 2404 22 - 300 MHz
8.31 8.30 8.10 8.10 8.07 8.07 7.60 7.60 7.59 7.58 7.58 7.58 7.57 7.43 7.41 7.41 7.40 7.39 7.38 7.38 7.37 7.36 7.36 7.36 7.34 7.34 7.34 7.33 7.33 7.32 7.31 7.31 6.91 6.88 3.90
O
MeO
Br
Ph
3k
0.85
0.94
1.90
2.91
0.98
3.00
f1 (ppm)

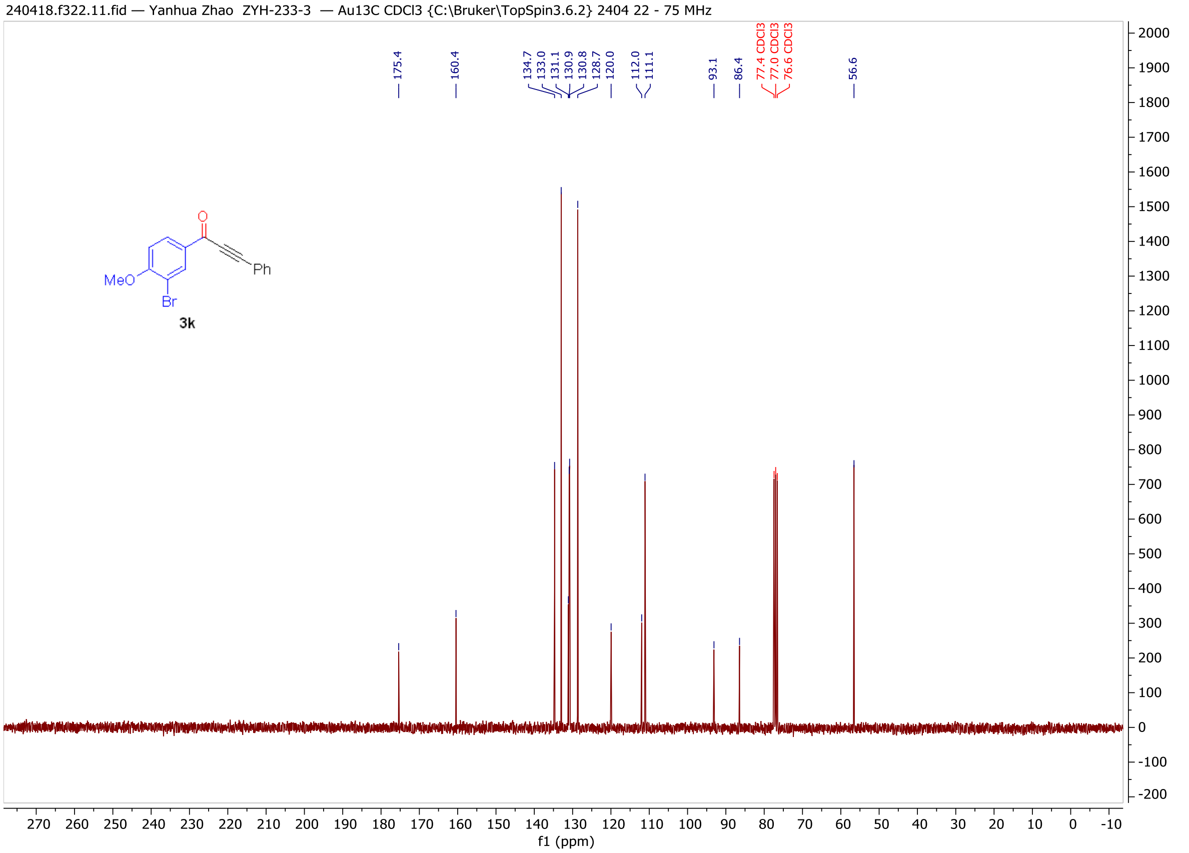
240418.f322.11.fid — Yanhua Zhao ZYH-233-3 — Au13C CDCl3 {C:\Bruker\TopSpin3.6.2} 2404 22 - 75 MHz
175.4
160.4
134.7 133.0 131.1 130.9 130.8 128.7 120.0
112.0 111.1
93.1
86.4
77.4 CDCl3 77.0 CDCl3 76.6 CDCl3
56.6
O
MeO
Br
Ph
3k
f1 (ppm)

240515.412.10.fid — Yanhua Zhao ZYH-244-3 — Au1H CDCl3 {C:\Bruker\TopSpin3.6.2} 2405 12 - 300 MHz

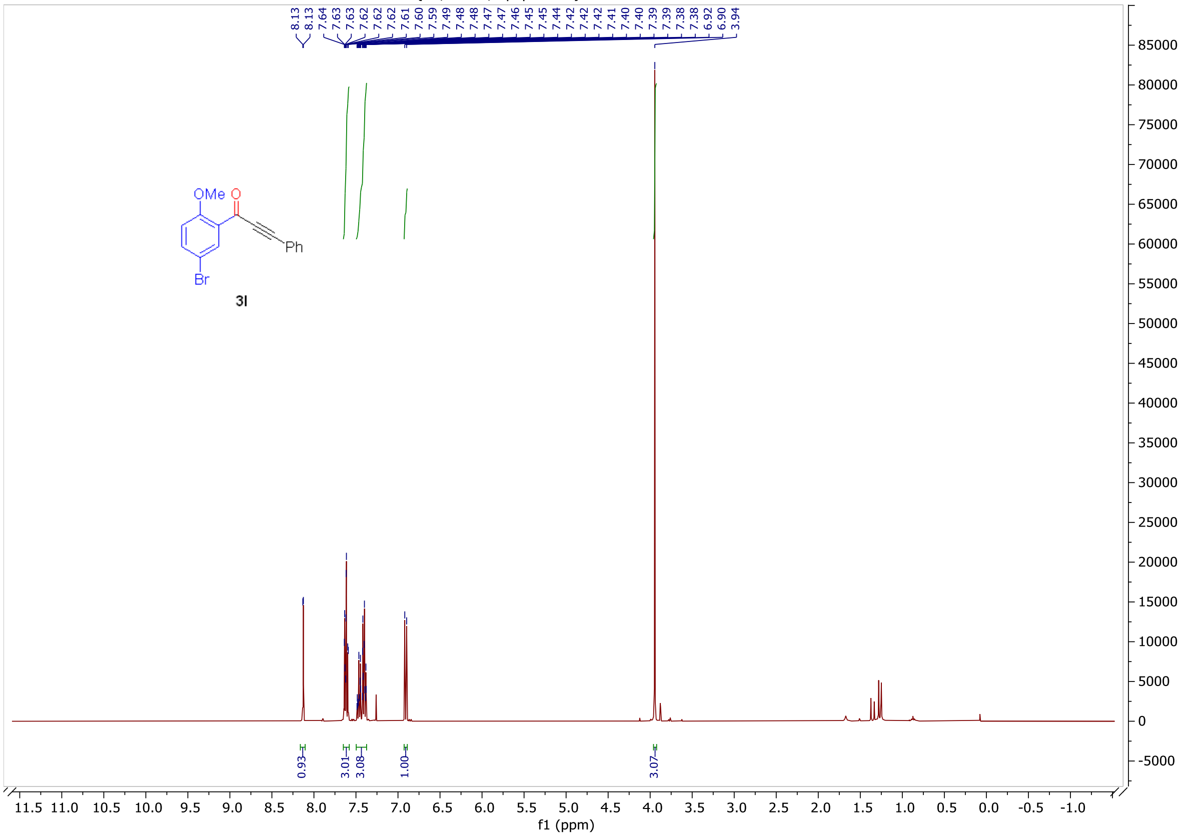

240515.412.11.fid — Yanhua Zhao ZYH-244-3 — Au13C CDCl3 {C:\Bruker\TopSpin3.6.2} 2405 12 - 75 MHz

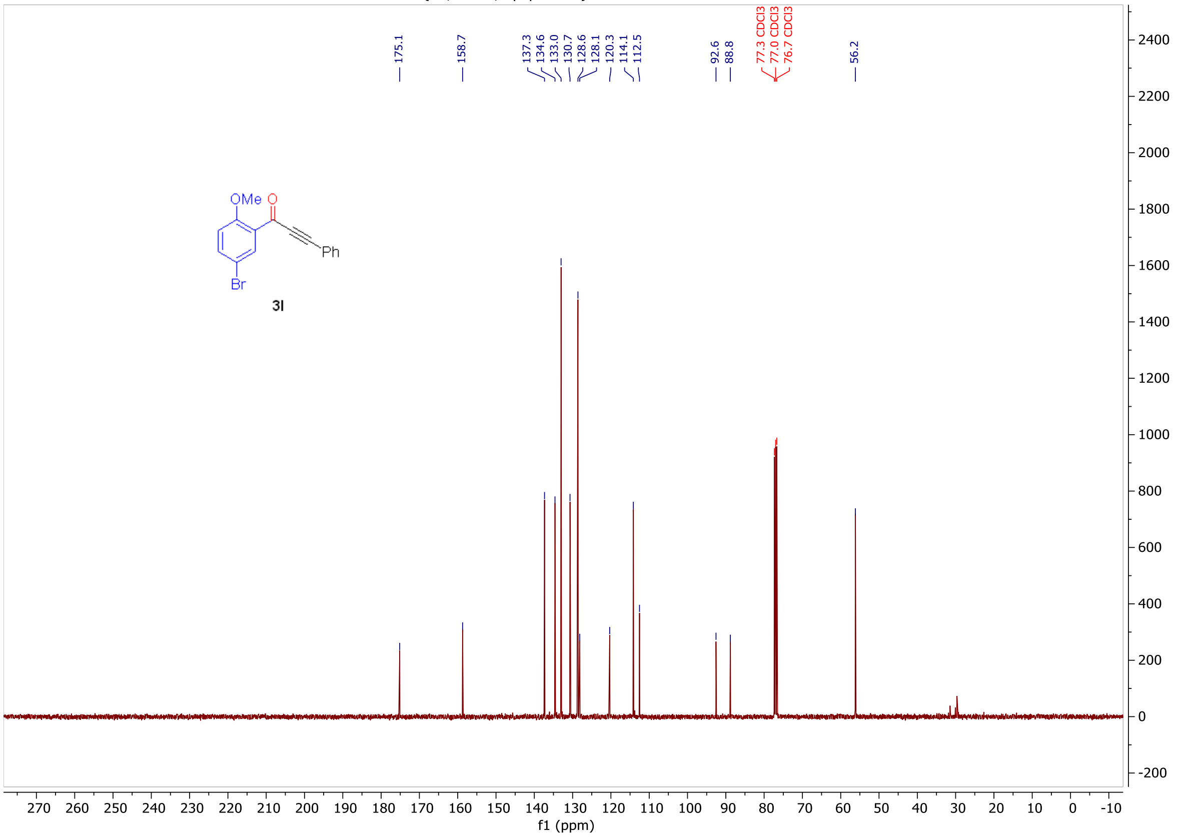

240426.f315.10.fid — Yanhua Zhao ZYH-236-3 — Au1H CDCl3 {C:\Bruker\TopSpin3.6.2} 2404 15 - 300 MHz

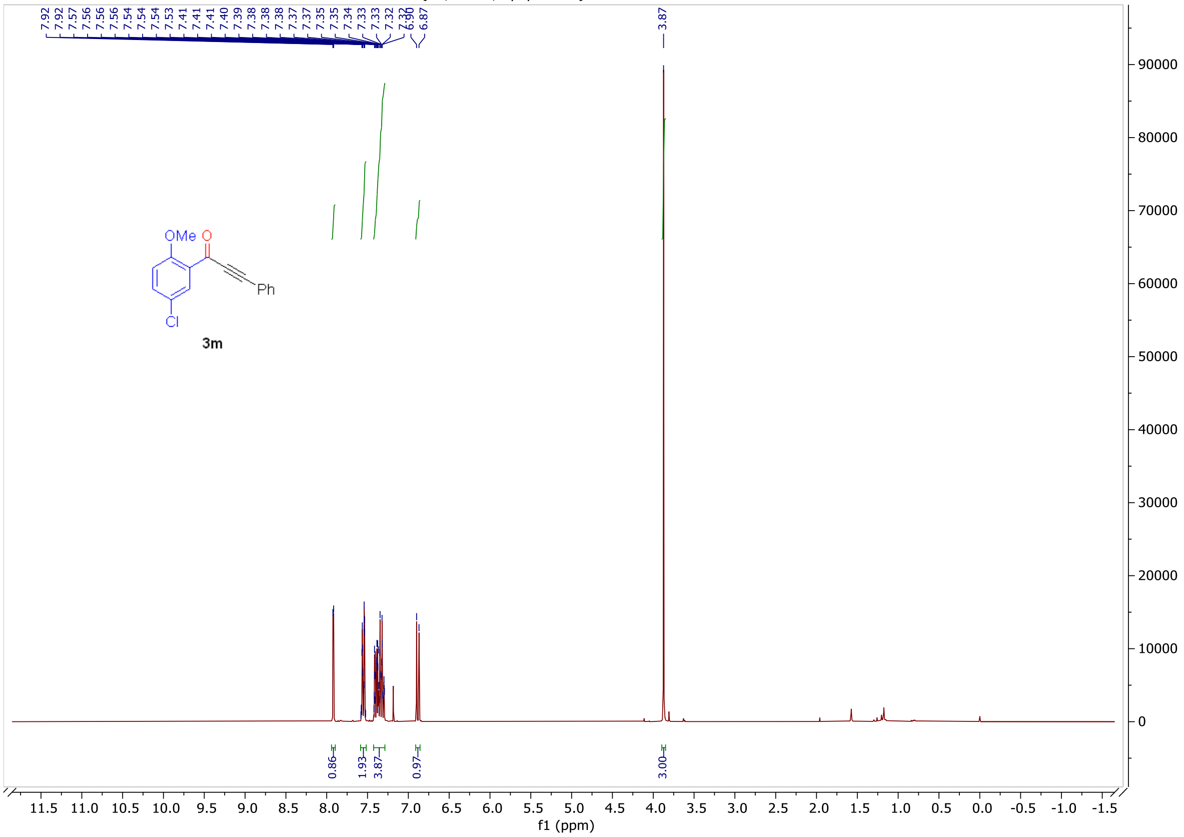

240426.f315.11.fid — Yanhua Zhao ZYH-236-3 — Au13C CDCl3 {C:\Bruker\TopSpin3.6.2} 2404 15 - 75 MHz

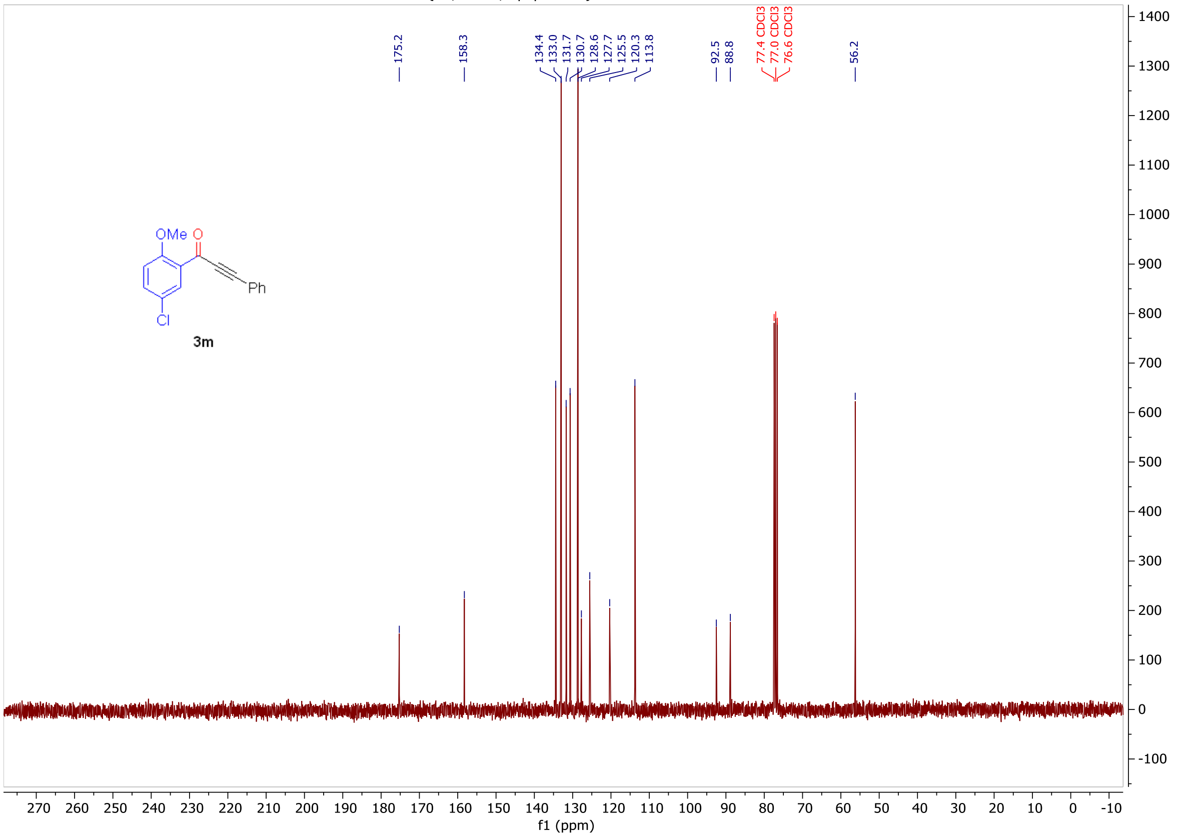

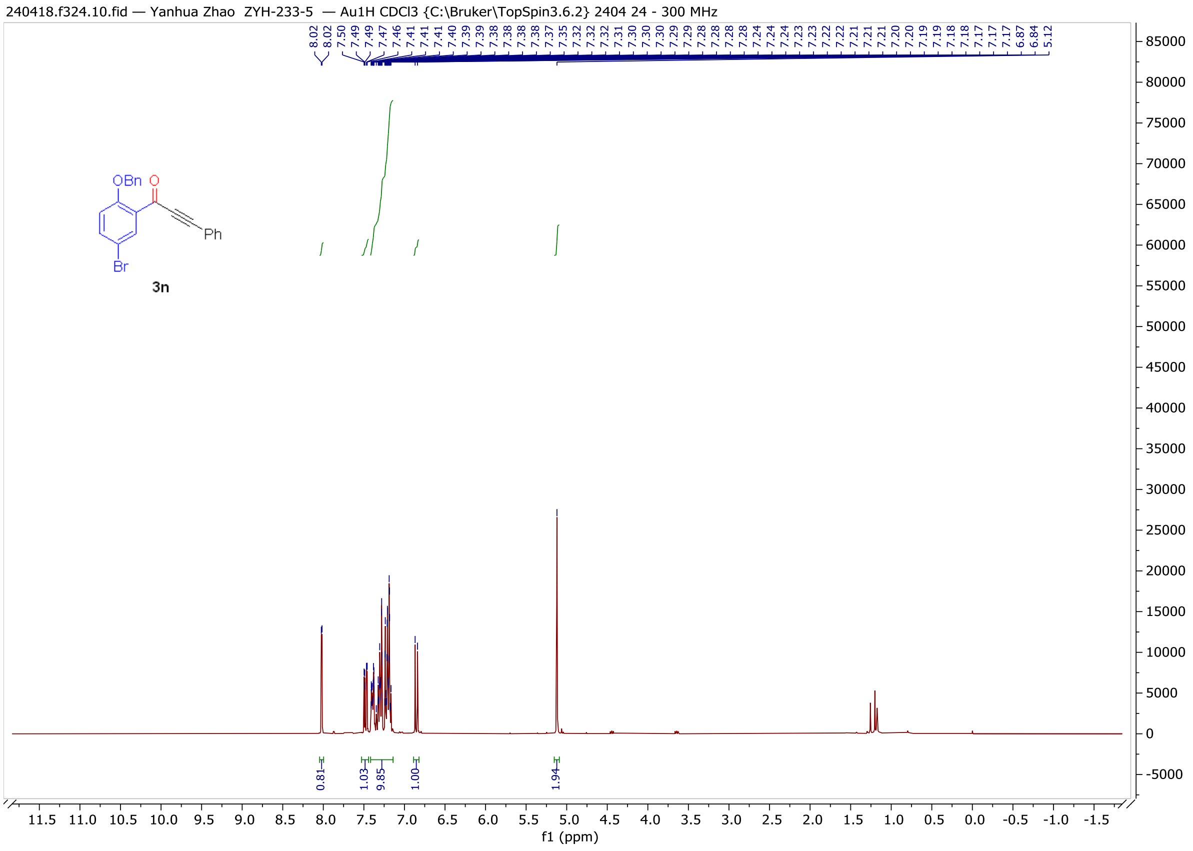
240418.f324.10.fid — Yanhua Zhao ZYH-233-5 — Au1H CDCl3 {C:\Bruker\TopSpin3.6.2} 2404 24 - 300 MHz
8.02 8.02 7.50 7.49 7.49 7.47 7.46 7.41 7.41 7.40 7.39 7.39 7.38 7.38 7.38 7.38 7.37 7.35 7.32 7.32 7.32 7.31 7.30 7.30 7.30 7.29 7.28 7.28 7.28 7.24 7.24 7.24 7.23 7.23 7.22 7.22 7.21 7.21 7.21 7.20 7.20 7.19 7.19 7.18 7.17 7.17 7.17 6.87 6.84 5.12
OBn
O
Ph
Br
3n
0.81 1.03 9.85 1.00 1.94
f1 (ppm)

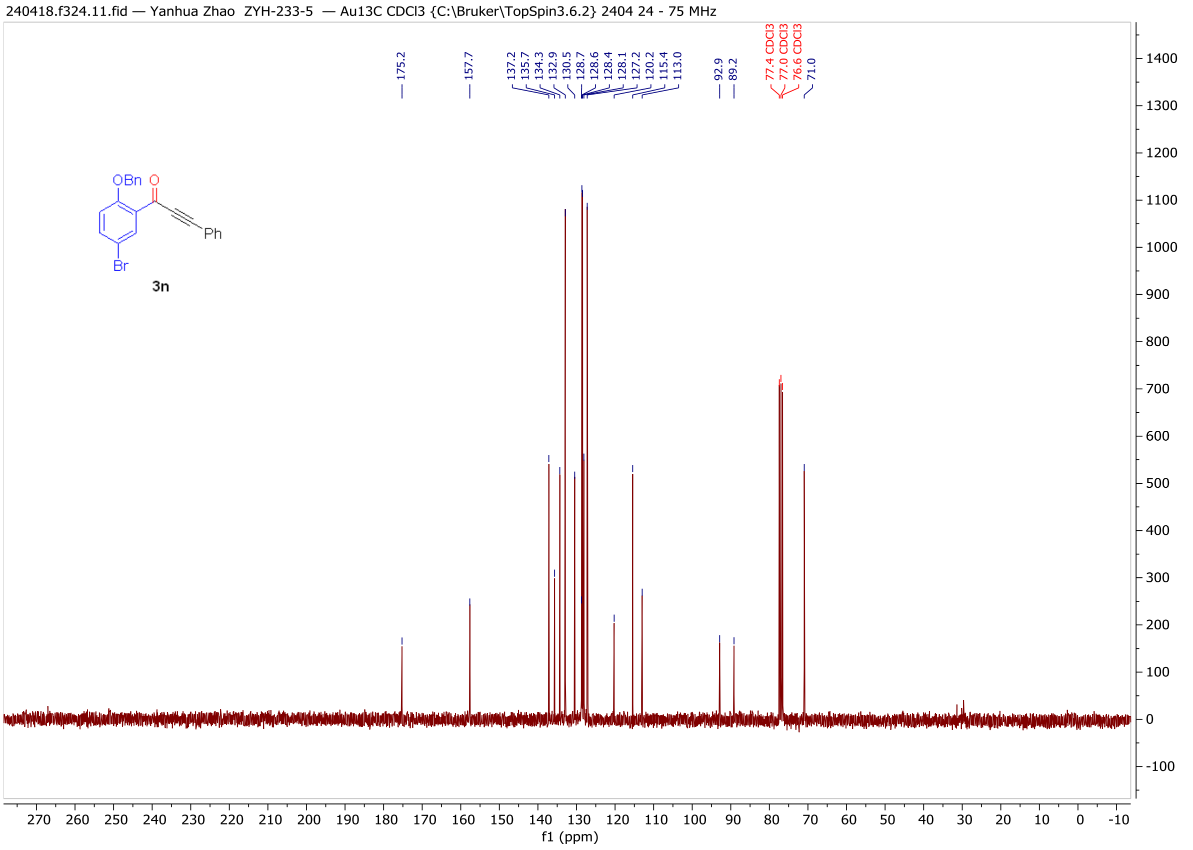
240418.f324.11.fid — Yanhua Zhao ZYH-233-5 — Au13C CDCl3 {C:\Bruker\TopSpin3.6.2} 2404 24 - 75 MHz
175.2 157.7 137.2 135.7 134.3 132.9 130.5 128.7 128.6 128.4 128.1 127.2 120.2 115.4 113.0 92.9 89.2
77.4 CDCl3 77.0 CDCl3 76.6 CDCl3 71.0
OBn
O
Ph
Br
3n
f1 (ppm)

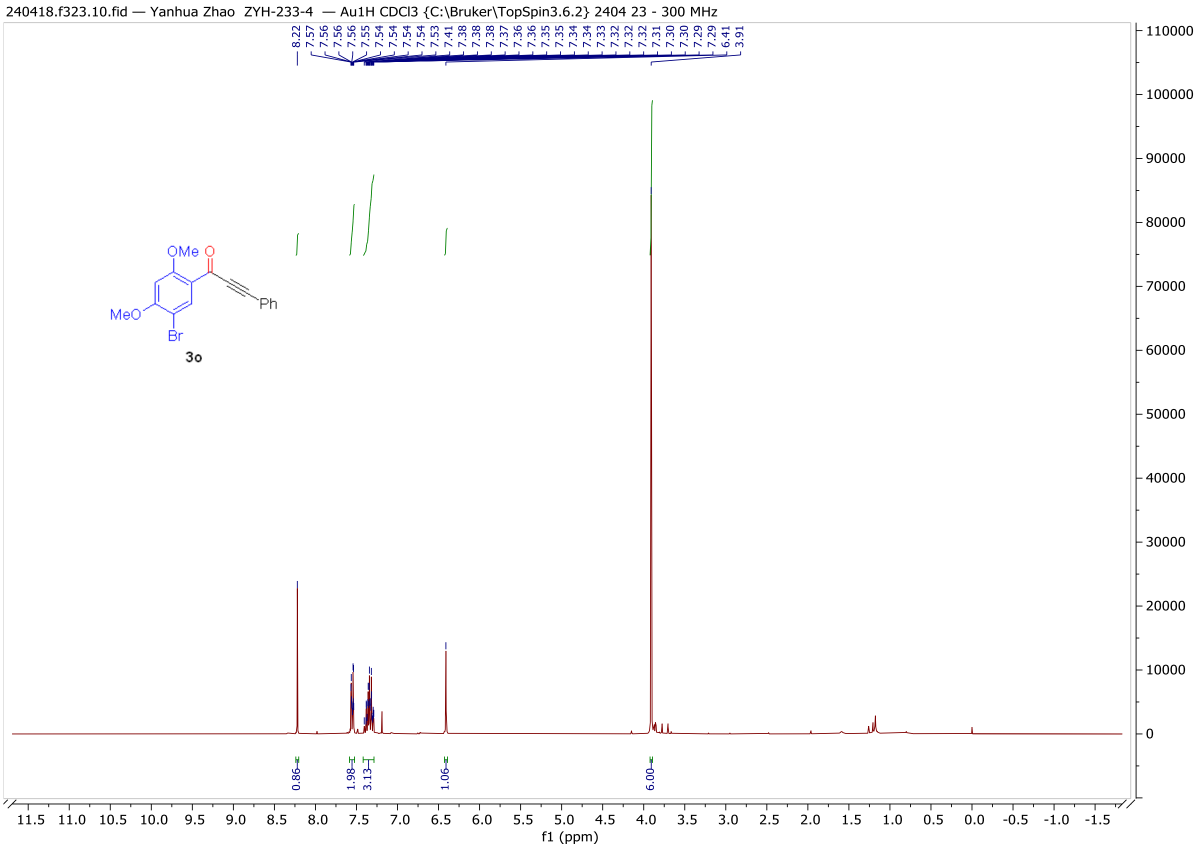
240418.f323.10.fid — Yanhua Zhao ZYH-233-4 — Au1H CDCl3 {C:\Bruker\TopSpin3.6.2} 2404 23 - 300 MHz
8.22 7.57 7.56 7.56 7.56 7.55 7.54 7.54 7.54 7.54 7.53 7.41 7.38 7.38 7.38 7.37 7.36 7.36 7.35 7.35 7.34 7.34 7.33 7.32 7.32 7.32 7.31 7.30 7.30 7.29 7.29 6.41 3.91
OMe O
MeO
Br
Ph
3o
0.86 1.98 3.13 1.06 6.00
f1 (ppm)

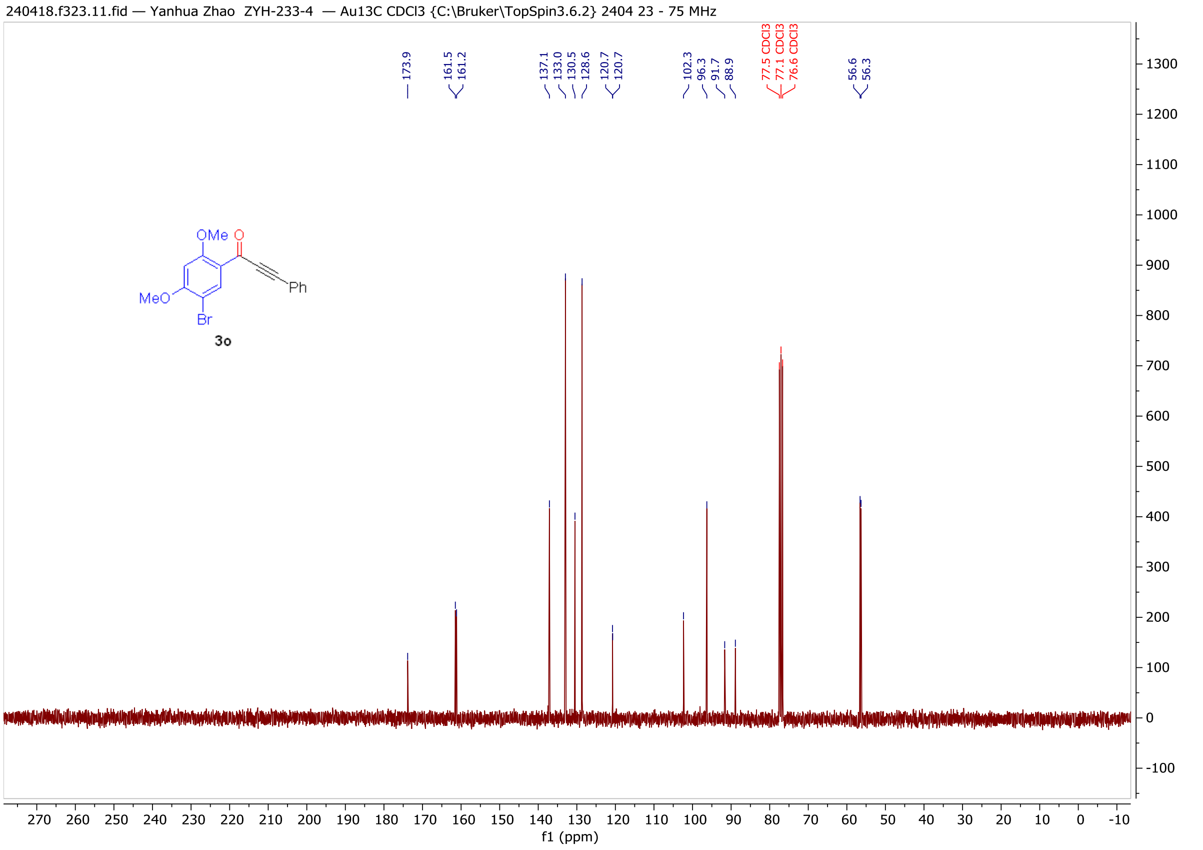
240418.f323.11.fid — Yanhua Zhao ZYH-233-4 — Au13C CDCl3 {C:\Bruker\TopSpin3.6.2} 2404 23 - 75 MHz
173.9 161.5 161.2 137.1 133.0 130.5 128.6 120.7 120.7 102.3 96.3 91.7 88.9 77.5 CDCl3 77.1 CDCl3 76.6 CDCl3 56.6 56.3
OMe O
MeO
Br
Ph
3o
f1 (ppm)

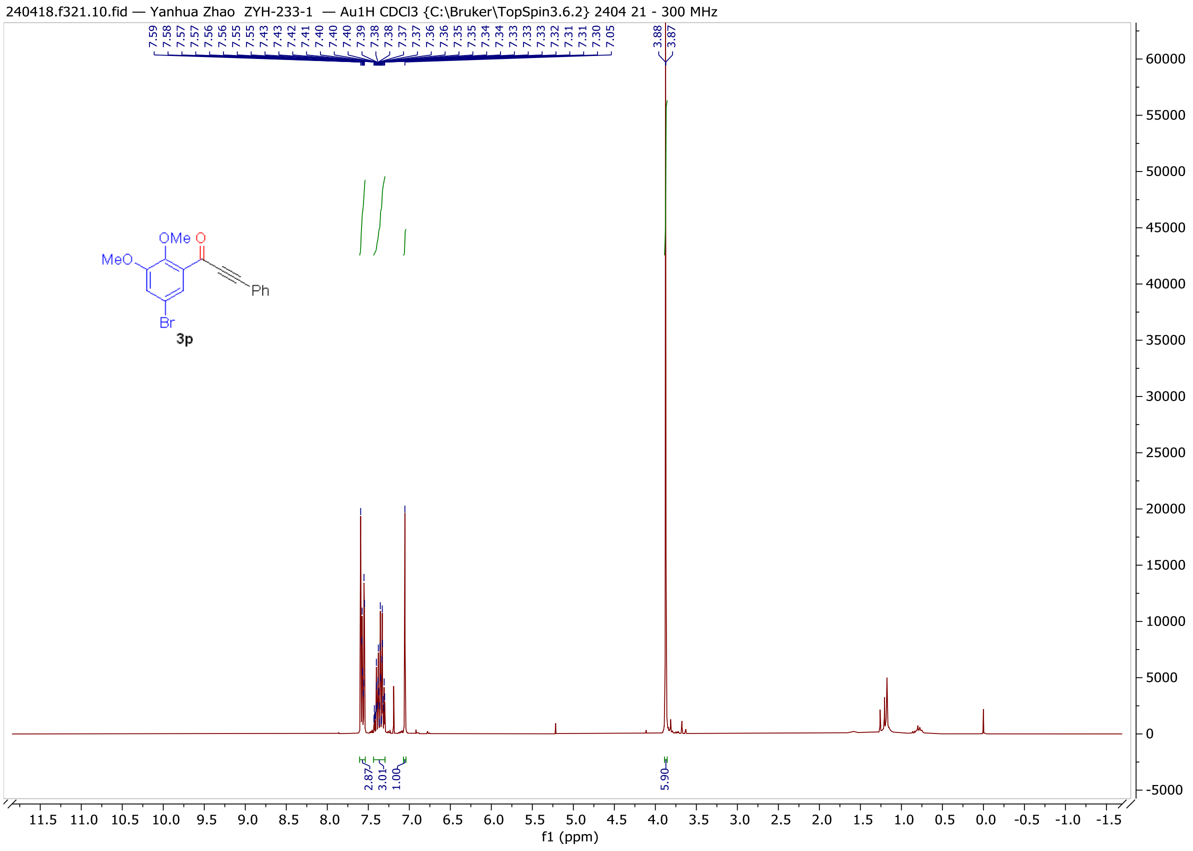
240418.f321.10.fid — Yanhua Zhao ZYH-233-1 — Au1H CDCl3 {C:\Bruker\TopSpin3.6.2} 2404 21 - 300 MHz
7.59 7.58 7.57 7.57 7.56 7.55 7.55 7.43 7.43 7.42 7.41 7.40 7.40 7.39 7.38 7.38 7.37 7.37 7.36 7.36 7.35 7.35 7.34 7.34 7.33 7.33 7.33 7.32 7.31 7.31 7.30 7.05
3.88 3.87
OMe
MeO
O
Ph
Br
3p
2.87
3.01
1.00
5.90
f1 (ppm)

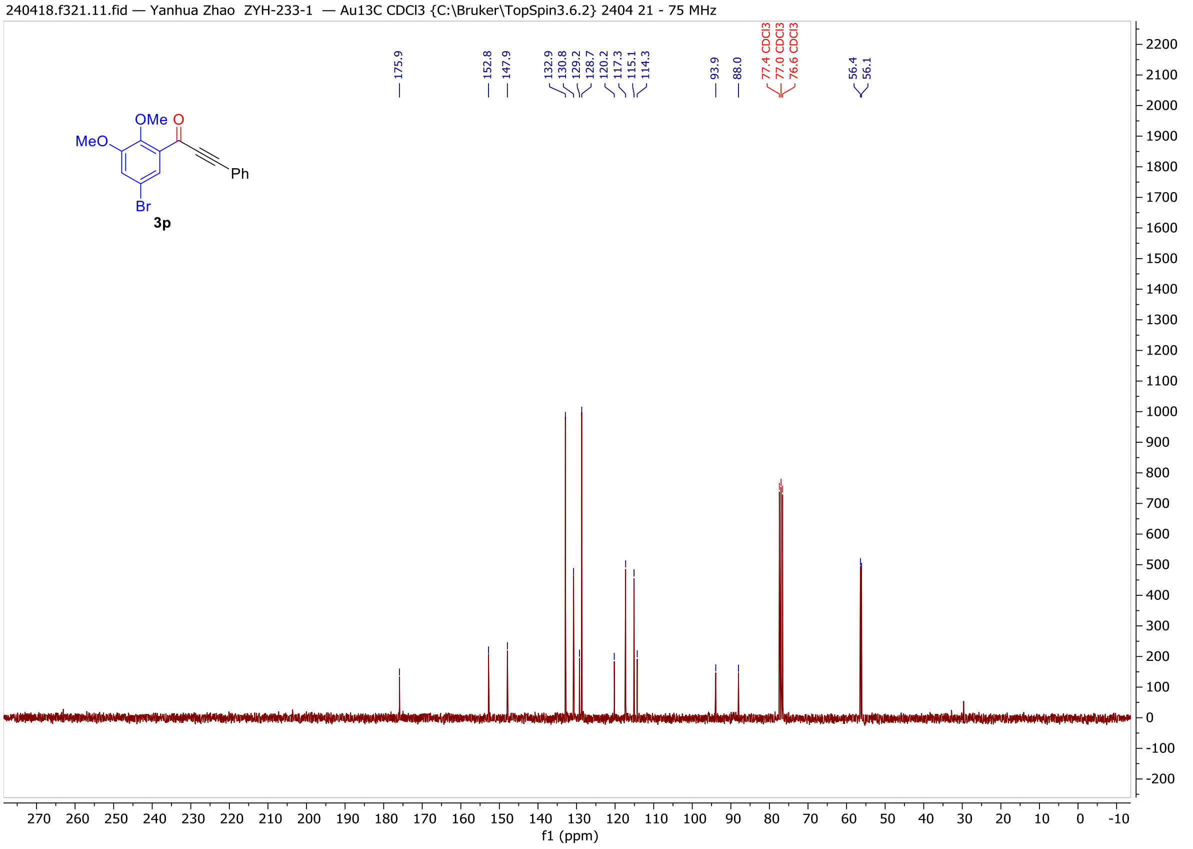
240418.f321.11.fid — Yanhua Zhao ZYH-233-1 — Au13C CDCl3 {C:\Bruker\TopSpin3.6.2} 2404 21 - 75 MHz
175.9
152.8 147.9
132.9 130.8 129.2 128.7 120.2 117.3 115.1 114.3
93.9 88.0
77.4 CDCl3 77.0 CDCl3 76.6 CDCl3
56.4 56.1
OMe
MeO
O
Ph
Br
3p
f1 (ppm)

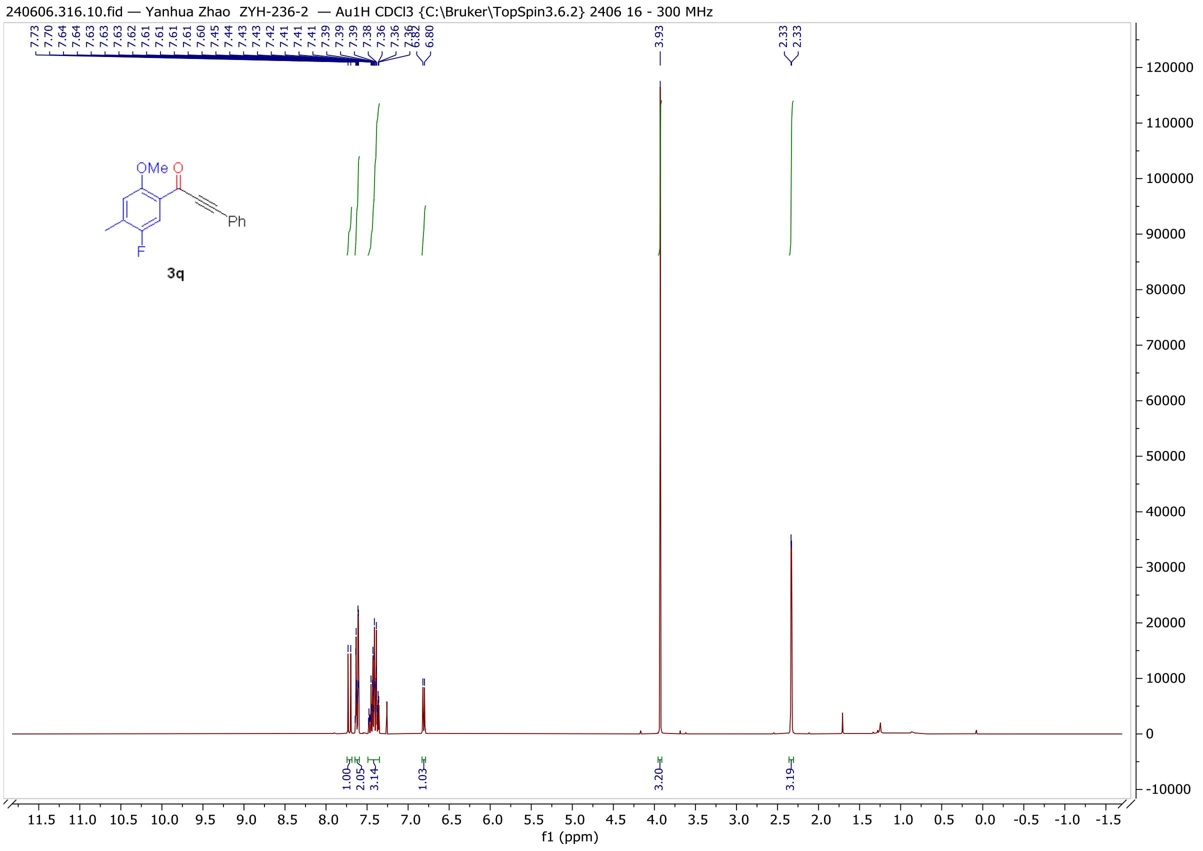
240606.316.10.fid — Yanhua Zhao ZYH-236-2 — Au1H CDCl3 {C:\Bruker\TopSpin3.6.2} 2406 16 - 300 MHz
3q
f1 (ppm)

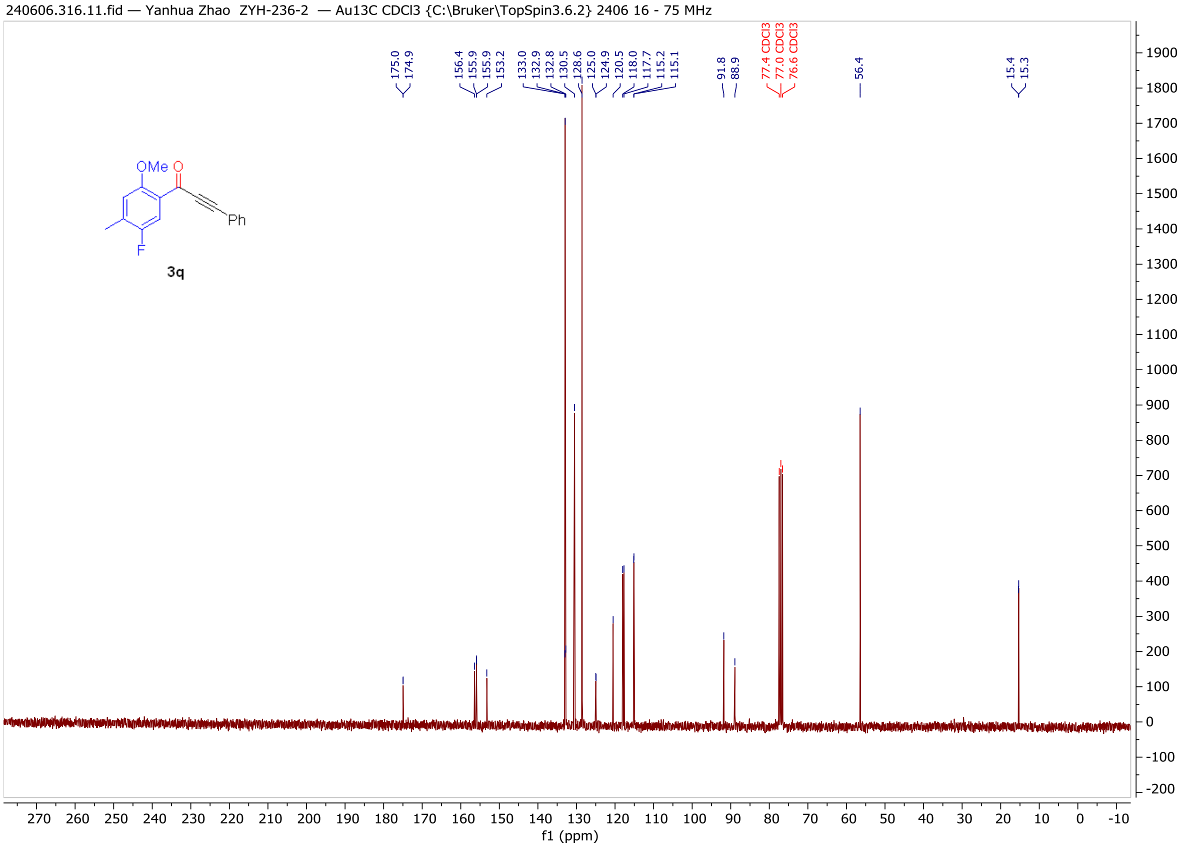
240606.316.11.fid — Yanhua Zhao ZYH-236-2 — Au13C CDCl3 {C:\Bruker\TopSpin3.6.2} 2406 16 - 75 MHz
3q
f1 (ppm)

240606.316.12.fid — Yanhua Zhao ZYH-236-2 — Au19F CDCl3 {C:\Bruker\TopSpin3.6.2} 2406 16-282 MHz

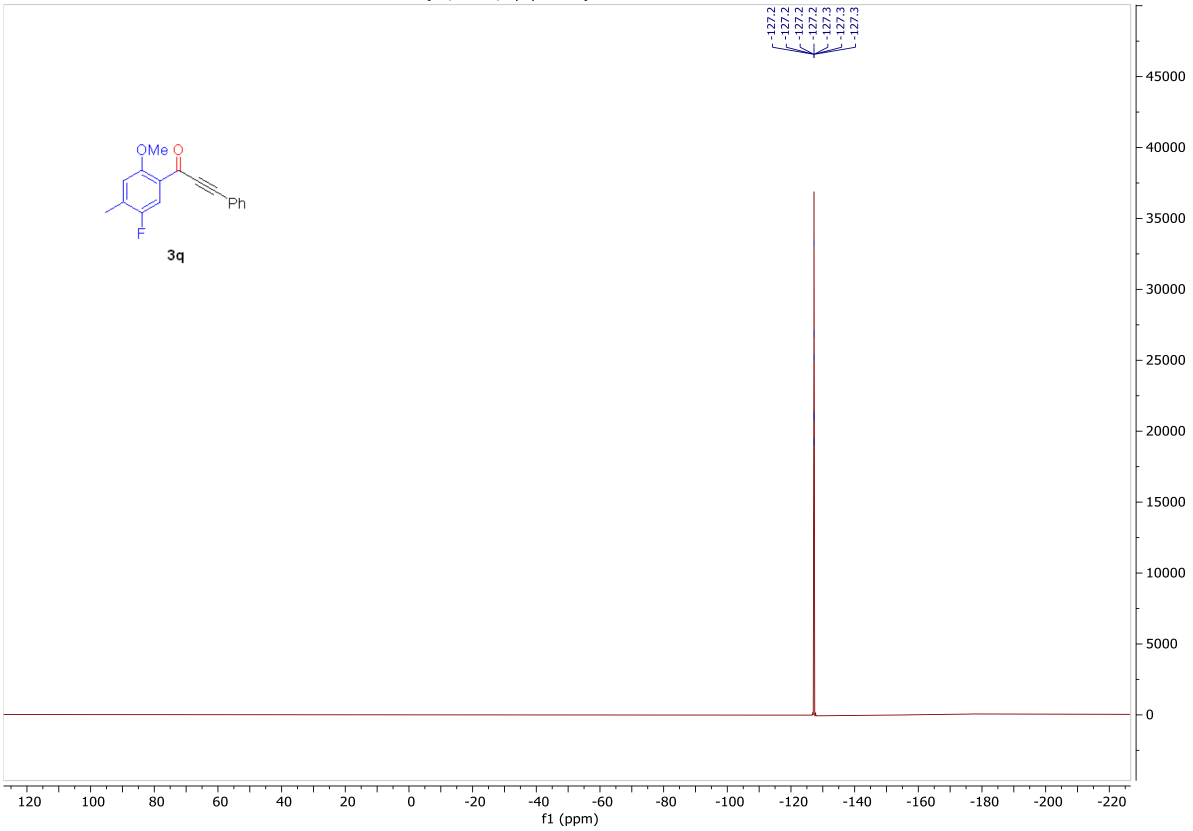

3q

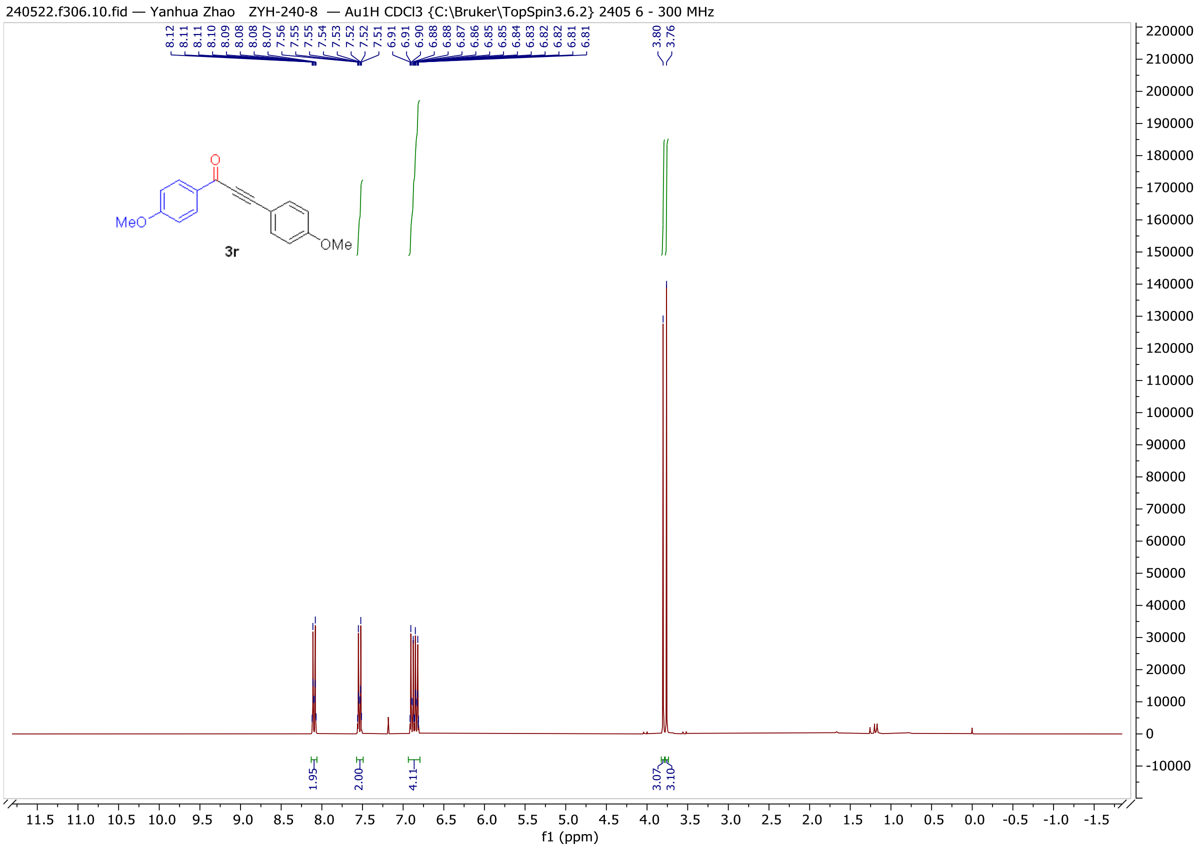
240522.f306.10.fid — Yanhua Zhao ZYH-240-8 — Au1H CDCl3 {C:\Bruker\TopSpin3.6.2} 2405 6 - 300 MHz
8.12 8.11 8.11 8.10 8.09 8.08 8.08 8.07 7.56 7.55 7.55 7.54 7.53 7.52 7.52 7.51 6.91 6.91 6.90 6.88 6.88 6.87 6.86 6.85 6.85 6.84 6.83 6.82 6.82 6.81 6.81
3.80 3.76
MeO
OMe
3r
1.95
2.00
4.11
3.07
3.10
f1 (ppm)

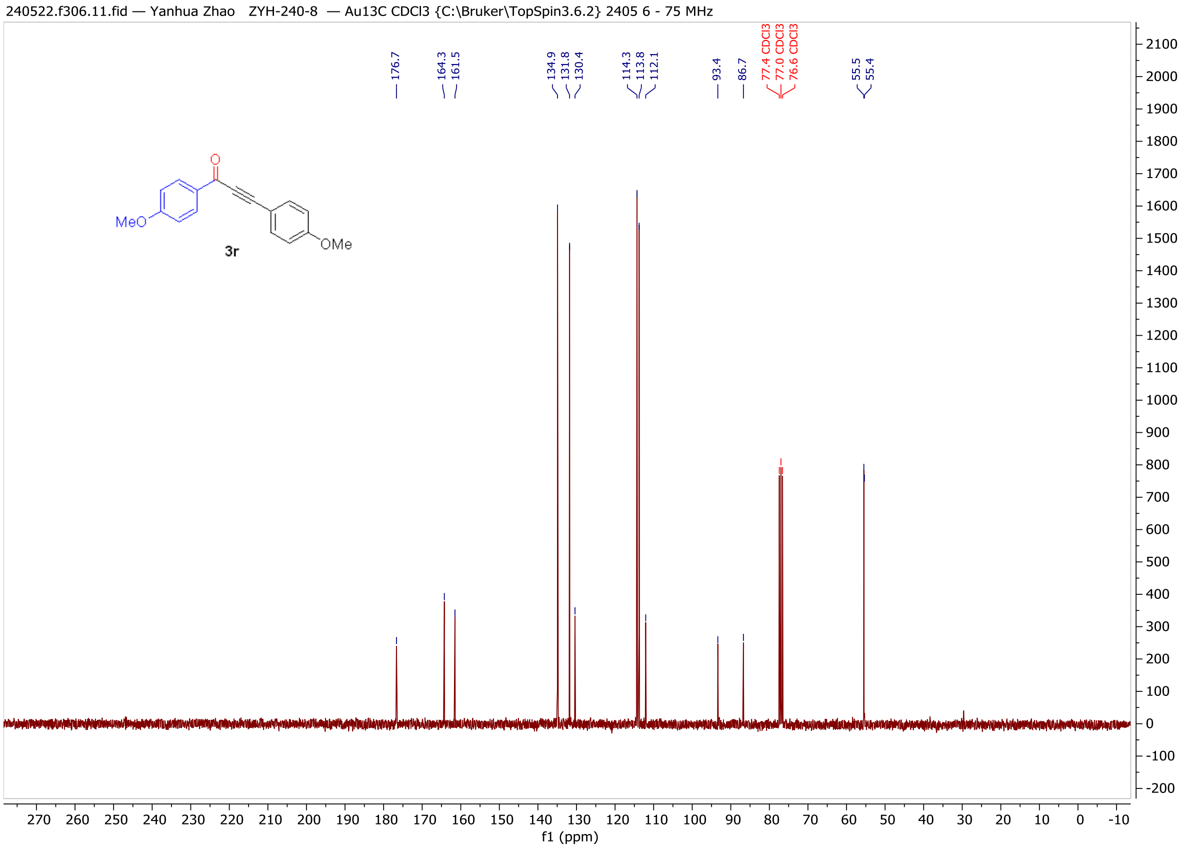
240522.f306.11.fid — Yanhua Zhao ZYH-240-8 — Au13C CDCl3 {C:\Bruker\TopSpin3.6.2} 2405 6 - 75 MHz
176.7
164.3 161.5
134.9 131.8 130.4
114.3 113.8 112.1
93.4
86.7
77.4 CDCl3 77.0 CDCl3 76.6 CDCl3
55.5 55.4
MeO
OMe
3r
f1 (ppm)

240503.310.10.fid — Yanhua Zhao ZYH-240-1 — Au1H CDCl3 {C:\Bruker\TopSpin3.6.2} 2405 10 - 300 MHz

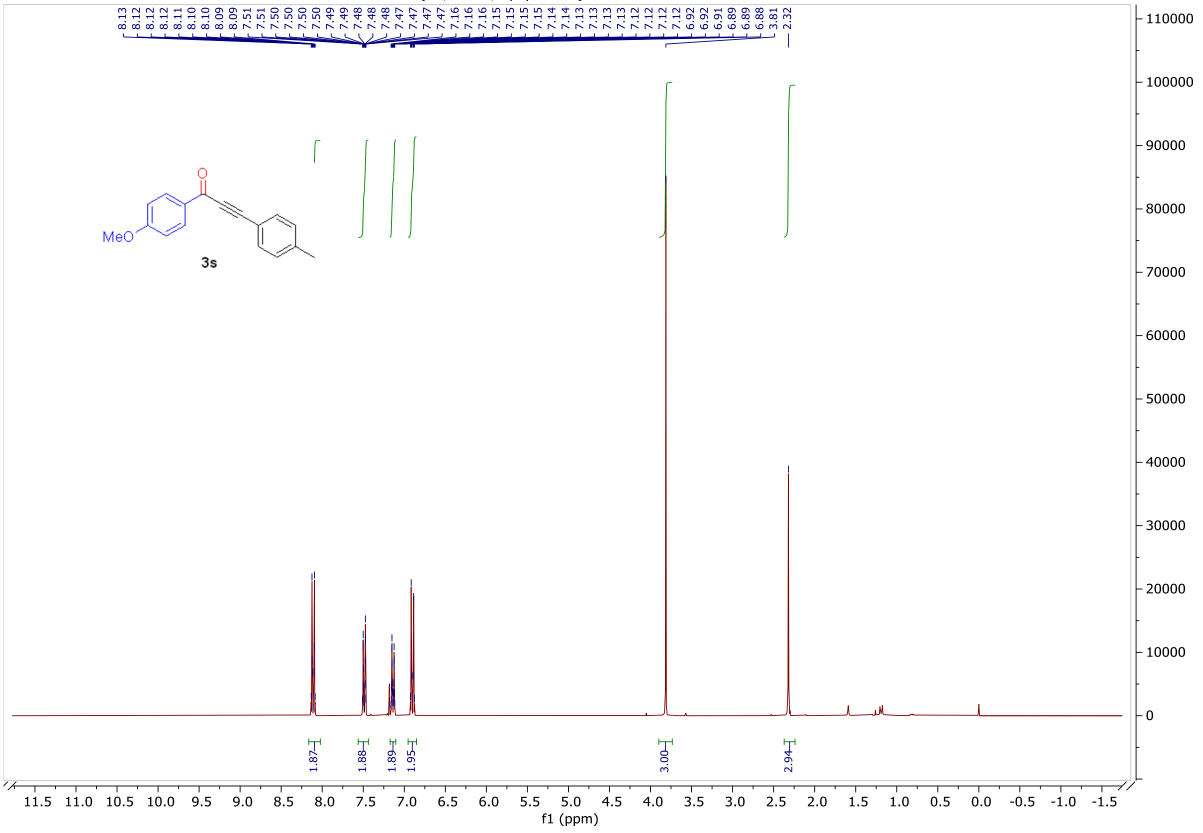

3s

240503.310.11.fid — Yanhua Zhao ZYH-240-1 — Au13C CDCl3 {C:\Bruker\TopSpin3.6.2} 2405 10 - 75 MHz

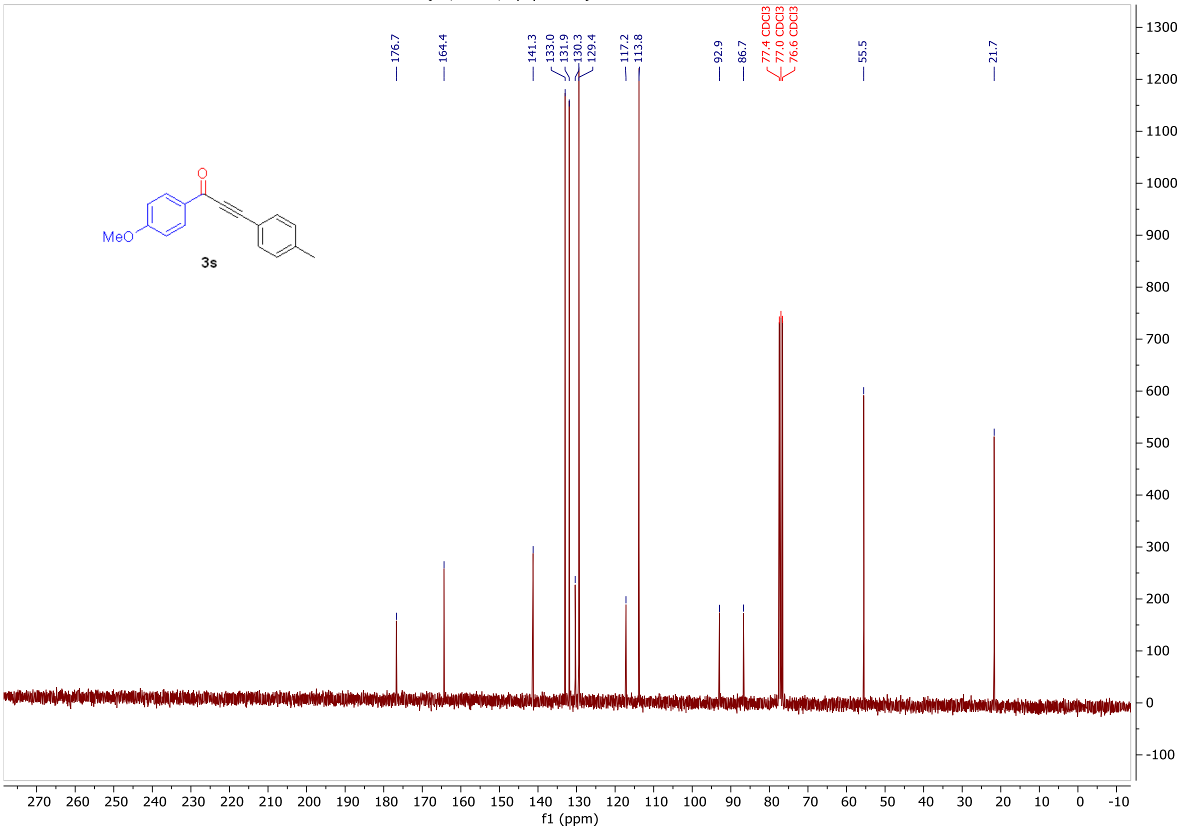

3s

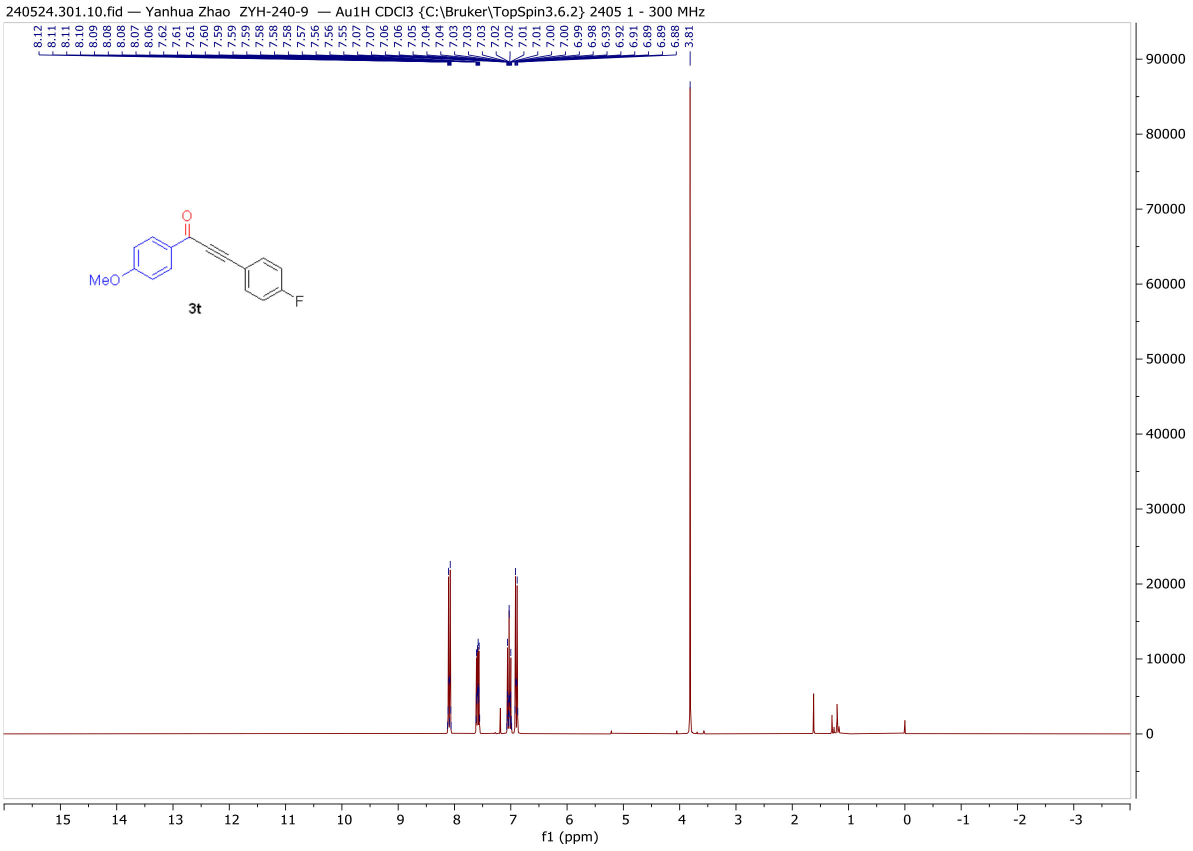
240524.301.10.fid — Yanhua Zhao ZYH-240-9 — Au1H CDCl3 {C:\Bruker\TopSpin3.6.2} 2405 1 - 300 MHz
MeO
O
F
3t
f1 (ppm)

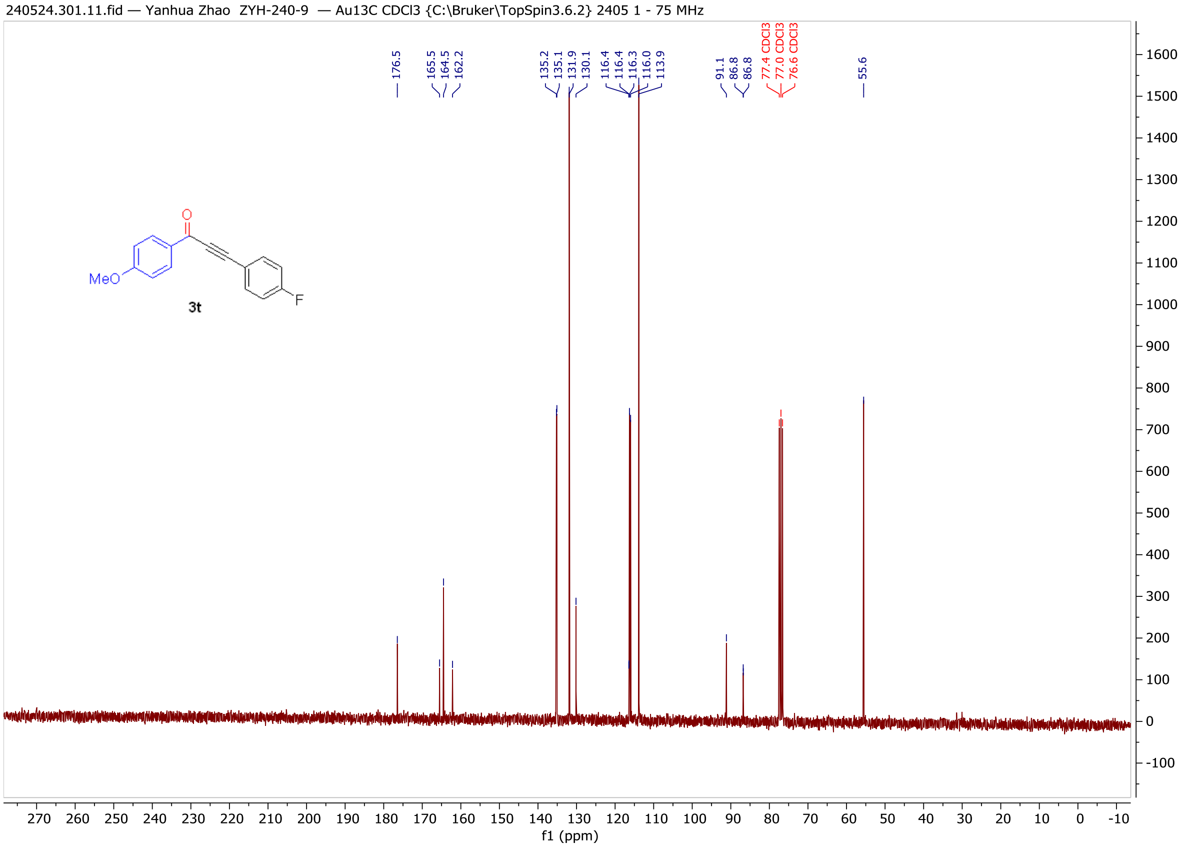
240524.301.11.fid — Yanhua Zhao ZYH-240-9 — Au13C CDCl3 {C:\Bruker\TopSpin3.6.2} 2405 1 - 75 MHz
176.5
165.5
164.5
162.2
135.2
135.1
131.9
130.1
116.4
116.4
116.3
116.0
113.9
91.1
86.8
86.8
77.4 CDCl3
77.0 CDCl3
76.6 CDCl3
55.6
MeO
O
F
3t
f1 (ppm)

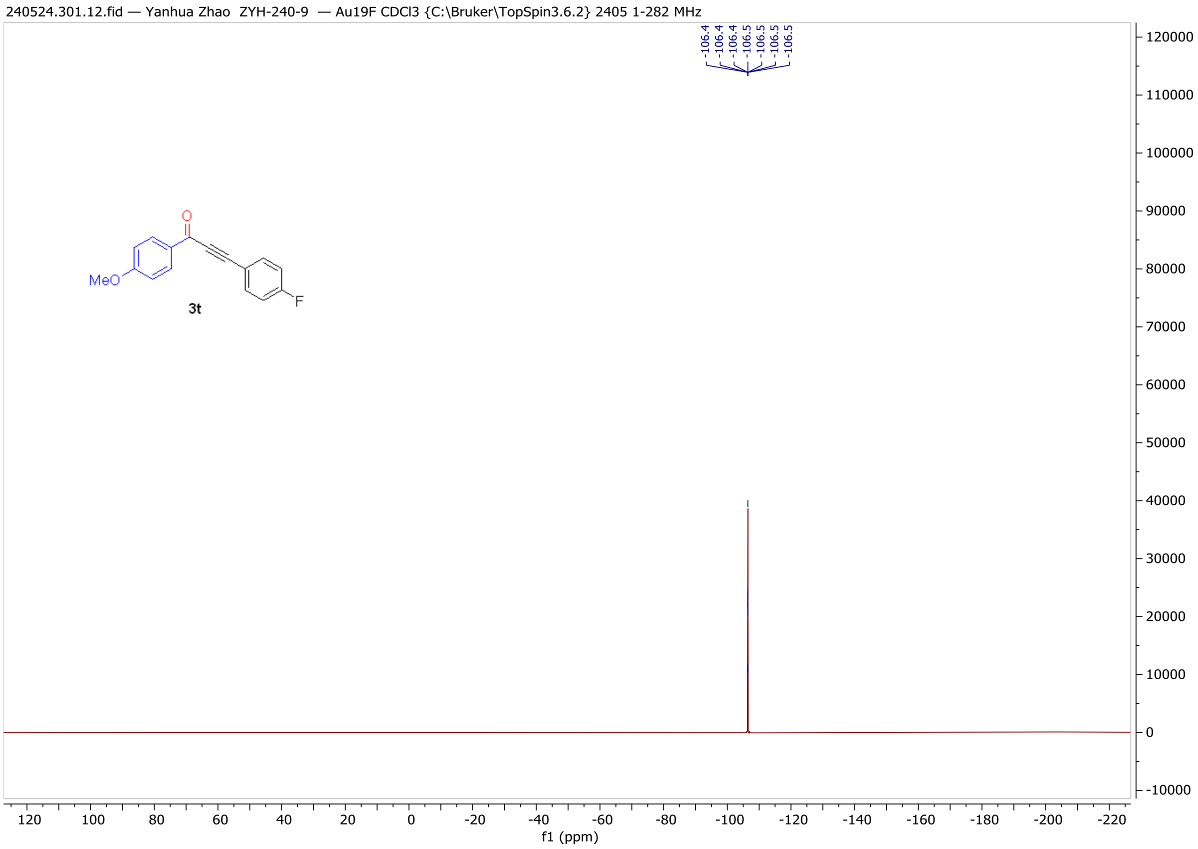
240524.301.12.fid — Yanhua Zhao ZYH-240-9 — Au19F CDCl3 {C:\Bruker\TopSpin3.6.2} 2405 1-282 MHz
-106.4
-106.4
-106.4
-106.5
-106.5
-106.5
-106.5
O
MeO
F
3t
f1 (ppm)

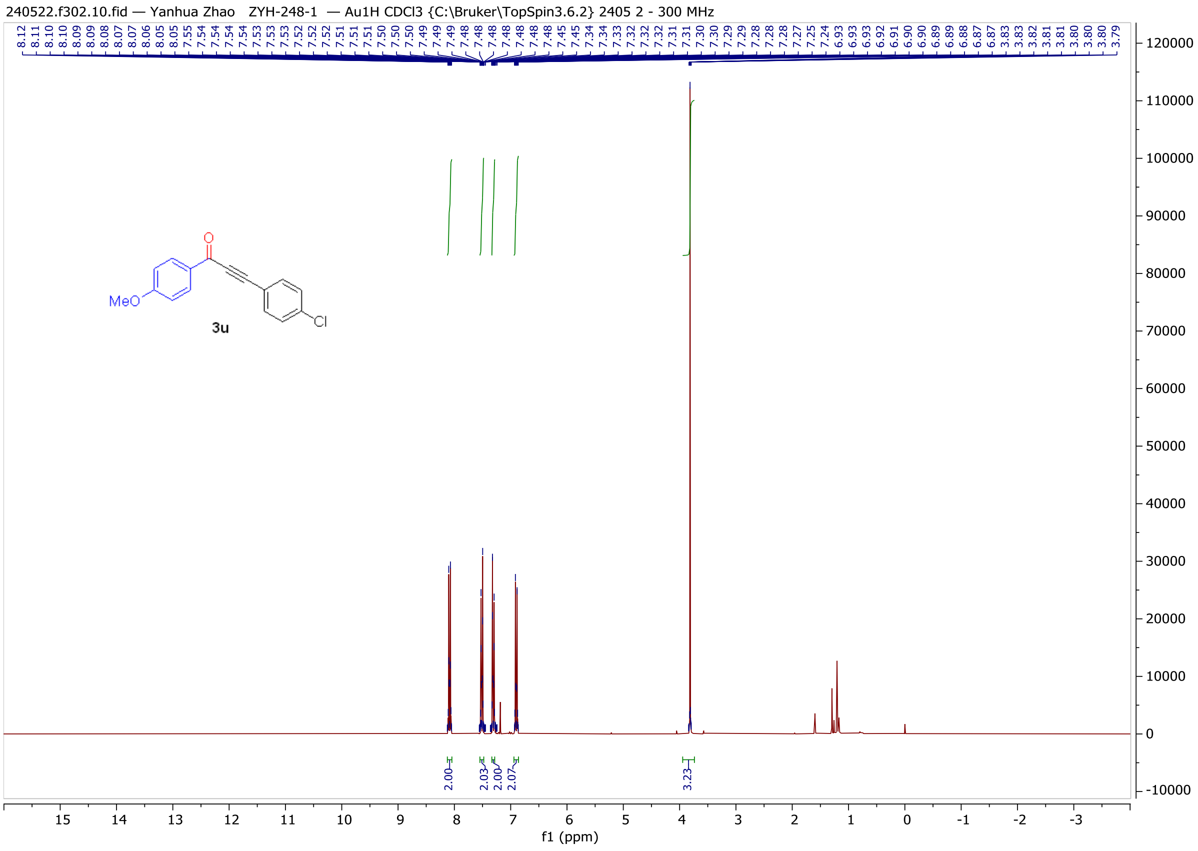
240522.f302.10.fid — Yanhua Zhao ZYH-248-1 — Au1H CDCl3 {C:\Bruker\TopSpin3.6.2} 2405 2 - 300 MHz
8.12 8.11 8.10 8.10 8.09 8.09 8.08 8.07 8.07 8.06 8.05 8.05 7.55 7.54 7.54 7.54 7.54 7.53 7.53 7.53 7.52 7.52 7.52 7.51 7.51 7.51 7.50 7.50 7.50 7.49 7.49 7.49 7.48 7.48 7.48 7.48 7.48 7.48 7.48 7.45 7.45 7.34 7.34 7.33 7.32 7.32 7.32 7.31 7.31 7.30 7.30 7.29 7.29 7.28 7.28 7.28 7.27 7.25 7.24 6.93 6.93 6.93 6.92 6.92 6.91 6.90 6.90 6.89 6.89 6.88 6.87 6.87 3.83 3.83 3.82 3.81 3.81 3.80 3.80 3.80 3.79
O
MeO
Cl
3u
2.00
2.03
2.00
2.07
3.23
f1 (ppm)

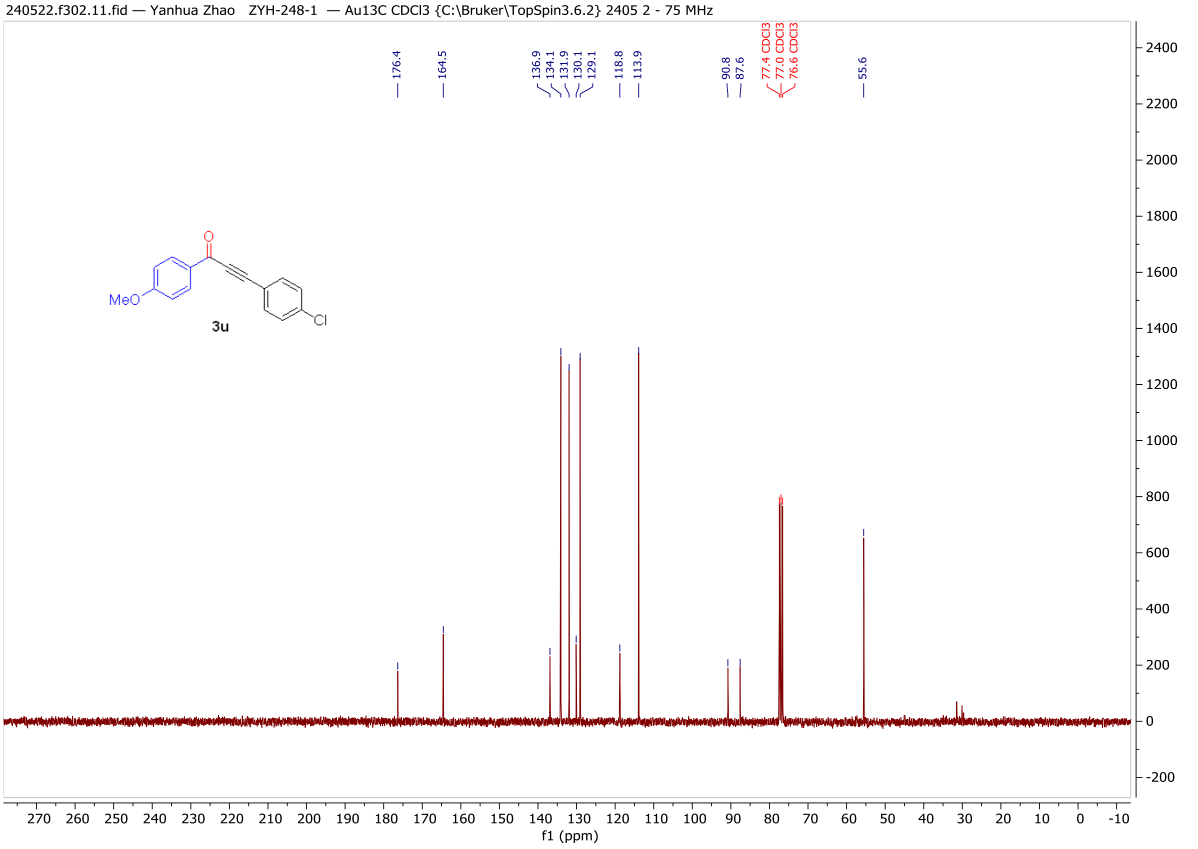
240522.f302.11.fid — Yanhua Zhao ZYH-248-1 — Au13C CDCl3 {C:\Bruker\TopSpin3.6.2} 2405 2 - 75 MHz
176.4
164.5
136.9
134.1
131.9
130.1
129.1
118.8
113.9
90.8
87.6
77.4 CDCl3
77.0 CDCl3
76.6 CDCl3
55.6
O
MeO
Cl
3u
f1 (ppm)

240516.f312.10.fid — Yanhua Zhao ZYH-240-5 — Au1H CDCl3 {C:\Bruker\TopSpin3.6.2} 2405 12 - 300 MHz

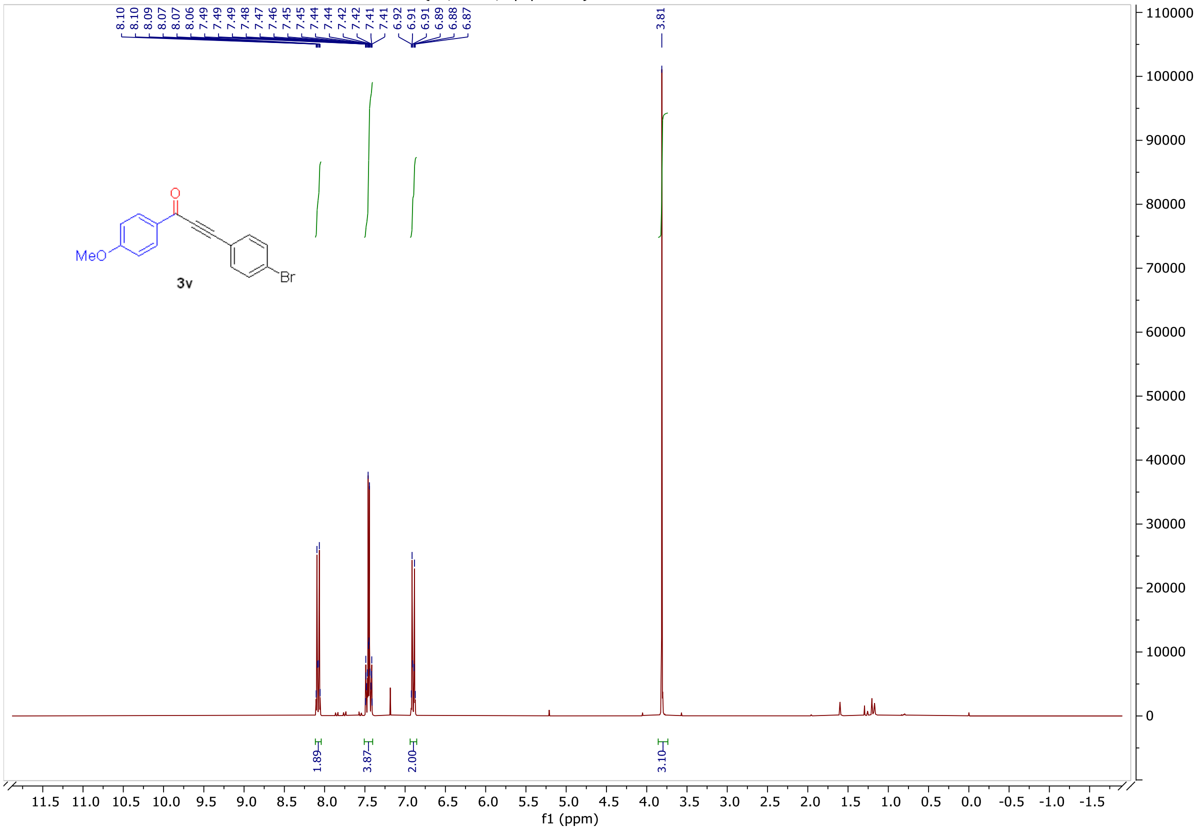

240516.f312.11.fid — Yanhua Zhao ZYH-240-5 — Au13C CDCl3 {C:\Bruker\TopSpin3.6.2} 2405 12 - 75 MHz

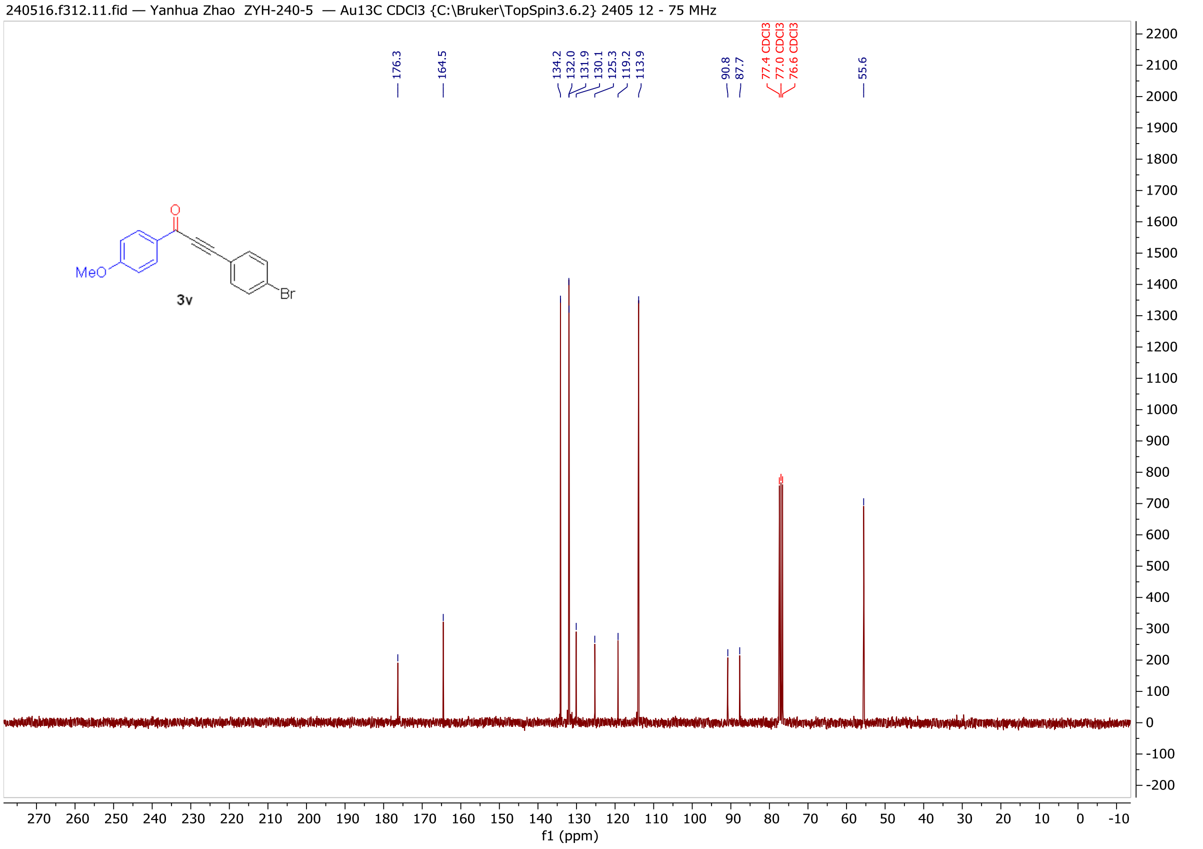

240516.f310.10.fid — Yanhua Zhao ZYH-240-2 — Au1H CDCl3 {C:\Bruker\TopSpin3.6.2} 2405 10 - 300 MHz

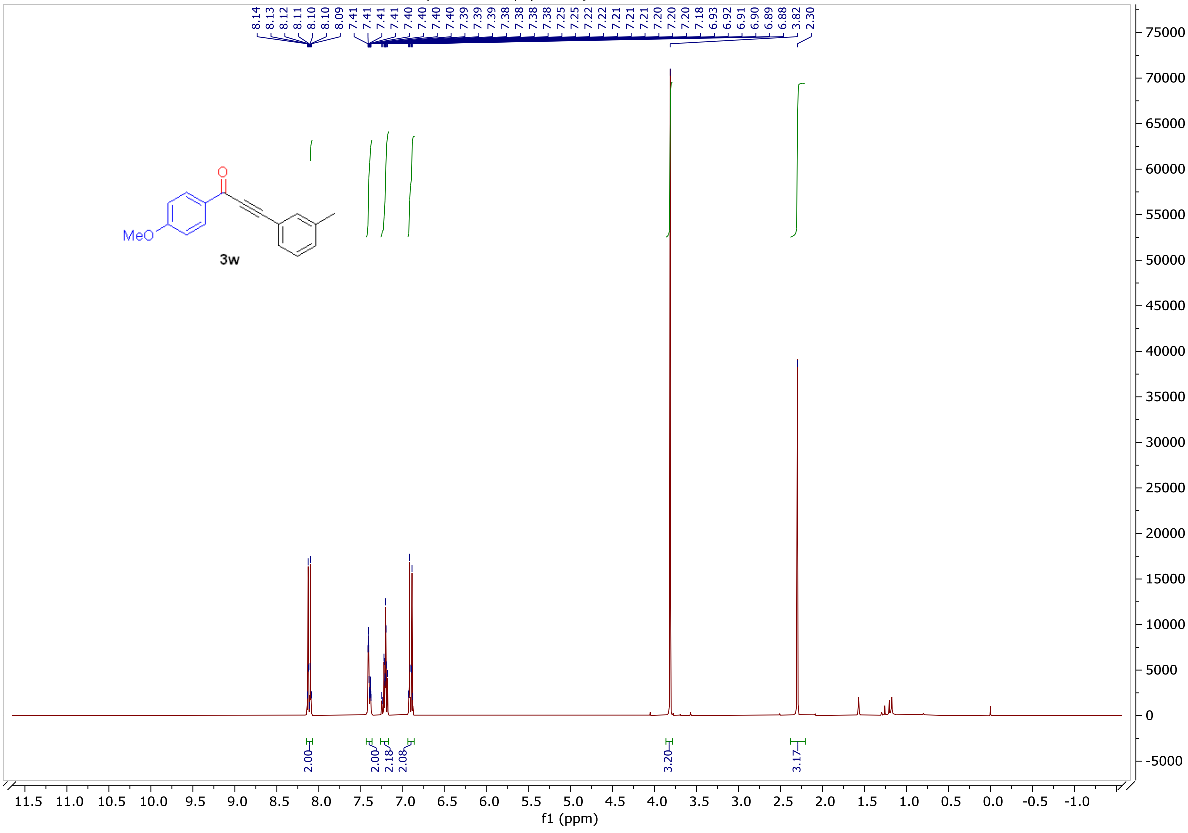

240516.f310.11.fid — Yanhua Zhao ZYH-240-2 — Au13C CDCl3 {C:\Bruker\TopSpin3.6.2} 2405 10 - 75 MHz

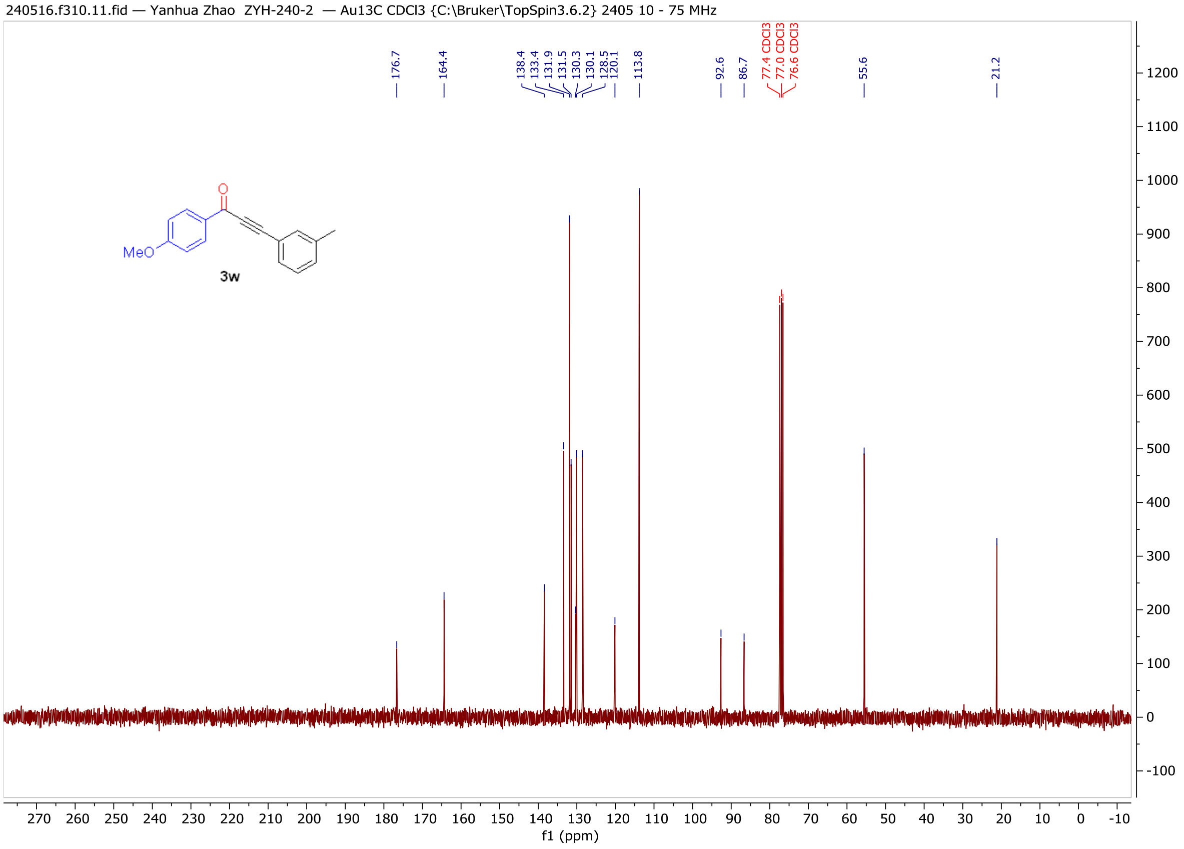

240522.f308.10.fid — Yanhua Zhao ZYH-240-12 — Au1H CDCl3 {C:\Bruker\TopSpin3.6.2} 2405 8 - 300 MHz

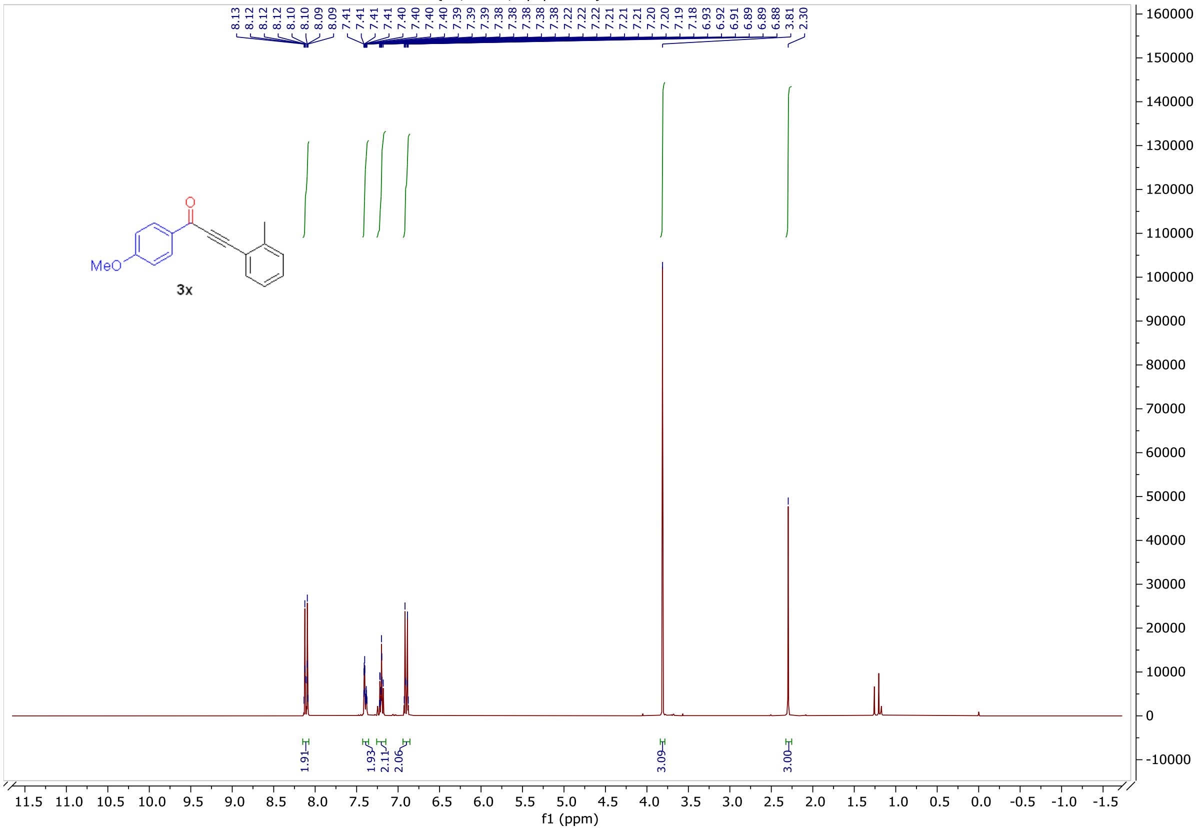

240522.f308.11.fid — Yanhua Zhao ZYH-240-12 — Au13C CDCl3 {C:\Bruker\TopSpin3.6.2} 2405 8 - 75 MHz

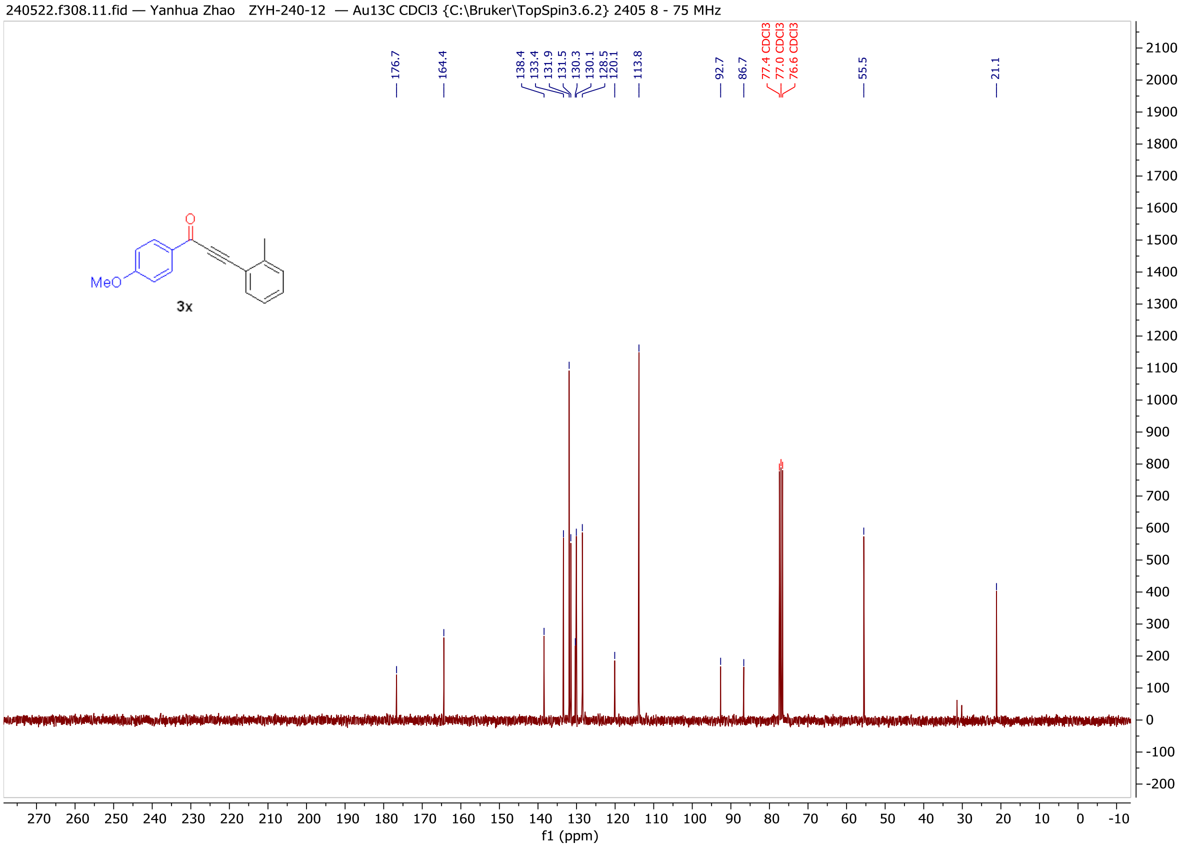

240530.308.10.fid — Yanhua Zhao ZYH-248-8 — Au1H CDCl3 {C:\Bruker\TopSpin3.6.2} 2405 8 - 300 MHz

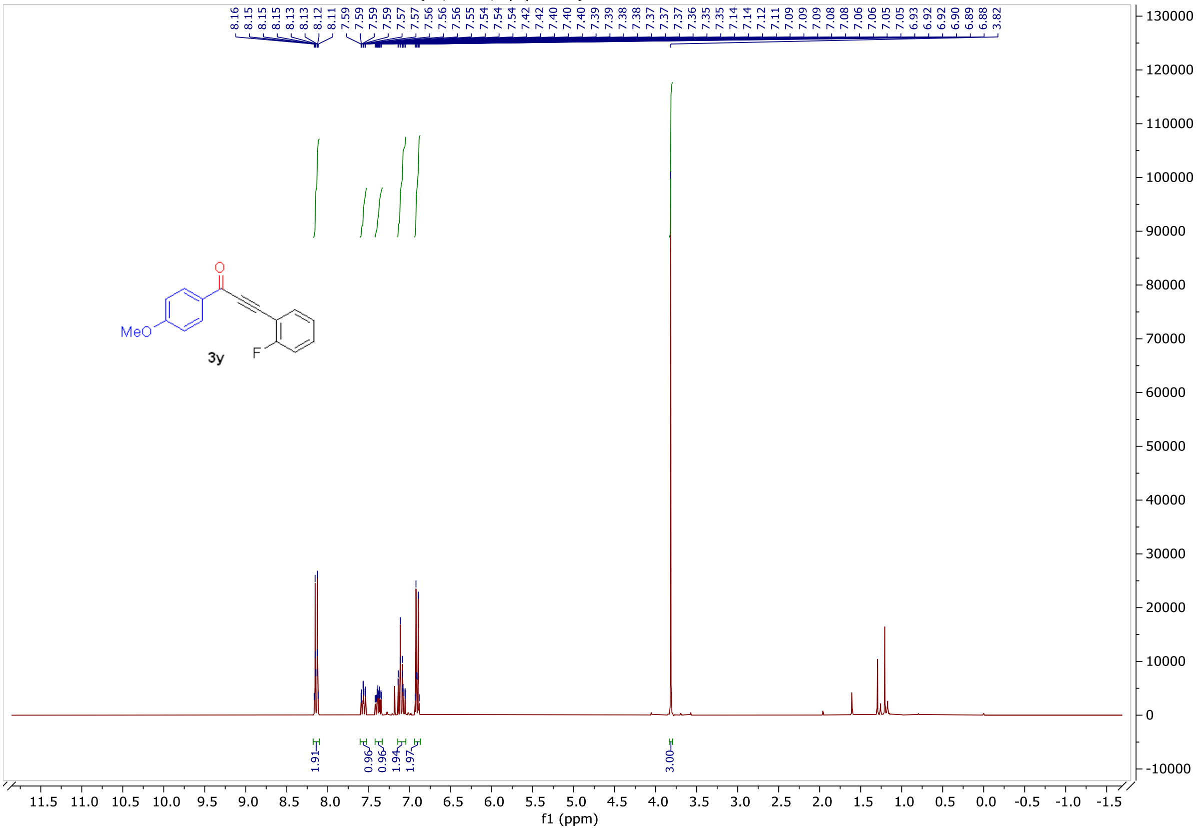

240530.308.11.fid — Yanhua Zhao ZYH-248-8 — Au13C CDCl3 {C:\Bruker\TopSpin3.6.2} 2405 8 - 75 MHz

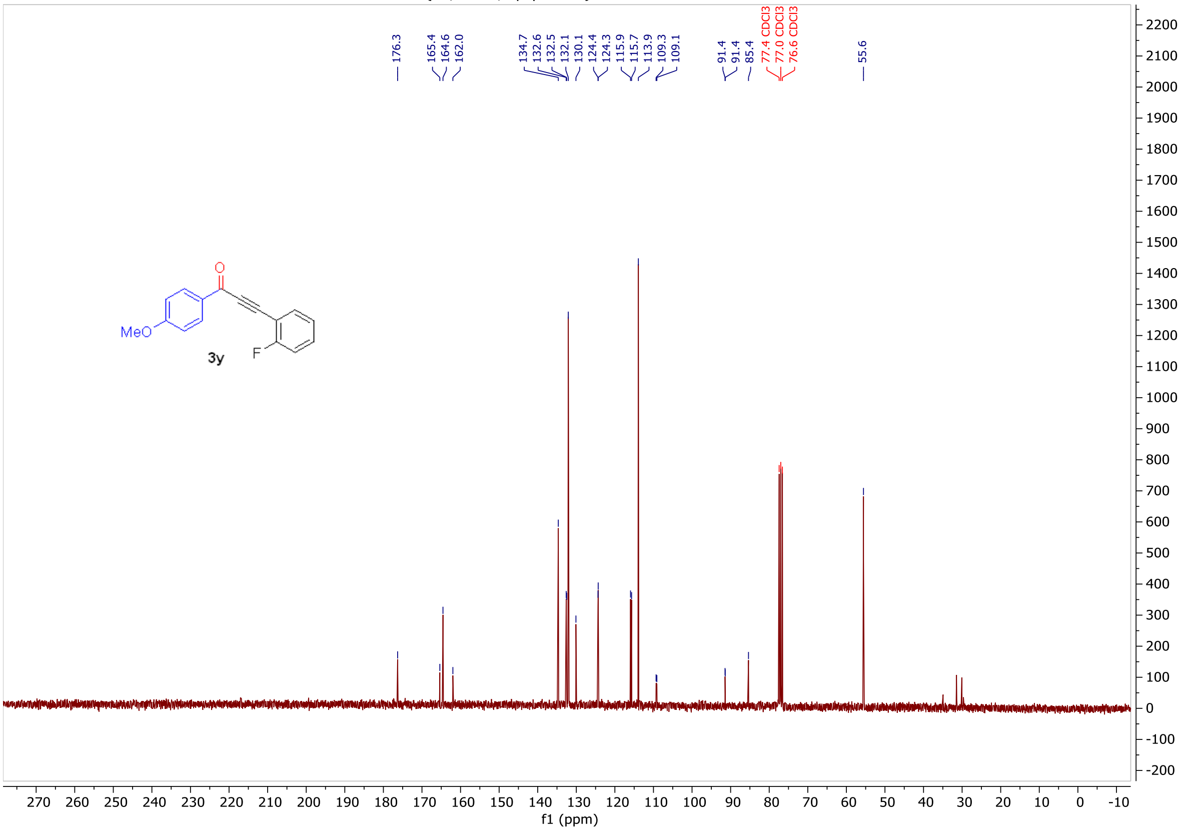

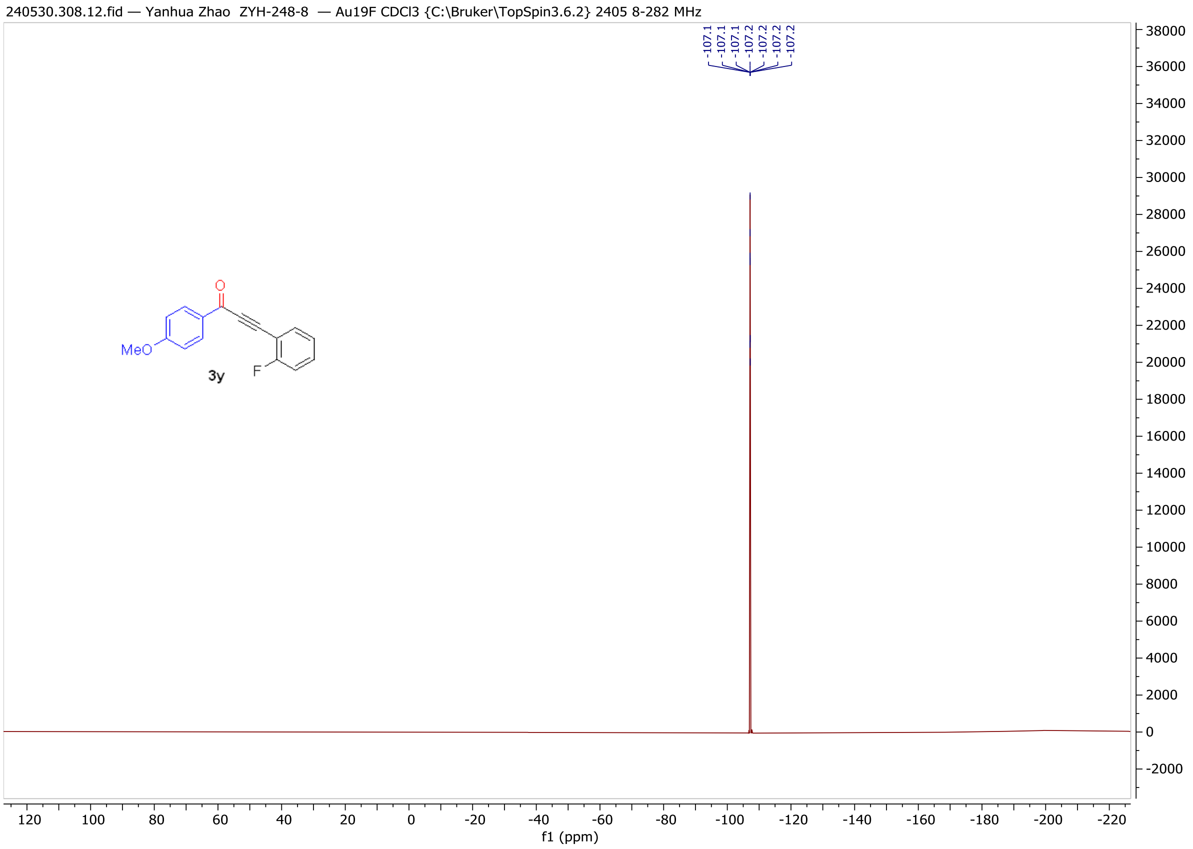
240530.308.12.fid — Yanhua Zhao ZYH-248-8 — Au19F CDCl3 {C:\Bruker\TopSpin3.6.2} 2405 8-282 MHz
-107.1
-107.1
-107.1
-107.2
-107.2
-107.2
-107.2
MeO
O
F
3y
f1 (ppm)

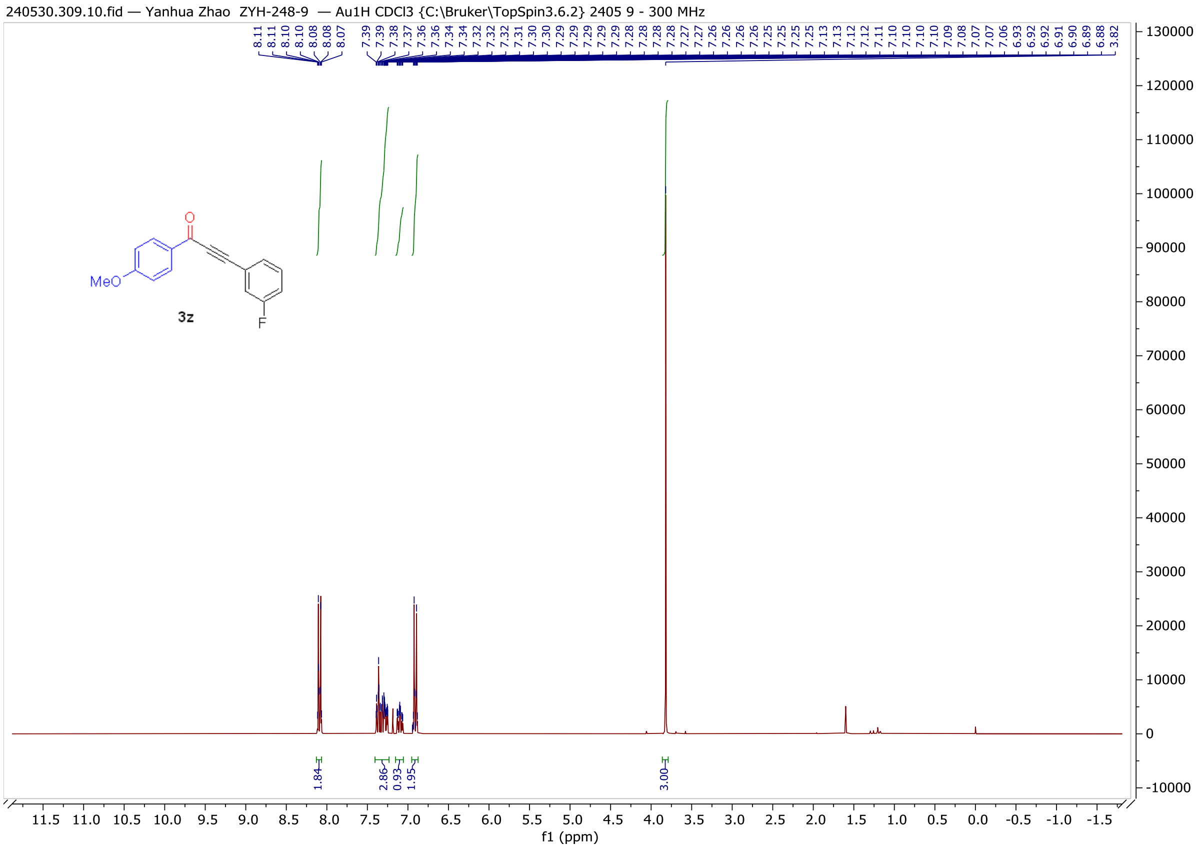
240530.309.10.fid — Yanhua Zhao ZYH-248-9 — Au1H CDCl3 {C:\Bruker\TopSpin3.6.2} 2405 9 - 300 MHz
MeO
O
F
3z
f1 (ppm)

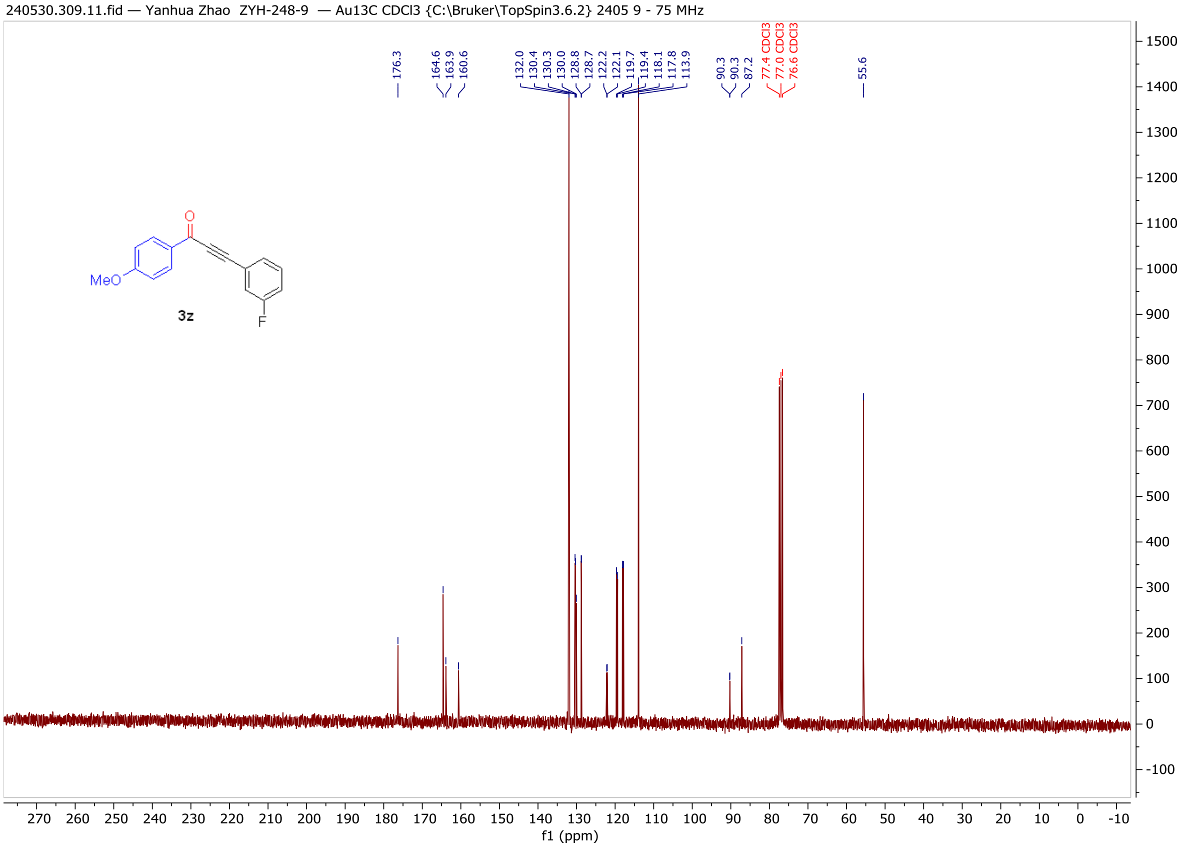
240530.309.11.fid — Yanhua Zhao ZYH-248-9 — Au13C CDCl3 {C:\Bruker\TopSpin3.6.2} 2405 9 - 75 MHz
176.3
164.6
163.9
160.6
132.0
130.4
130.3
130.0
128.8
128.7
122.2
122.1
119.7
119.4
118.1
117.8
113.9
90.3
90.3
87.2
77.4 CDCl3
77.0 CDCl3
76.6 CDCl3
55.6
MeO
O
F
3z
f1 (ppm)

240530.309.12.fid — Yanhua Zhao ZYH-248-9 — Au19F CDCl3 {C:\Bruker\TopSpin3.6.2} 2405 9-282 MHz

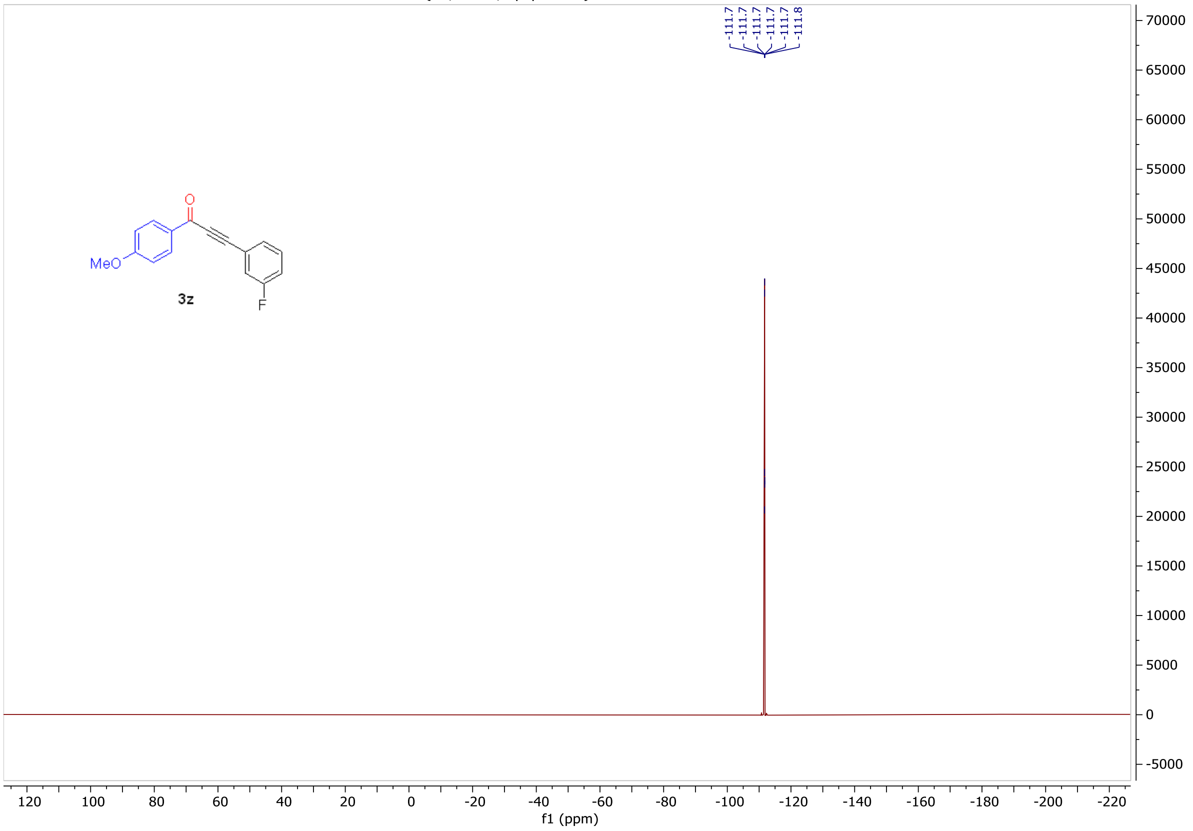

240516.f313.10.fid — Yanhua Zhao ZYH-240-4 — Au1H CDCl3 {C:\Bruker\TopSpin3.6.2} 2405 13 - 300 MHz

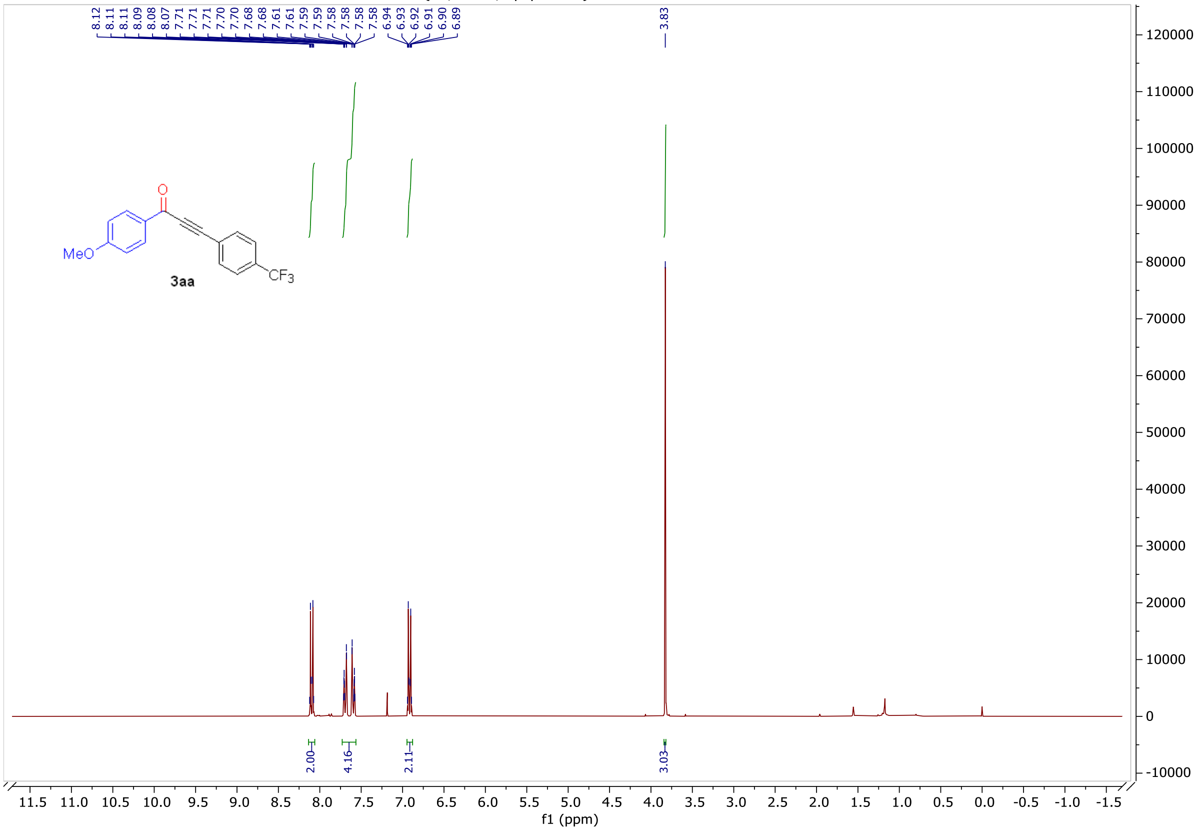

240516.f313.11.fid — Yanhua Zhao ZYH-240-4 — Au13C CDCl3 {C:\Bruker\TopSpin3.6.2} 2405 13 - 75 MHz

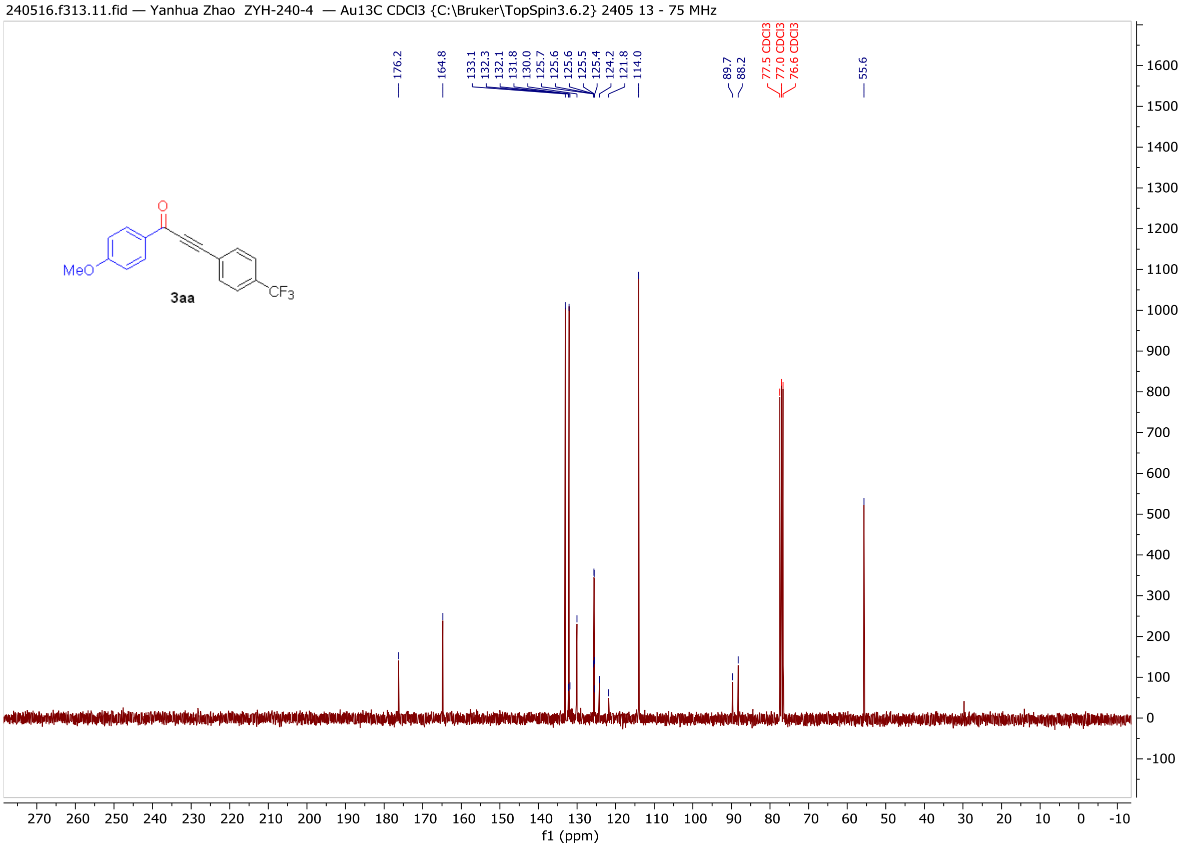

240516.f313.12.fid — Yanhua Zhao ZYH-240-4 — Au19F(H-gek) CDCl3 {C:\Bruker\TopSpin3.6.2} 2405 13-282 MHz

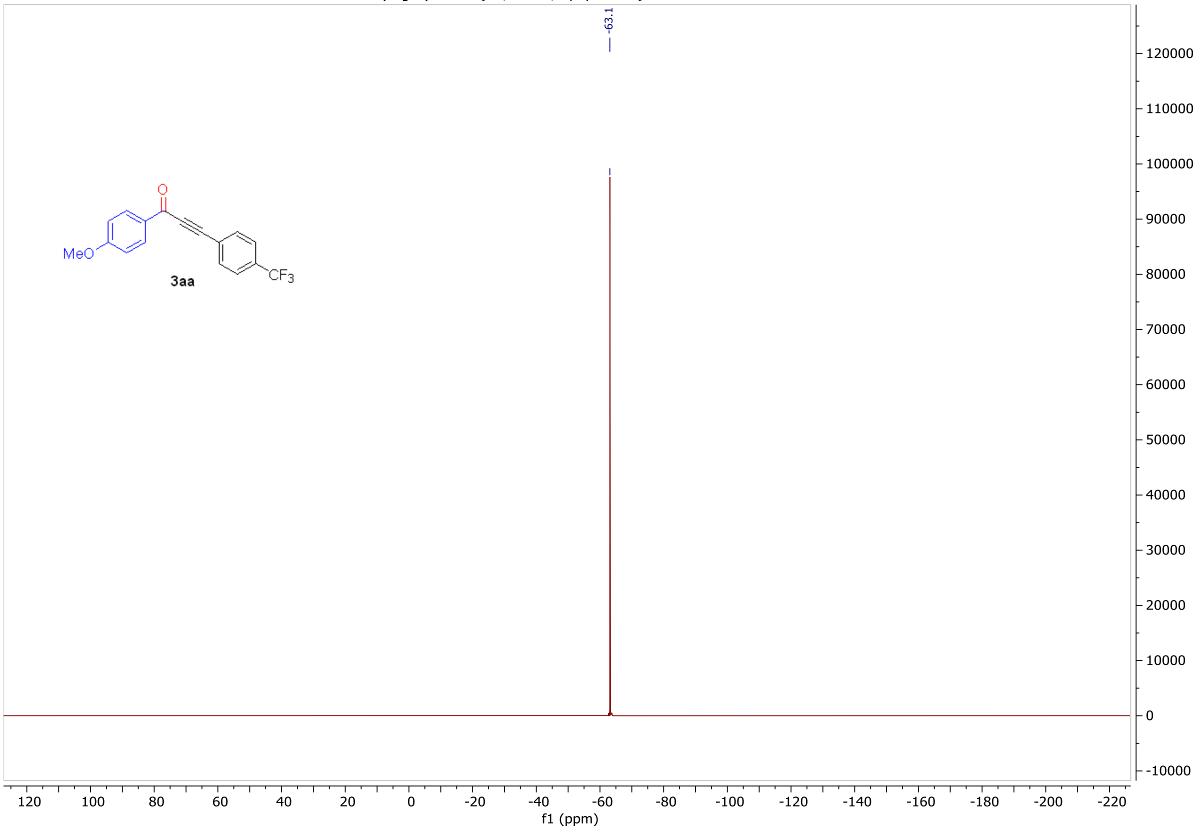

240522.f304.10.fid — Yanhua Zhao ZYH-248-3 — Au1H CDCl3 {C:\Bruker\TopSpin3.6.2} 2405 4 - 300 MHz

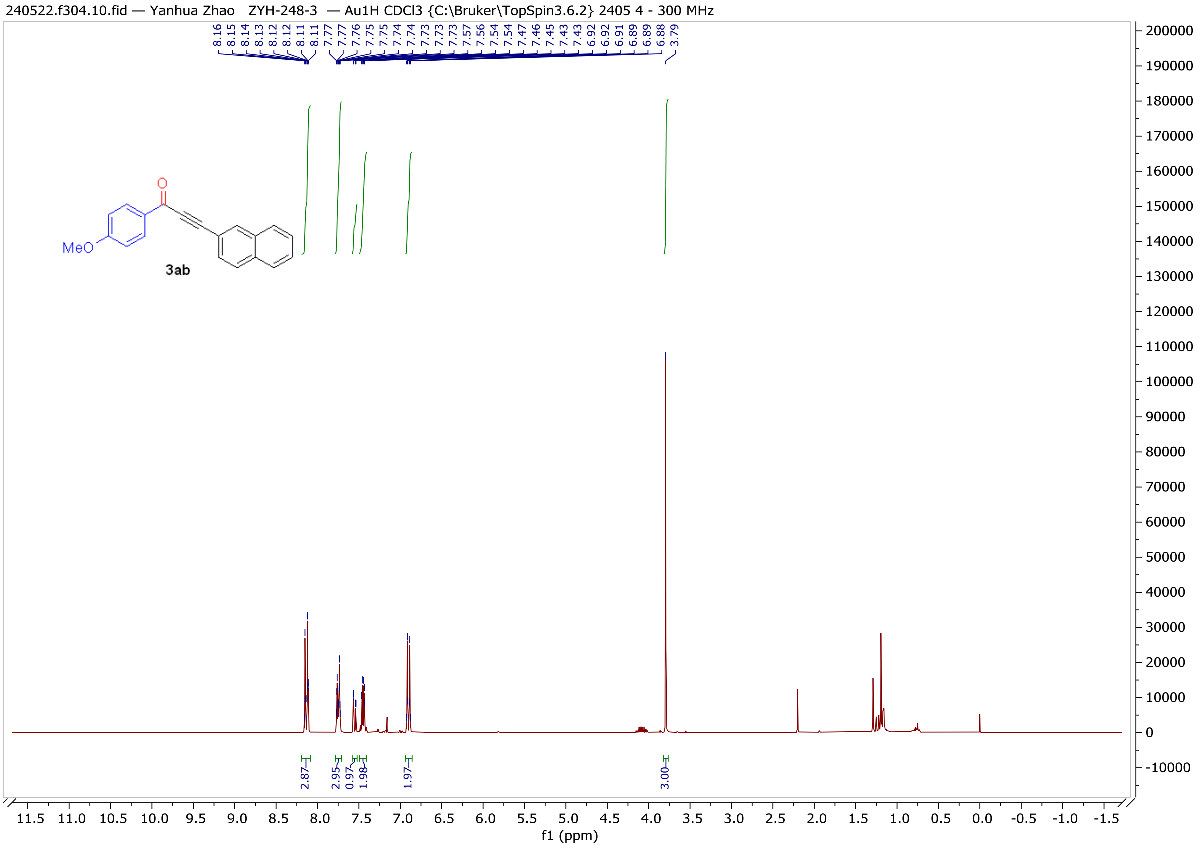

240522.f304.11.fid — Yanhua Zhao ZYH-248-3 — Au13C CDCl3 {C:\Bruker\TopSpin3.6.2} 2405 4 - 75 MHz

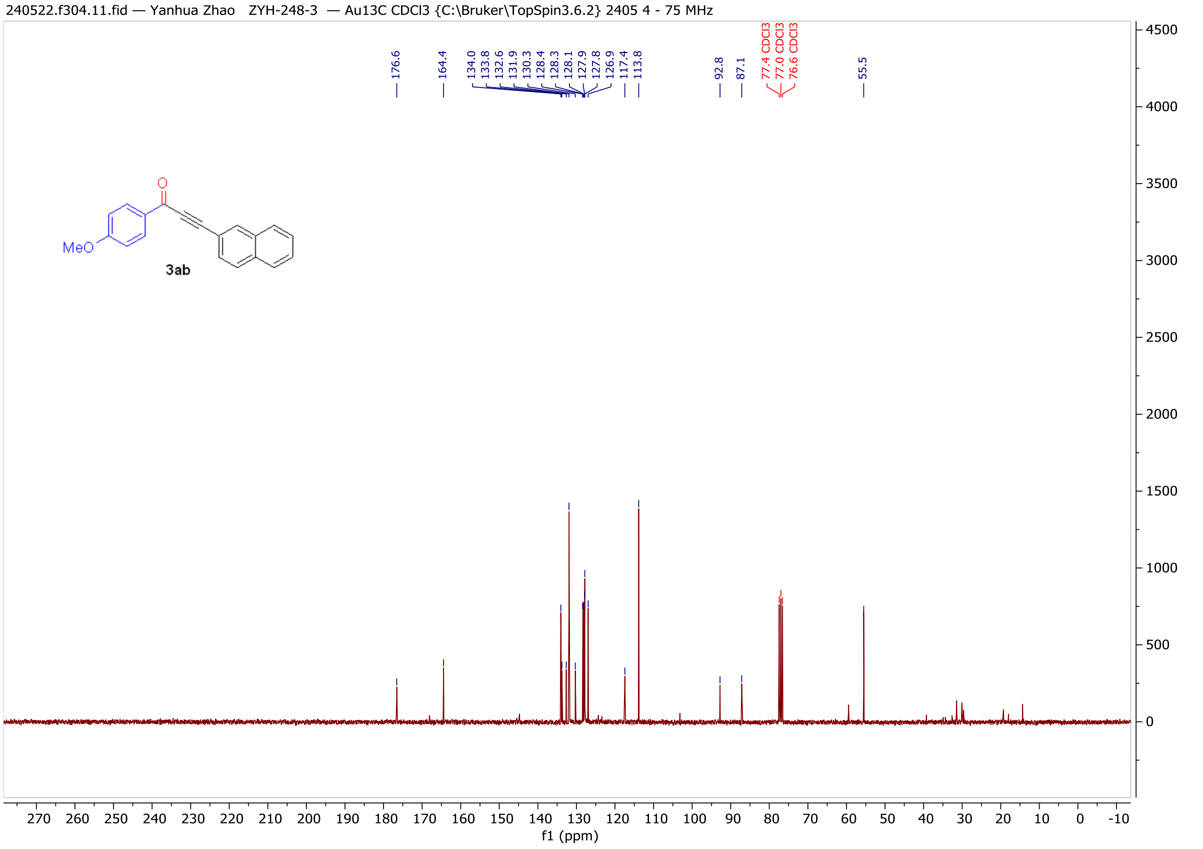

240516.f311.10.fid — Yanhua Zhao ZYH-240-3 — Au1H CDCl3 {C:\Bruker\TopSpin3.6.2} 2405 11 - 300 MHz

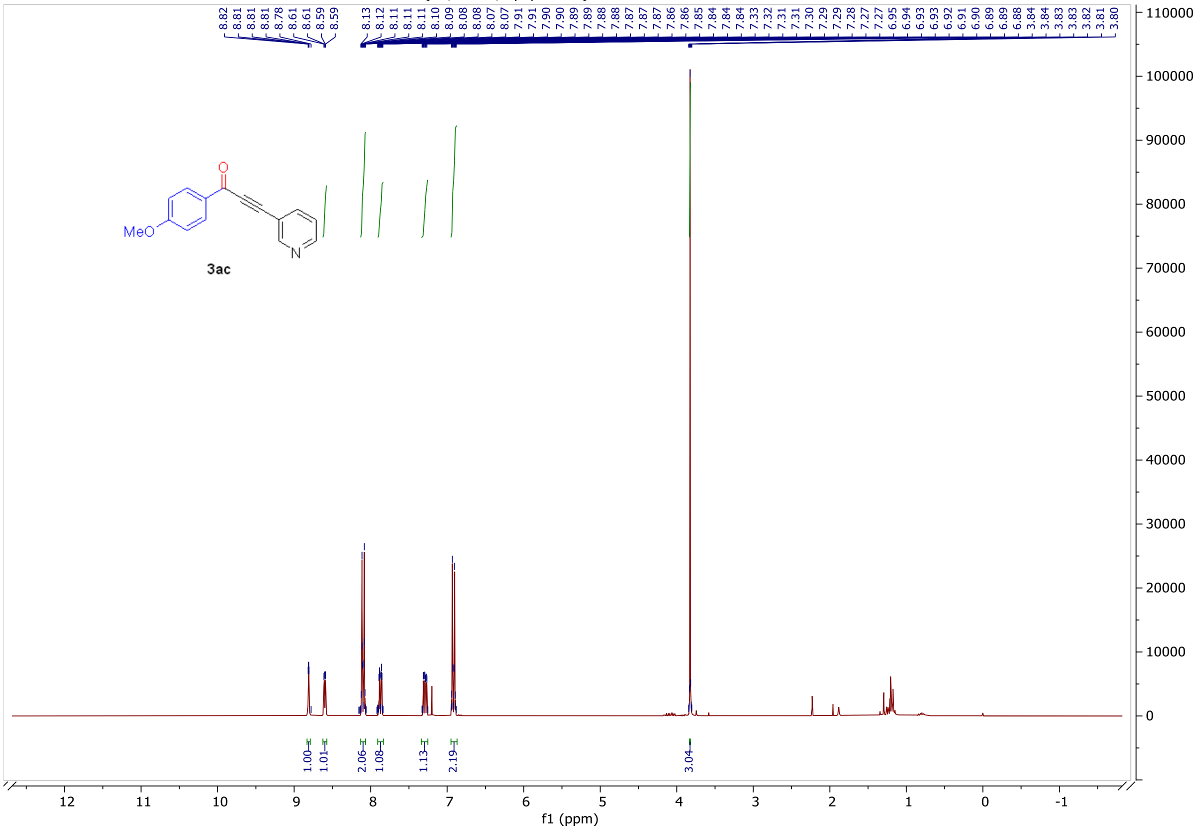

240516.f311.11.fid — Yanhua Zhao ZYH-240-3 — Au13C CDCl3 {C:\Bruker\TopSpin3.6.2} 2405 11 - 75 MHz

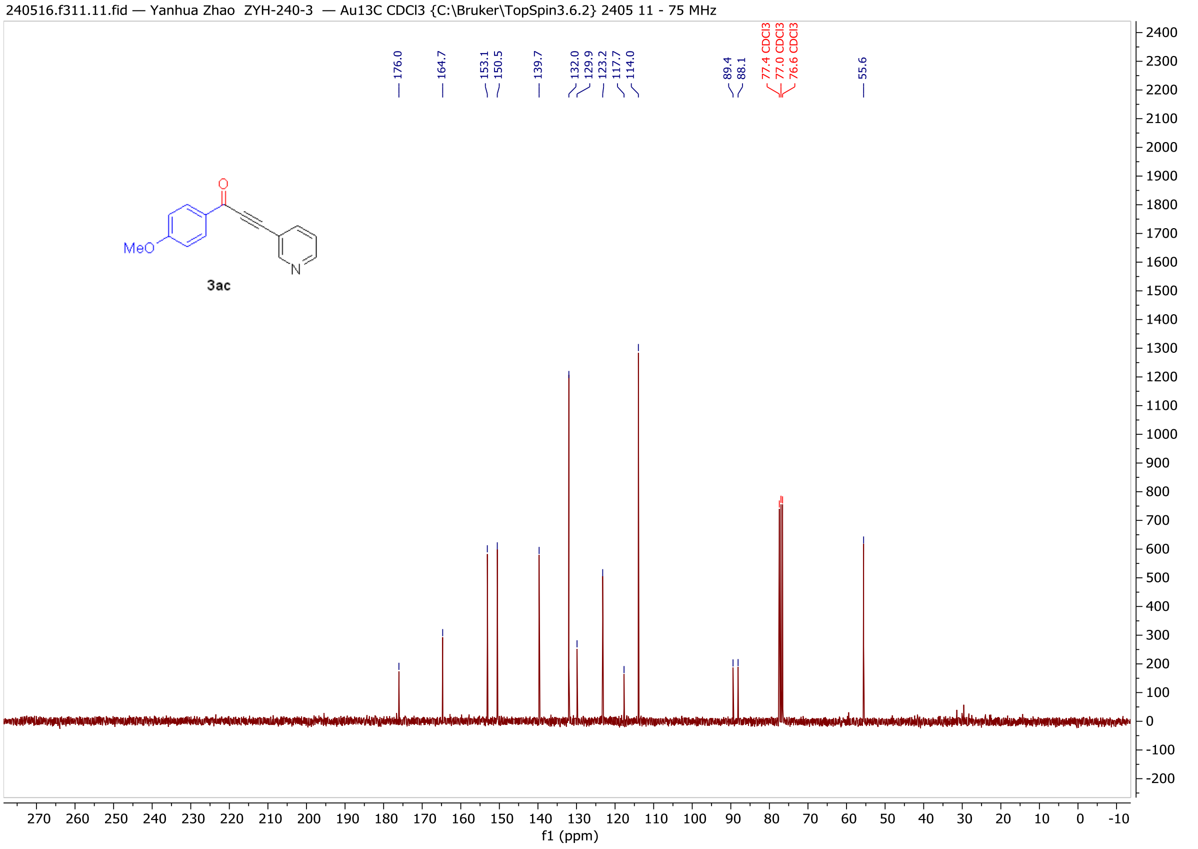

240522.f307.10.fid — Yanhua Zhao ZYH-240-10 — Au1H CDCl3 {C:\Bruker\TopSpin3.6.2} 2405 7 - 300 MHz

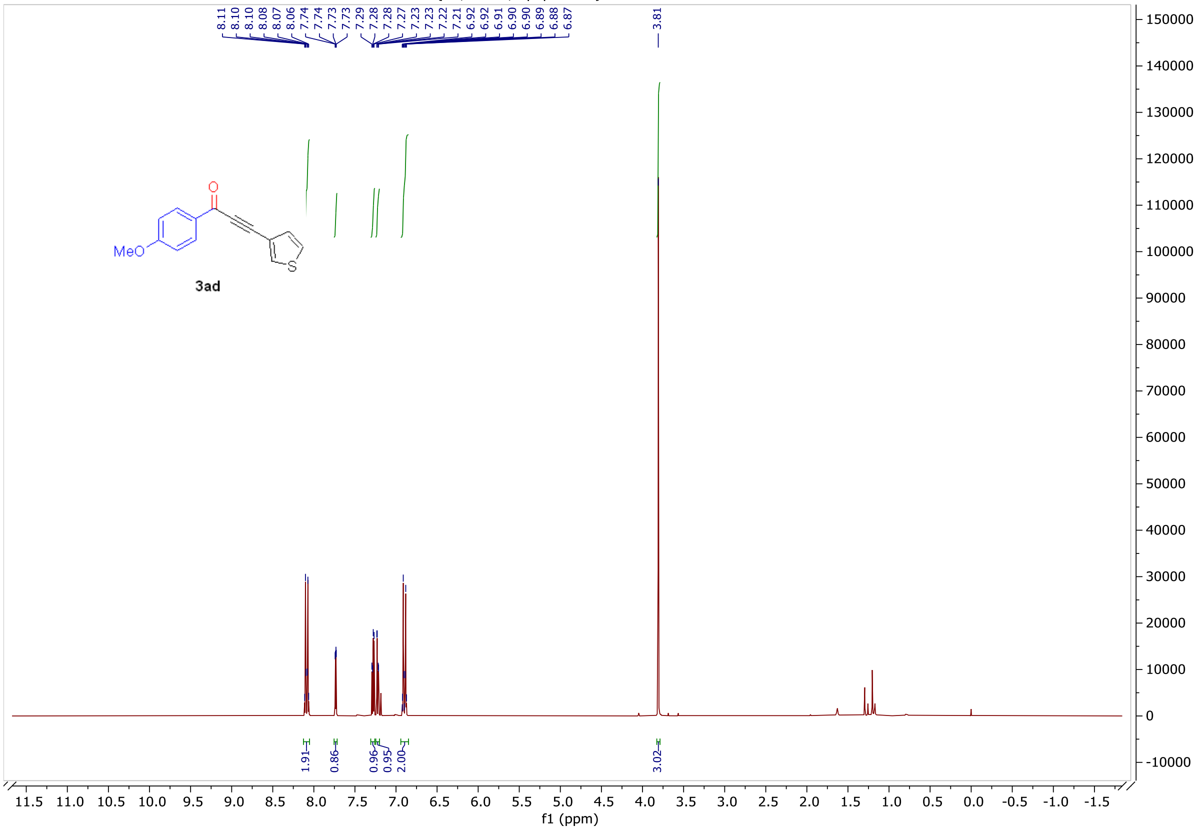

240522.f307.11.fid — Yanhua Zhao ZYH-240-10 — Au13C CDCl3 {C:\Bruker\TopSpin3.6.2} 2405 7 - 75 MHz

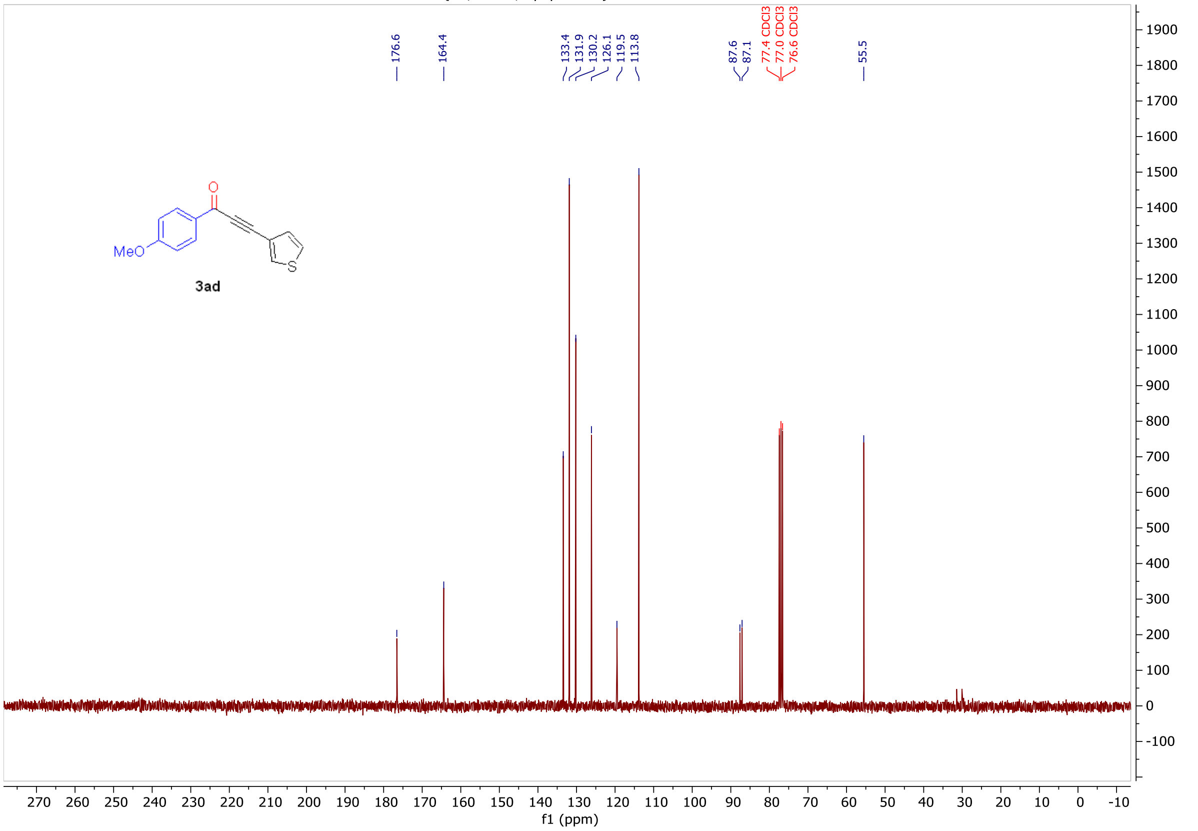

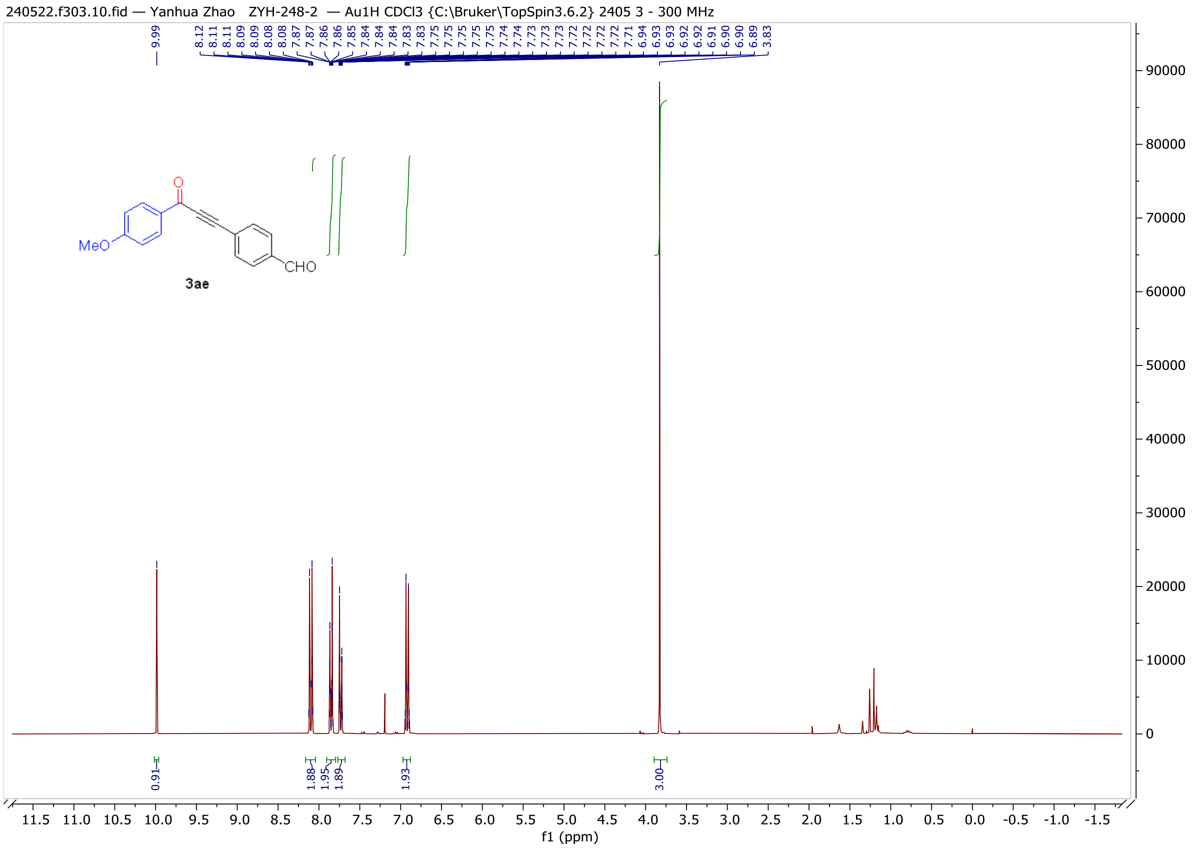
240522.f303.10.fid — Yanhua Zhao ZYH-248-2 — Au1H CDCl3 {C:\Bruker\TopSpin3.6.2} 2405 3 - 300 MHz
3ae
MeO
CHO
f1 (ppm)

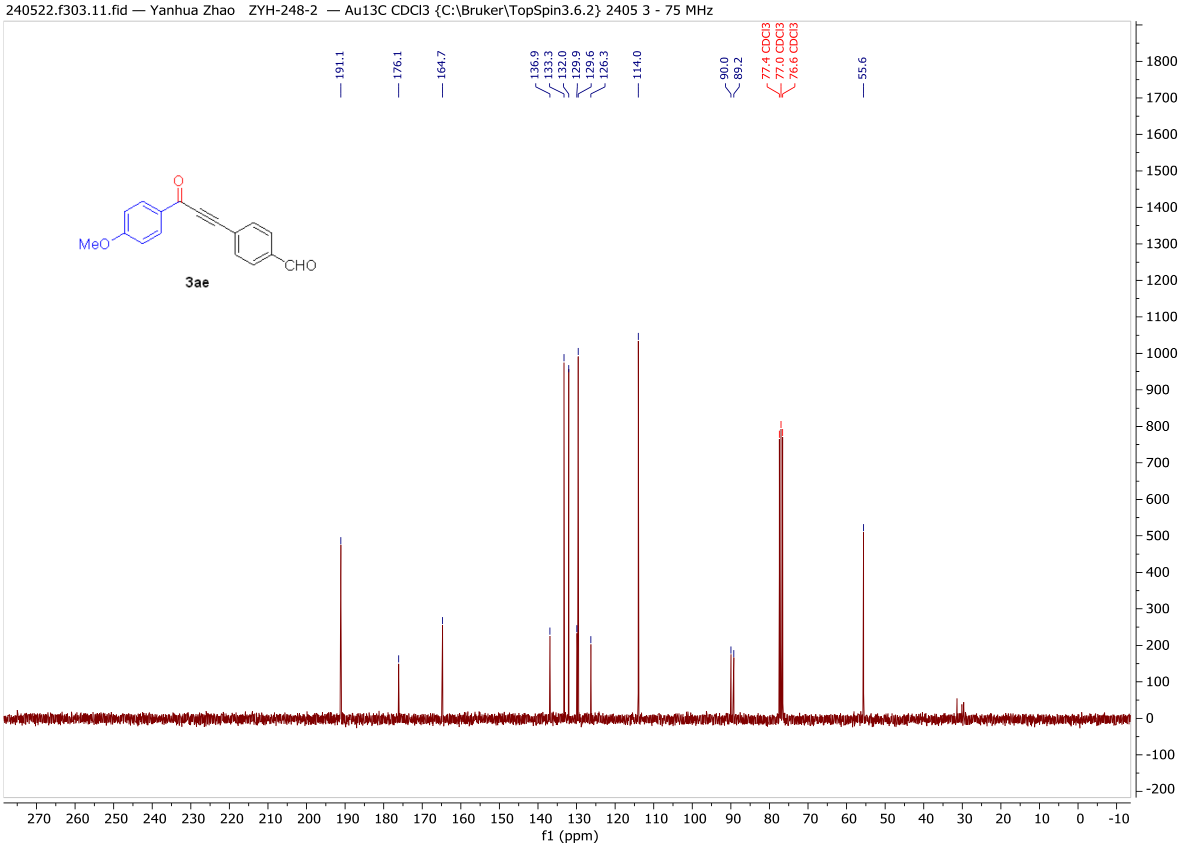
240522.f303.11.fid — Yanhua Zhao ZYH-248-2 — Au13C CDCl3 {C:\Bruker\TopSpin3.6.2} 2405 3 - 75 MHz
191.1
176.1
164.7
136.9
133.3
132.0
129.9
129.6
126.3
114.0
90.0
89.2
77.4 CDCl3
77.0 CDCl3
76.6 CDCl3
55.6
3ae
MeO
CHO
f1 (ppm)

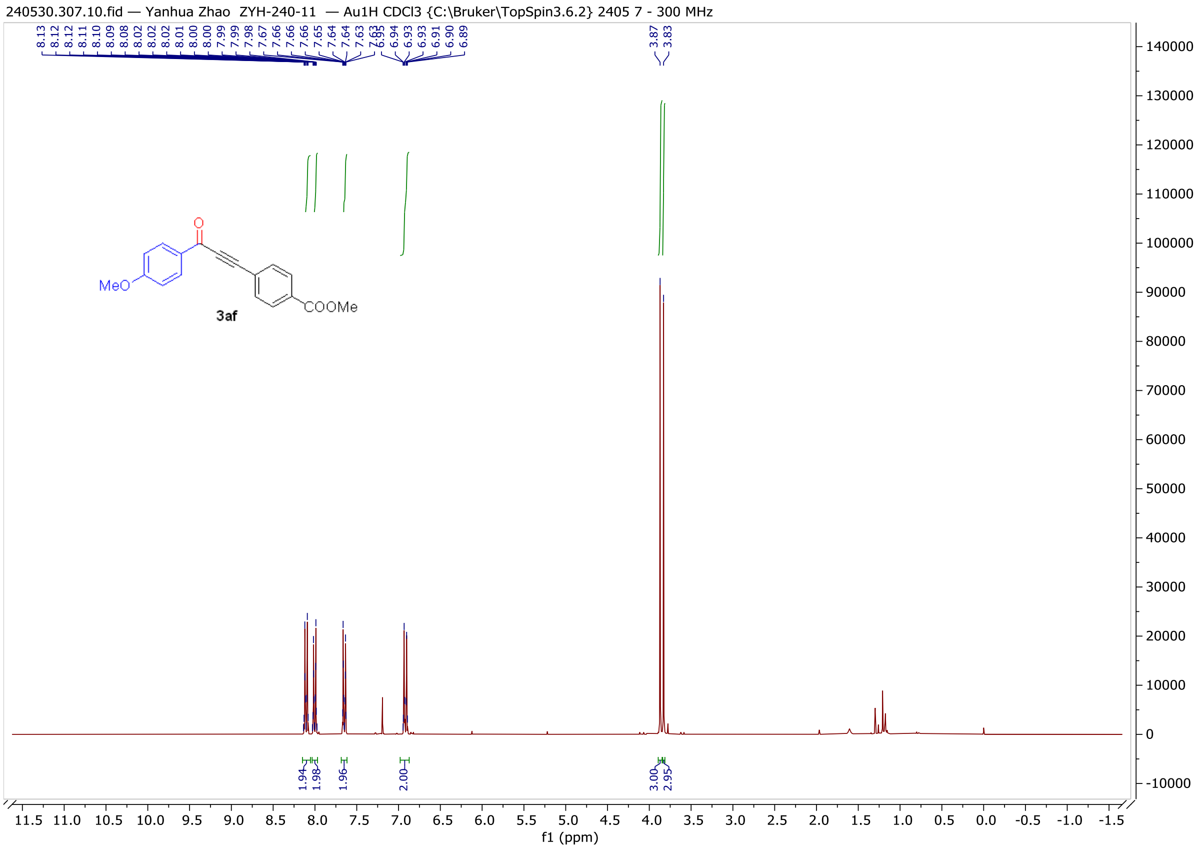
240530.307.10.fid — Yanhua Zhao ZYH-240-11 — Au1H CDCl3 {C:\Bruker\TopSpin3.6.2} 2405 7 - 300 MHz
8.13 8.12 8.12 8.11 8.10 8.09 8.08 8.02 8.02 8.02 8.01 8.00 8.00 7.99 7.99 7.98 7.67 7.66 7.66 7.65 7.64 7.64 7.63 7.63 6.95 6.94 6.93 6.93 6.91 6.90 6.89
3.87 3.83
MeO
O
COOMe
3af
1.94 1.98 1.96 2.00 3.00 2.95
f1 (ppm)

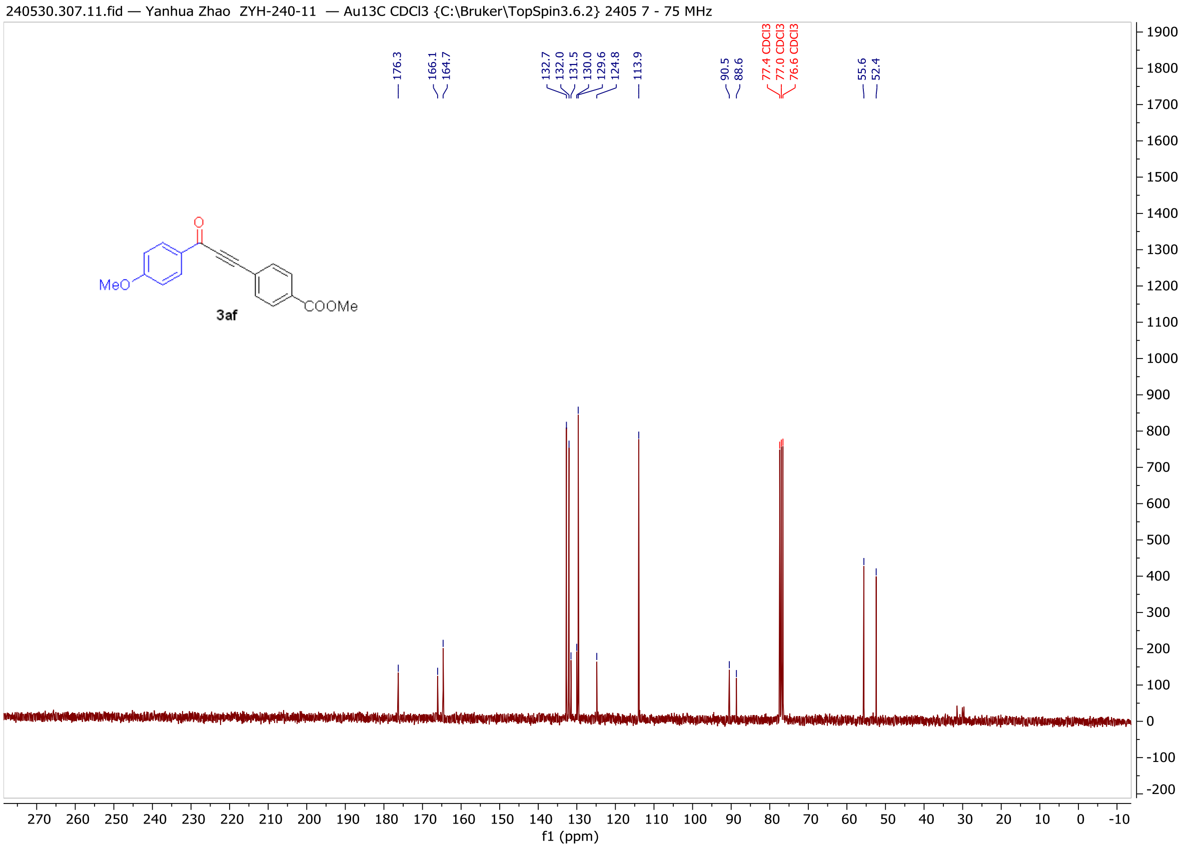
240530.307.11.fid — Yanhua Zhao ZYH-240-11 — Au13C CDCl3 {C:\Bruker\TopSpin3.6.2} 2405 7 - 75 MHz
176.3
166.1 164.7
132.7 132.0 131.5 130.0 129.6 124.8
113.9
90.5 88.6
77.4 CDCl3 77.0 CDCl3 76.6 CDCl3
55.6 52.4
MeO
O
COOMe
3af
f1 (ppm)

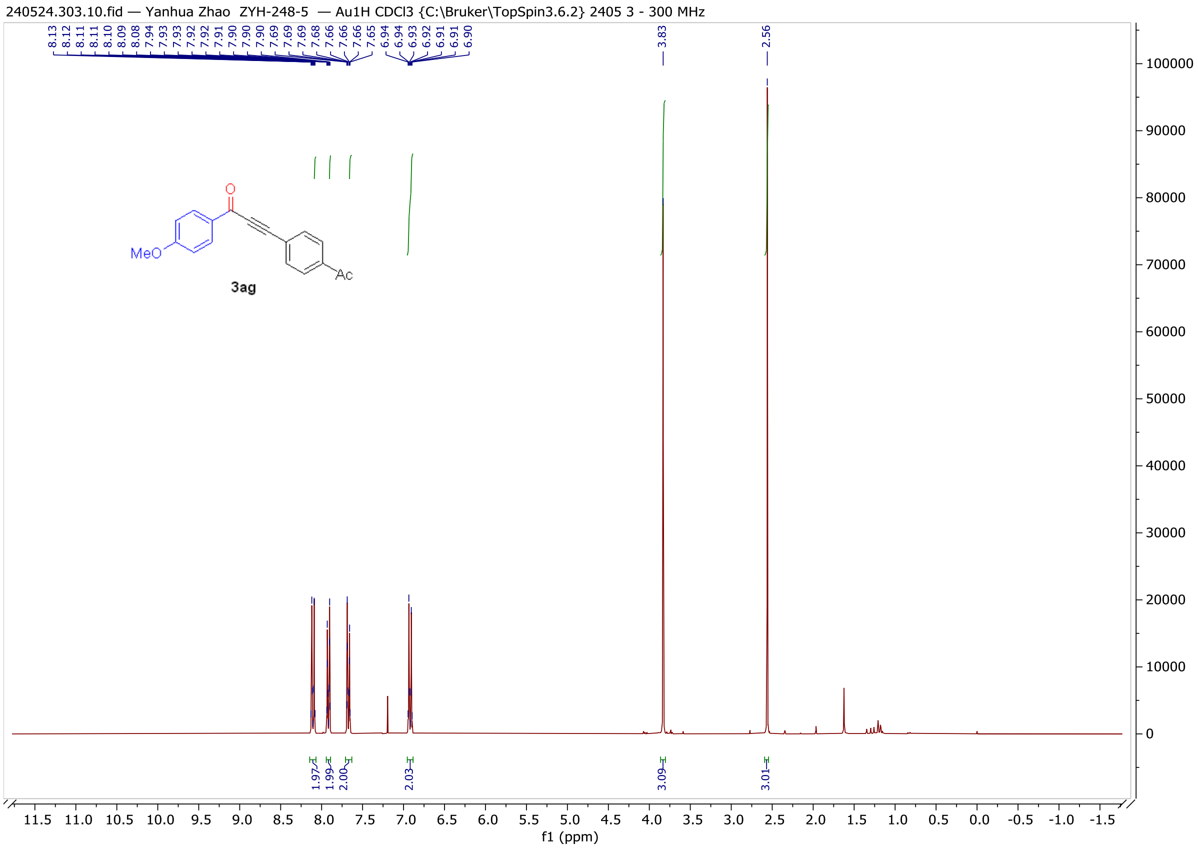
240524.303.10.fid — Yanhua Zhao ZYH-248-5 — Au1H CDCl3 {C:\Bruker\TopSpin3.6.2} 2405 3 - 300 MHz
8.13 8.12 8.11 8.10 8.09 8.08 7.94 7.93 7.93 7.92 7.92 7.91 7.90 7.90 7.90 7.69 7.69 7.69 7.68 7.66 7.66 7.66 7.65 6.94 6.94 6.93 6.92 6.91 6.91 6.90
3.83
2.56
MeO
O
Ac
3ag
1.97 1.99 2.00 2.03 3.09 3.01
f1 (ppm)

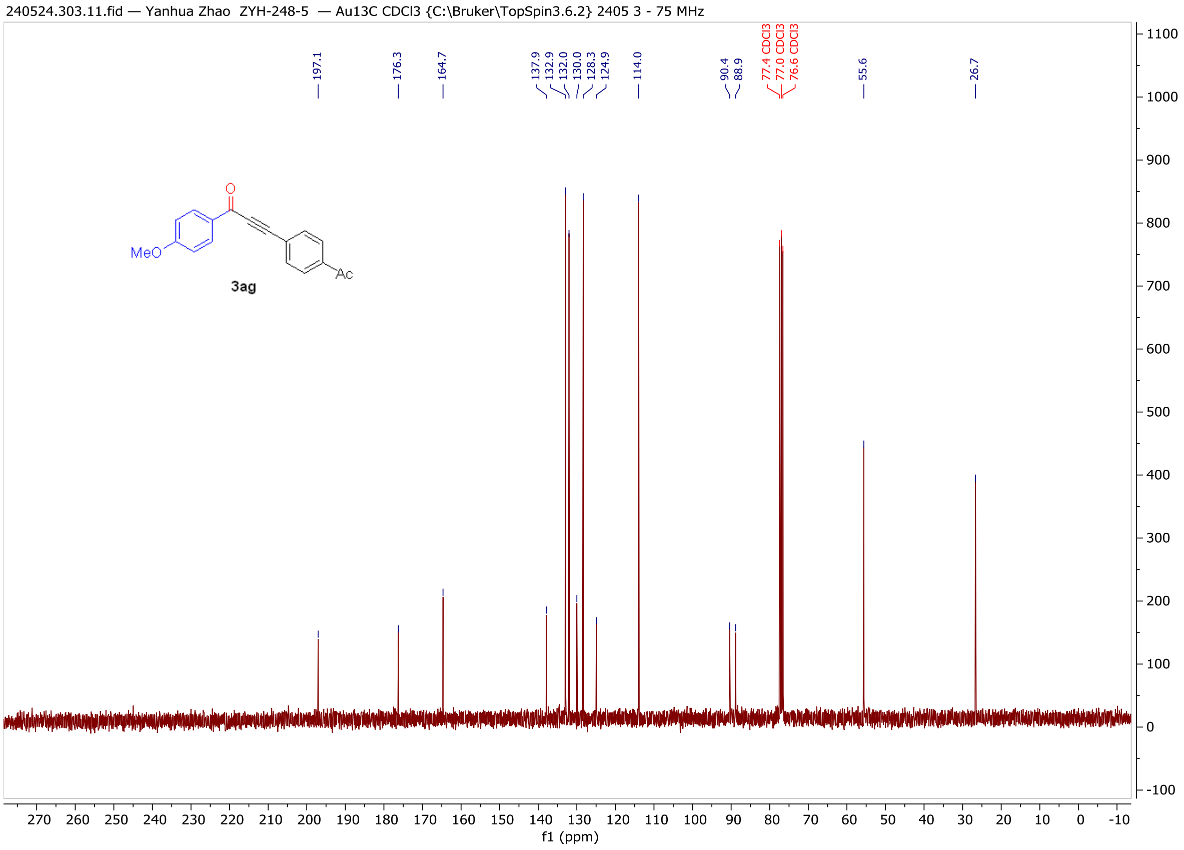
240524.303.11.fid — Yanhua Zhao ZYH-248-5 — Au13C CDCl3 {C:\Bruker\TopSpin3.6.2} 2405 3 - 75 MHz
197.1
176.3
164.7
137.9 132.9 132.0 130.0 128.3 124.9
114.0
90.4 88.9
77.4 CDCl3 77.0 CDCl3 76.6 CDCl3
55.6
26.7
MeO
O
Ac
3ag
f1 (ppm)
